# Systematic mutation analysis in rare colorectal cancer presenting ovarian metastases

**DOI:** 10.1038/s41598-019-53182-6

**Published:** 2019-11-18

**Authors:** Sungjin Park, Hee Kyung Ahn, Dae Ho Lee, YunJae Jung, Joo-Won Jeong, Seungyoon Nam, Won-Suk Lee

**Affiliations:** 1 College of Medicine, Gachon University, Incheon, Korea; 2 Gachon Institute of Genome Medicine and Science, Gachon University Gil Medical Center, Incheon, Korea; 3 Department of Medical Oncology, Gachon University Gil Medical Center, Incheon, Korea; 4 Gachon Advanced Institute of Health Sciences & Technology, Gachon University, Incheon, Korea; 5 Department of Internal Medicine, Gachon University Gil Medical Center, Incheon, Korea; 6 Department of Microbiology, College of Medicine, Gachon University, Incheon, Korea; 7 Department of Anatomy and Neurobiology, College of Medicine, Kyung Hee University, Seoul, Korea; 8 Department of Life Sciences, Gachon University, Seongnam, Gyeonggi-do Korea; 9 Department of Surgery, Gachon University Gil Medical Center, Incheon, Korea

**Keywords:** Cancer genomics, Cancer genomics

## Abstract

Although colorectal cancer is one of the most lethal cancer types in the world, its metastasis to the ovary is rare, compared to metastasis to other organs. Consequently, the genomic basis for colon-to-ovary metastasis remains unstudied, due to limited available patients, and thus there have been no attempts to construct individual-specific networks. Due to its rarity, the small sample size makes common mutations difficult to find. To overcome this problem, we herein attempted to apply a biological connectivity map called a sample-specific network (SSN), to reveal common biological functions in three samples. Our three samples were compared to a clinical dataset contained in The Cancer Genome Atlas (TCGA) Colorectal Adenocarcinoma (COAD), showing different mutational spectra, compared to matched samples based on age, gender, microsatellite instability (MSI) status, and tumor, node, metastasis (TNM) stage. The SSNs for the three samples revealed significant correlations of the mutation statuses of several apoptosis genes, in contrast to the TCGA-matched samples. Further analysis of a targeted-gene panel sequencing dataset for colon-to-ovary metastasis of primary tumor samples also confirmed significant correlations of the mutational statuses among apoptosis genes. In summary, using SSN, we successfully identified a common function (apoptosis) among our three patients having colon-to-ovary metastasis, despite no common mutations in the three patients. Such computational analyses could facilitate productive study of rare cancers and other diseases.

## Introduction

Colorectal cancer (CRC) is the third-most common cancer in the world, with an estimated 43.7 new cases, per 100,000 men and women, per year, and the fourth-leading lethal cancer, with 881,000 deaths in 2018^1^. During the past decade, clinicians have witnessed remarkable progress in the treatment of metastatic colon cancer, especially upon understanding its molecular basis, leading to effective combinations of chemotherapy regimens of oxaliplatin, irinotecan, cetuximab, and bevacizumab^2^. These novel agents have yielded both higher tumor response rates and prolonged overall survival.

Despite this substantial improvement in overall survival, however, CRC patients with rare colon-to-ovary metastases still have poorer prognoses and chemotherapy responses, compared to other metastatic sites^3–6^. It was also reported that, remarkably, the non-ovarian metastases tumor response rate to 5-FU-based chemotherapy was 40%, but only 5% for ovarian metastases^7^. Although Lee *et al*.^6^ reported that ovarian metastatectomy improved survival^6^, better understanding of the underlying mechanism(s) for resistance to nonsurgical therapy is urgently needed.

The frequency of ovarian metastasis from a primary CRC is low, at approximately 3.4% of women diagnosed with a malignant colorectal cancer^8^, compared to higher occurring metastases to the liver and the lung^9^. Due to this rarity, limited sample sizes (and thus low statistical power) represent a major hurdle for genomic analyses of CRC-ovarian metastasis. Therefore, it is important to apply a proper strategy to analyze mutational phenomena, using a systematic view, not only by comparing mutational occurrences.

In light of these challenges we performed a systematic study to improve the genetic characterization of ovarian metastases. We employed a novel bioinformatics approach to investigate genetic characteristics of CRC-ovarian metastatic tumors, by applying a sample-specific network (SSN) method to analyze unbiased, whole-exome sequencing (WES). A SSN allows creation of an individual-specific molecular network, via genomic profiling of a single sample^10^. We then compared the genetic landscape between primary colon tumors and their colon-to-ovary metastases, to attempt to reveal potential biomarkers for this rare condition.

## Results

### Demographic and clinical manifestations

Simultaneous ovarian metastases from three Korean female CRC patients, aged 52.33 ± 1.52 years old (range of 51 to 54 years old), were collected. All three patients possessed wild type *KRAS* and microsatellite stability (MSS), and one patient showed *HER2* amplification, as determined by pathologic examination. These and other clinical details are described in Table 1 and the Materials and Methods.

**Table 1:** Clinico-pathological data of the three patients.

Patient #	Gender	Age	Survival status (survival time/months)	TNM stage	KRAS	EGFR status	Microsatellite instability	Primary tumor size(cm)	Primary tumor treatment	Ovary metastasis size(cm)	Metastasis treatment
5	F	54	Alive (11)	IV	WILD	+	Stable	5	Anterior resection	4	FOLFIRI/FOLFOX/Cetuximab/Radiation
8	F	52	Alive (48)	IV	WILD	+	Stable	3.5	Low anterior resection	10	FOLFIRI/FOLFOX/Cetuximab/Bevacizumab/
9	F	51	Alive (10)	IV	WILD	+	Stable	3	Anterior resection	1	FOLFIRI/FOLFOX/Bevacizumab/lapatinib

### Pathway mutations in CRC tumors

**Figure 1:**
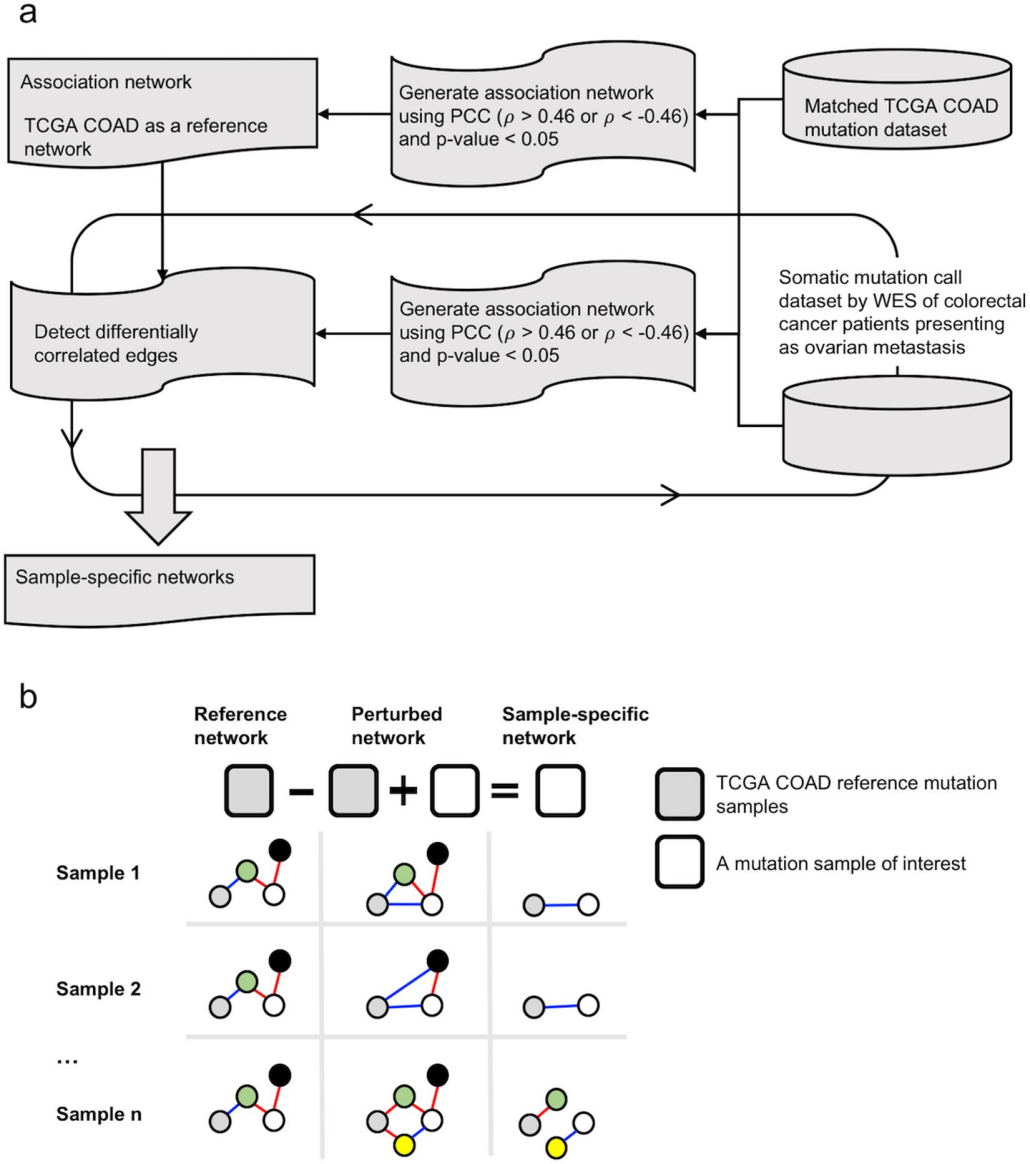
Framework of building sample-specific networks. (**a**) We built an association network (a “reference network”), using mutation information from TCGA-matched samples. Each of our samples was compared to the reference network, to build another association network of the sample, i.e., a “perturbed network”. We then used a Pearson correlation-based statistical test to identify differentially correlated edges between the “reference network” and the “perturbed network”. We applied this process to all patients’ somatic mutation sample datasets. (**b**) A conceptual illustration of the building of the sample-specific network. *ρ* is a correlation coefficient.

We first examined the mutational profiles of our patients, to subsequently built SSNs, using a method to determine how mutational co-occurrences of each patient’s ovarian CRC metastasis differed in samples matched for age, gender, and tumor stage^10^_._ Such data, of five samples selected (henceforth, “TCGA-matched samples”), was retrieved from The Cancer Genome Atlas (TCGA), allowing us to build tumor mutational co-occurrence networks. We first built such networks from mutation data of the TCGA-matched samples, using the Pearson correlation coefficient as an association index. This network, henceforth referred to as a “reference network”, contained molecular-level mutational co-occurrences among genes^10^. Then, we added each patient’s primary tumor mutational profile to the mutation dataset of the TCGA-matched samples, generating a “perturbed network”, i.e., a network derived from a “reference network,” by adding a sample of interest^10^. Then, we used a statistical test to identify differential “edges” between the reference network and the perturbed network^10^. Here, a “node” represents a gene, while an edge represents a relationship, between two nodes, in the perturbed network. The value of an edge represents a quantitative strength of how two nodes are related. The entire framework of our method is illustrated in Fig. 1.

**Figure 2:**
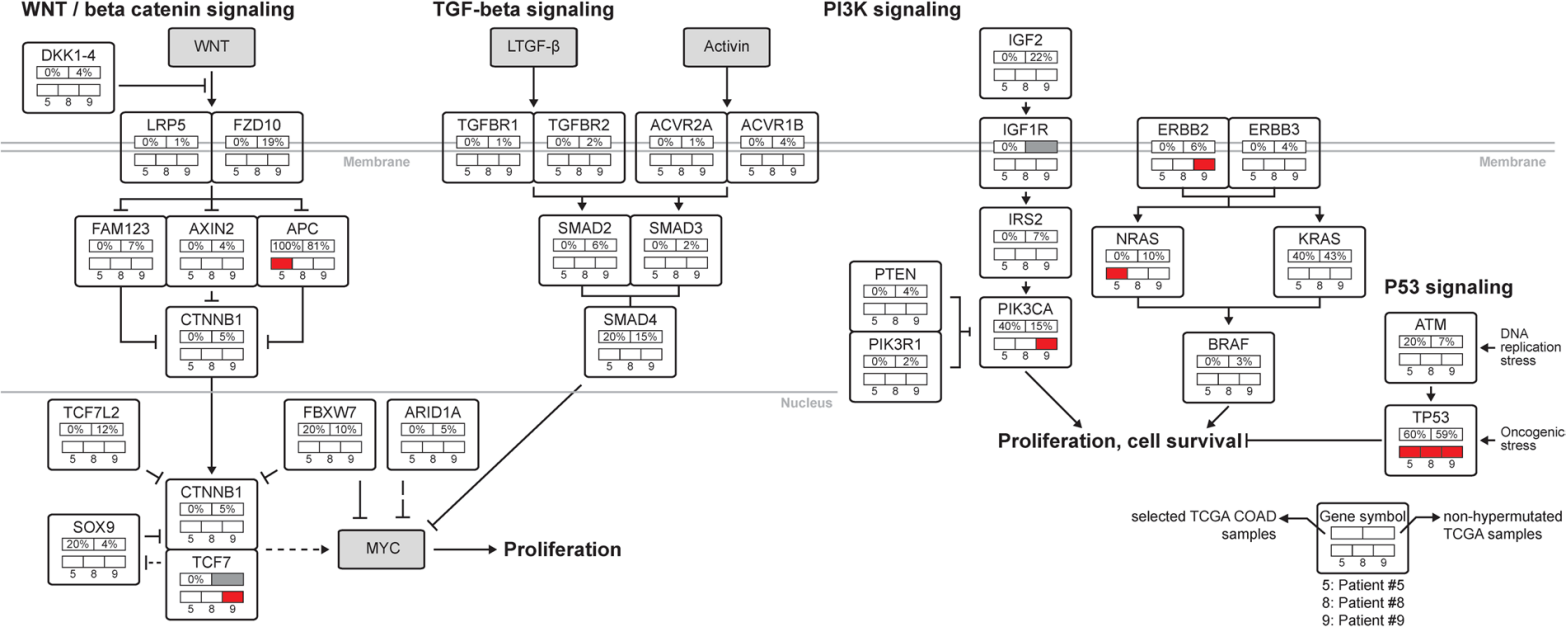
Diversity and frequency of genetic changes in our patients. *APC* was reported highly altered in the TCGA COAD report^11^ and we sought similar mutation ratios in the TCGA-matched samples. However, in our patients, only patient #5 had an APC mutation. All our patients had mutated *TP53*, which was mutated in only 60% of the TCGA-matched samples. Each gene has two box rows. In the upper box row, the left cell indicates a percentage of mutation observed from the TCGA-matched samples, and the right cell represents a percentage of alterations reported in TCGA COAD. A gray-filled box means a missing value. In the lower box row, three cells represent mutation statuses of patients #5, #8, and #9, from left to right (red cells = mutation; white cells = wild type).

We identified mutational profiles of members of the WNT beta-catenin signaling pathway, including MAPK, PI3K, TGF-*β*, and p53 pathways, when comparing our three patients to the TCGA-matched samples (Fig. 2). In Fig. 2, along with mutational occurrences of our three patients, we juxtaposed frequencies of genetic alterations of a non-hypermutated group (defined as a mutation rate of <8.24 per 10^6^ base pairs)^11^ from the TCGA COlorectal ADenocarcinoma (COAD) report in 2012^11^. While only one of our three patient samples had a somatic *APC* mutation, the TCGA-matched samples all showed mutated *APC* (81% of the non-hypermutated group had *APC* alterations) in the WNT/beta-catenin signaling pathway (Fig. 2). Although all three of our patients had *TP53* mutations, only 60% of the TCGA-matched samples had these (59% of non-hypermutated group showed *TP53* alteration) (Fig. 2). For *PIK3CA* (a member of the PI3K signaling pathway), one of the three, and 40% of the TCGA-matched samples, had mutations, although only 15% were found altered in the non-hypermutated group (Fig. 2). The DNA double-strand break repair enzyme gene, *ATM*, was not mutated among our patients, although 20% of the TCGA-matched samples did possess *ATM* mutations, as did 7% of the non-hypermutated samples^11^ (Fig. 2).

### Differential correlation changes, of mutation statuses, among genes, in our three patients

**Figure 3:**
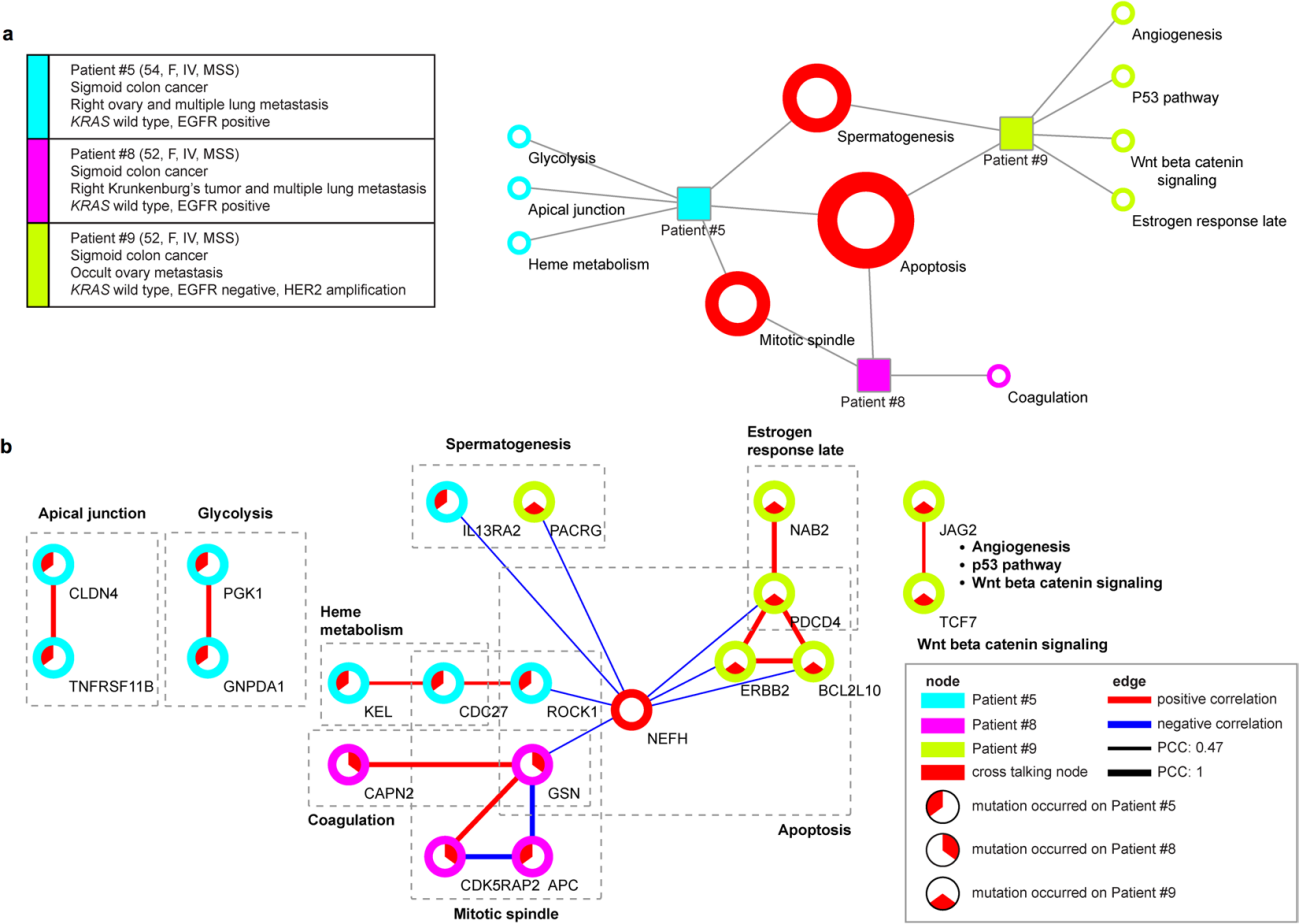
Inspection of sample-specific networks across our three colorectal cancer patients presenting ovarian metastases, compared to the TCGA-matched samples. (**a**) Network view showing the hallmark gene sets, as their correlation coefficients changed significantly among our colon-to-ovary cancer patients, compared to the TCGA-matched samples. (**b**) Gene-gene interaction diagram showing the mutational spectra of each patient. Colored circles indicate patients and crosstalk nodes. Colored edges indicate positive (red) and negative (blue) correlations, and their respective thicknesses indicate amounts of correlation coefficients. PCC: Pearson correlation coefficient.

The apoptosis signaling pathway was a common functional context among our three patient ovarian primary tumors, when we constructed three perturbed networks, and then merged these into one network (Fig. 3a). These assessments revealed that apoptosis-related gene correlation coefficients of mutational statuses (between two neighboring genes), were changed significantly in three of our patients, #5, #8, and #9. In all our patients, the neurofilament peptide gene *NEFH* (not mutated in the network of the TCGA-matched samples) correlated with genes belonging to the apoptosis process (Fig. 3b). For patient #5, *IL13RA2* and *ROCK1* (a Rho kinase) negatively correlated with *NEFH*, in terms of their mutation statuses. *ROCK1* was also a crosstalk gene between the apoptosis and mitotic spindle pathways. For patient #8, *GSN* (gelsolin), which facilitates crosstalk between the apoptosis and coagulation pathways, negatively correlated with *NEFH* (Fig. 3b). For patient #9, *ERBB2*, *BCL2L10*, *PDCD4*, and *PACRG*, all showed negative correlations with *NEFH* (Fig. 3b). *ERBB2*, an oncogene, and *BCL2L10*, belonging to the apoptosis pathway, positively correlated with each other and *PDCD4*, of the estrogen response late pathway (Fig. 3b). Since patient #9 had *HER2* amplification, its crosstalk genes (*PDCD4, BCL2L10, NEFH*) should be later examined for correlation with *ERBB2*, and possible clinical associations. Changes in gene correlation are described in Table 2.

**Table 2:** Correlation coefficient changes in sample-specific networks.

Interaction (Genes A-B)	Hallmark gene sets	W_AB_^a^	ΔW_AB_^b^	p-value of interaction
JAG2-TCF7	WNT BETA CATENIN SIGNALING	0	0.65	0.001
PACRG-NEFH	SPERMATOGENESIS	0	−0.47	0.018
BCL2L10-PDCD4	APOPTOSIS	0	1.00	5.73E-07
BCL2L10-ERBB2	APOPTOSIS	0	1.00	5.73E-07
BCL2L10-NEFH	APOPTOSIS	0	−0.47	0.018
PDCD4-NAB2	ESTROGEN RESPONSE LATE	0	1.00	5.73E-07
PDCD4-ERBB2	APOPTOSIS	0	1.00	5.73E-07
PDCD4-NEFH	APOPTOSIS	0	−0.47	0.018
ERBB2-NEFH	APOPTOSIS	0	−0.47	0.018
CDK5RAP2-GSN	MITOTIC SPINDLE	0	1.00	5.73E-07
APC-GSN	MITOTIC SPINDLE	0	−1.00	5.73E-07
APC-CDK5RAP2	MITOTIC SPINDLE	0	−1.00	5.73E-07
GSN-CAPN2	COAGULATION	0	1.00	5.73E-07
GSN-NEFH	APOPTOSIS	0	−0.47	0.018
CLDN4-TNFRSF11B	APICAL JUNCTION	0	1.00	5.73E-07
PGK1-GNPDA1	GLYCOLYSIS	0	1.00	5.73E-07
IL13RA2-NEFH	SPERMATOGENESIS	0	−0.47	0.018
KEL-CDC27	HEME METABOLISM	0	0.65	0.001
CDC27-ROCK1	MITOTIC SPINDLE	0	0.65	0.001
ROCK1-NEFH	APOPTOSIS	0	−0.47	0.018

W_AB_ and ΔW_AB_ are described in detail in the Methods section. We also added the relevant hallmark gene sets for an interaction of two genes. The p-value was obtained from the Z-value that showed a ΔW_AB_ of an edge was significant statistically.

^a^Pearson correlation coefficient between two gene pair in a “reference network”.

^b^Degree of Pearson correlation coefficient change between two gene pair in a “perturbed network”.

We next assessed whether the functional contexts (apoptosis, mitotic spindle, and spermatogenesis), in our dataset, were likewise present in an independent dataset. To that end, we analyzed a targeted panel sequencing dataset of 26 colorectal ovarian metastases^12^, identifying significant correlation changes in 23 of the 26 samples, compared to the TCGA-matched samples. Those three contexts were revealed again (HALLMARK_APOPTOSIS in ten patients, HALLMARK_MITOTIC_SPINDLE in four patients, and HALLMARK_SPERMATOGENESIS in one patient) in the targeted panel sequencing dataset (Supplementary Fig. S1a). Moreover, there were significant correlation changes of mutational statuses in apoptosis genes, in ten of the 26 samples (HALLMARK_APOPTOSIS in Supplementary Fig. S1a), with apoptosis nodes also correlating with *NEFH*, forming six different network topological patterns (Supplementary Fig. S1b). *NEFH* also negatively correlated with *ERBB2*, in five samples (patterns 1 and 2 in Supplementary Fig. S1b). In three samples, *NEFH* negatively correlated with the metastasis-related gene, *MMP2* (patterns 3 and 4 in Supplementary Fig. S1b), and in two samples, *NEFH* negatively correlated with *SMAD7* (patterns 5 and 6 in Supplementary Fig. S1b). As a result, we revealed that our dataset, and the independent dataset, shared common functional contexts, with *NEFH* implicated in both datasets.

### Comparison of mutations of primary colon and metastasized ovarian tumors

In our three patients, both primary and metastatic ovarian tumor tissues were compared, showing that all patients shared *TP53* mutations in both their primary and metastasized tumors (Supplementary Table S1). Consequently, we built two mutational co-occurrence networks for the primary CRC and metastasized ovarian tumors, to compare their topological configurations. We hence observed a network structural similarity between the two networks, based on single nucleotide variations (SNVs, Supplementary Table S1). Overall, significant genes, and their correlations, were preserved in the two mutational co-occurrence networks, although there were changes of neighboring partners or correlational statuses (Supplementary Fig. S2). For example, the gene pairs *APC*-*CDC27*, and *CDC27*-*ROCK1* were positively connected in the CRC network, while these became negatively connected in the metastatic network.

## Discussion

In this study, we studied the mutational landscape of three patient samples of rare ovarian colorectal (CRC) metastases, as compared to their primary CRC tumors. The mutational co-occurrences of our three samples showed different mutational co-occurrences, both in age-/tumor stage-matched samples in the TCGA, and in a non-hypermutated group, a TCGA COlorectal ADenocarcinoma (COAD) report from 2012^11^. All three patients had *TP53* mutations, which were present in only 60% of the TCGA-matched and non-hypermutated patient samples. For the CRC-causing gene *APC*, in the WNT/beta-catenin signaling pathway, all the TCGA-matched, and 81% of non-hypermutated, samples, had mutations, but only one of our three ovarian metastasis-suffering patients had somatic *APC* mutations.

We also detected significant correlation changes of mutations statuses of genes, belonging to apoptosis, in all three SSNs derived from our patients’ samples, when we applied a statistical method to identify significant differential correlation changes. Another oft-present gene, *NEFH*, encodes a heavy polypeptide that is assembled into neurofilaments^13^, the main cytoskeletal components of a mature neuron^14^. Aberrations in neurofilaments have been implicated in numerous neurological diseases^15–17^, although *NEFH* has been little studied in cancer^18,19^, where it was observed in human autonomic nerve tumors and central neurocytomas^20–22^. Hypermethylated *NEFH* may also confer poor prognosis in breast cancer^23^, and its dysregulation has been observed in lung, prostate, and renal cancers^24–26^. Our three patients showed negative correlations in *NEFH* with six genes, *PDCD4*, *ERBB2*, *BCL2L10*, *ROCK1*, *IL3RA2*, and *GSN*, in the hallmark apoptosis gene set (Fig. 3b). *NEFH*, however, had no relation with these six genes in the reference network.

In the independent targeted-gene panel sequencing analysis (Supplementary Fig. S1b), *NEFH* mutation statuses directly associated with the oncogene *ERBB2* mutation statuses, which was consistent with our dataset. *NEFH* mutation statuses also associated with *CTNNB1* mutation statuses, a member of the WNT oncogenic signaling cascade, through *ERBB2* and *SMAD7* (Supplementary Fig. 1b; patterns 2 and 6). *NEFH* also associated with *APC* (another WNT pathway member, upstream of *CTNNB1*), through *GSN*, in our dataset analysis (Fig. 3b), Thus, these findings might implicate *NEFH* in WNT signaling in cancer^19,27,28^, even as *NEFH*’s role in cancer is yet to be discovered. Recent studies^19,27,28^ have reported NEFH protein to be linked to Akt/beta-catenin signaling^27,28^, as well as a tumor suppressor role^19^. Due to mutation status changes of the WNT genes (*APC* and *CTNNB1*)*, NEFH* might lose its tumor suppressor role, leading to apoptosis dysregulation.

Metastasis is a complex, multi-step process that leads to the accumulation of genomic alterations in primary tumor cells, during malignancy progression, from the primary tumor, to localized invasion, to metastasis^29,30^. Genomic changes, and characteristics of metastatic-stage tumors, are complex and only partially understood. Chemotherapy-naïve primary tumors, and their paired metastases, frequently share many altered genes at both sites, especially in liver metastasis^31^. However, emerging evidence suggests that treatment of colon cancer patients with anti-EGFR agents increases intra-tumor heterogeneity, leading to the emergence of clones with different genetic alterations^32^. To assess this, we used chemo-naïve synchronous colon cancer-to-ovary metastasis samples. Also, considering well-conserved network configurations between co-occurrence networks of the primary CRC and the metastasized ovarian networks (Supplementary Fig. S2), our systematic approach discovered that the paired primary CRC and metastasized ovarian tumors share many altered genes and less heterogeneity.

Colon cancer with ovarian metastasis is less responsive to chemotherapy, compared to non-ovarian metastases^5,6^. Likewise, we observed no tumor response of ovarian metastases^5^, whereas the tumor regression rate for non-ovarian metastatic lesions was over 65%, in a series of twenty-two patients with colon cancer ovarian metastases^5^. Based on these reports, the ovary has been regarded as a “sanctuary” organ for resistance to systemic chemotherapy. Patient #5 has now survived over four years. Thus, the underlying mechanism of chemoresistance needs to be determined, including several tumor features such as volume, cystic properties, and mucin-richness, as these may contribute to poor sensitivity to chemotherapy^6^.

The observation of HER2 (*ERBB2*) amplification in one tumor can have major impact on treatment strategies in one patient, and for the patient #9, after 1 cycle of lapatinib, the patient was switched to palliative care, due to disease progression. Although the majority of metastatic CRC patients had HER2 (*ERBB2*) amplification^33^, our study only considered mutations, not amplifications, and moreover HER2 amplification in one of our patients might be insufficient to explain biology in ovarian metastasis.

In summary, although ovarian metastasis from the colon is rare^9^, precluding (due to insufficient sample sizes) most statistical methods that merely identify common mutations, we successfully applied network biology to this disease. The result of that analysis was that these patients all shared a common biological function, i.e., a perturbed network involving the process of apoptosis. We strongly assert that such approaches will allow analyses of similarly uncommon maladies, facilitating increased understanding of rare disease pathogenesis.

## Materials and Methods

### Patient samples

Three patients who were pathologically diagnosed with colon cancer, with ovary metastases, were included in this study. All subjects were recruited from Gachon Gil Medical Center (Gachon University, South Korea), and the study was approved by the Institutional Review Board of Gachon University Gil Medical Center (IRB#GIRBA2216). Written informed consents were obtained from the patients, in accordance with institutional guidelines. All methods were performed in accordance with the relevant guidelines and regulation. These patients were chemo-naive at the time of primary colon surgery, and were diagnosed with ovary metastasis during the first operation.

#### Patient #5

A 54-year old woman, presenting with ongoing lower abdominal pain, had obstructing distal sigmoid colon cancer, with synchronous right ovary metastasis and multiple lung metastases. The patient’s initial cancer embryonic antigen (CEA) level was 85.31 ng/mL, and she underwent palliative anterior resection, with bilateral oophorectomy, when the primary tumor and ovary metastases were collected and freshly frozen. The pathologic features were as follows: wild type *KRAS*, T4a lesion, lymphovasular invasion, perineural invasion, and 9 of 20 lymph node metastases with negative resection margins. The patient underwent palliative chemotherapy with FOLFIRI (leucovorin calcium, 5-fluorouracil, and irinotecan) and cetuximab. Currently, the patient is in therapeutic remission for stable lung metastasis.

#### Patient #8

A 52-year-old female presented with a 3.5-cm sigmoid colon cancer with 10-cm sized right Krukenburg’s tumor, and multiple lung metastases. The patient underwent palliative low anterior resection, with total hysterectomy and bilateral oophrectomy (tissue procurement of the primary tumor and ovarian metastasis). The pathologic features were as follows: adenocarcinoma, wild type *KRAS*, moderate differentiation, direct invasion of the uterine myometrium (T4b), lymphovascular invasion, perineural invasion, and 20 positively metastatic of 60 lymph nodes dissected. Thirteen months later, the patient developed a small recurrent tumor in her lumbar spine, and underwent radiation therapy (3600 cGy from 300 cGy x 12 fractions), and 12 cycles of oxaliplatin-based chemotherapy (FOLFOX6) thereafter. Shortly after chemotherapy, lung metastasis progressed. The patient still has slowly progressive, multiple lung metastases refractory to cetuximab and bevacizumab. The patient is still alive, four years after the initial surgery.

#### Patient #9

A 51-year old female presented with obstructing sigmoid colon cancer, with occult ovarian metastasis, and underwent anterior resection, with intraoperative bowel lavage with simultaneous bilateral oophorectomy. Pathologic examination revealed metastases in both ovaries, with wild-type *KRAS* and HER2 amplification. After 12 cycles of oxaliplatin-based chemotherapy, the regimen was switched to bevacizumab plus irinotecan-based chemotherapy. Shortly after 1 cycle of lapatinib, the patient was switched to palliative care, due to disease progression.

**Figure 4:**
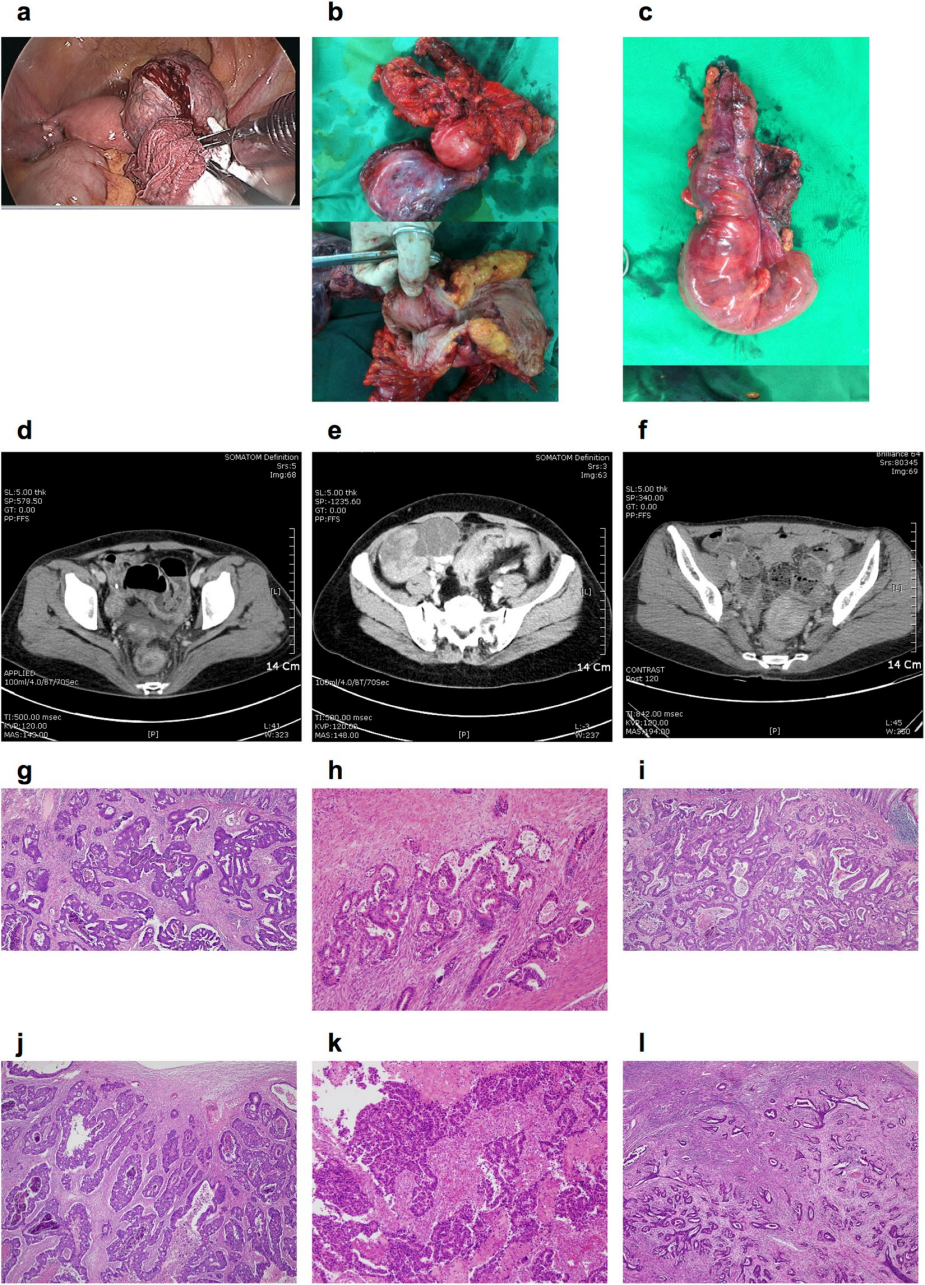
Macroscopic examinations of the excised tumor specimens. (**a**) Patient #5 underwent palliative anterior resection, with oophorectomy, when freshly frozen primary tumor and ovary metastasis tissue was collected. (**b**) Patient, #8 underwent palliative low anterior resection, with total hysterectomy and bilateral oophorectomy. (**c**) Patient #9 underwent anterior resection, with intraoperative bowel lavage and simultaneous bilateral oophorectomy. Abdominal enhanced computed tomography of patients (**d**) #5, (**e**) #8, and (**f**) #9. Histological analysis of the excised colon tumor specimens of (**g**) patients #5, (**h**) #8, and (**i**) #9. Histological analysis of the excised ovary tumor specimens of patients (j) #5, (**k**) #8, and (**l**) #9.

Clinicopathological features of the three patients are listed in Table 1, and surgical specimens from the three above-described patients, their corresponding abdominopelvic CT scans, and histologies, are shown in Fig. 4.

### Whole-exome sequencing

We used exome sequencing, at 200X coverage, to profile somatic mutations in primary colon cancers, and synchronous ovary metastases.

#### DNA preparation

Genomic DNA was extracted from tumors and normal tissues, using a QIAamp DNA mini kit (Qiagen Inc, Valencia, CA, USA), according to the manufacturer’s protocol. DNA quality and quantity were assessed using a Nanodrop spectrometer (Nanodrop Technologies, Wilmington, DE, USA) and a Qubit fluorometer (Life Technologies, Invitrogen, Carlsbad, CA, USA).

#### Experimental DNA sequencing

Whole-exome sequencing was performed using SureSelect Human All Exon V5 51 Mb (Agilent, Santa Clara, CA, USA), following the manufacturer’s standard protocol. A paired-end DNA sequencing library was prepared through genomic DNA shearing, use a Covaris™ (Woburn, MA, USA) sonicator, followed by peak detection, end repair, poly A-tailing, paired-end adaptor ligation, and amplification. After hybridization of the library with bait sequences for 24 hours, the captured library was purified and amplified with an index barcode tag, and the library quality and quantity were determined. Sequencing of the exome library was carried out using the 100-bp paired-end mode of the HiSeq SBS kit (Illumina, San Diego, CA).

#### Whole-exome sequencing processing and alignment

Sequence reads in FASTQ format were mapped to the human assembly UCSC hg19, using the Burrows-Wheeler Aligner (BWA, v0.7.7)^34^ with ‘mem’ and seed value parameters ‘-k 45’, to create SAM files with correct mate pair information, with a read group tag that included the sample name. We then used Picard (v1.92) to convert SAM files to compressed BAM files, and sorted the BAM files by chromosome coordinates. The Genome Analysis Toolkit (v2.3.9Lite)^35^ was used to locally realign the BAM files at intervals that may have insertion/deletion (indel) alignment errors. Somatic mutations and indels were “called” with Mutect^36^ and GATK Somatic Indel Detector (Broad Institute), respectively. Single nucleotide variants and indels were annotated using snpEff (v4.1a)^37^ to classify variants as synonymous, non-synonymous, missense, or frameshift point mutations and frameshift indels. We provide all variants detected in primary CRC tumors (single nucleotide variants in Supplementary Table S3 and indels in Supplementary Table S4) and metastasized ovarian tumors (single nucleotide variants in Supplementary Table S5 and indels in Supplementary Table S6).

### TCGA sample selection for reference network construction

#### Dataset from The Cancer Atlas Genome (TCGA)

We downloaded a TCGA primary colon cancer^11^ mutation dataset from the UCSC Cancer Genomics Browser^38^ (last accessed on 02-21-2018), namely, the version of TCGA COAD mutation database 2015-02-24. From this mutation dataset, we selected five samples under the following conditions; female, age at initial pathologic diagnosis between 46 and 59 years-old, MSI, and tumor stage IV (IVA and IVB included). The age range of the five samples was approximately close to that of our three ovarian metastasis-bearing patients. These samples were referred to as “TCGA-matched samples” throughout the manuscript. Supplementary Table S2 lists the clinicomolecular characteristics of the TCGA-matched samples selected for the reference network construction.

#### Mutational co-occurrence network

We calculated a correlation coefficient between mutation statuses of patients for each gene pair (genes A and B) to build mutational co-occurrence profiles, using Pearson’s correlation coefficient (PCC).

$$Correlation\,coefficien{t}_{AB}=\frac{cov(A,B)}{{\sigma }_{A}{\sigma }_{B}}$$

where *cov* is a covariance, and *σ* is a standard deviation. We then constructed a reference network of mutational co-occurrences in the TCGA-matched samples, with Pearson correlation coefficient (PCC) cutoff criteria; |PCC| > 0.46 and p-value < 0.05, according to Green *et al*.^39^. Correlation states refer to positive, negative, and noncorrelation. A PCC > 0.46 was considered a positive correlation, and a PCC < −0.46 a negative correlation. A PCC between −0.46 and 0.46 was considered noncorrelation. Genes of interest in calculating PCCs focused on the hallmark gene sets provided by MIT MSigDB^40^.

#### Detecting significant correlation changes between two genes

For SSN construction for our individual samples, Lie *et al*.^10^, showed that the statistical significance of an edge, (correlation of mutation statuses) between two genes (equivalently, nodes; say, nodes A and B), could be verified by the Z-test, and the Z-value could be defined as below:

$$Z=\frac{\Delta {W}_{AB}}{\frac{1-{{W}_{AB}}^{2}}{n-1}}$$

where *W*_*AB*_ is a PCC value (for the TCGA-matched samples) of an edge between nodes A and B; X_AB_ is a PCC value between nodes A and B, when the TCGA-matched samples plus our individual samples were combined; Δ*W*_*AB*_ is X_AB_ minus W_AB_.; and *n* is a number of samples used to build the reference network. Edges in associated networks were selected from those correlation values that changed significantly in a statistical test. We only considered gene pairs with one type of association change (Δ*W*_*AB*_), gain-of-correlation, which indicates noncorrelation of W_AB_ changed to either positive or negative correlation, of X_AB_. We did not consider the case where correlation direction (e.g., positive, negative) of W_AB_ was not changed in X_AB_, or was changed to noncorrelation.

### Targeted gene sequencing dataset of colorectal cancer patients metastasized to ovaries

To evaluate the consistency of the functional contexts of our dataset, we evaluated another colorectal cancer dataset metastasized to ovaries^12^. This dataset was a targeted gene sequencing dataset using its own custom-made 115-gene panel. The entire gene list, for the panel, and list of mutations in primary COAD tumors and metastases to ovaries, are available in the supplementary materials of their report^12^. From that data set, we used the mutation profiles of the primary COAD tumors. Because this dataset was from the targeted gene panel, *NEFH* was not listed in the panel. Thus, we queried the mutational occurrence ratio of *NEFH*, in TCGA COAD to cBioPortal^41^, and the occurrence ratio of *NEFH* aberrations was 1.5% (among 594 samples, 1 amplification, 1 deep deletion, 1 truncating mutation, and 6 missense mutations, last accessed DEC 31, 2018). However, the majority of TCGA COAD primary tumor tissue samples did not have mutations, and we assumed that there was no mutation on *NEFH* in this targeted sequencing dataset.

## Supplementary information

Supplementary material

## Data Availability

NCBI BioProject accession: PRJNA489923.
