# Supplementary material for: Systematic mutation analysis in rare colorectal cancer presenting ovarian metastases
Source: Sci Rep. 2019 Nov 18;9:16990. doi: 10.1038/s41598-019-53182-6 (PMC6861287; doi:10.1038/s41598-019-53182-6)
Supplement: Supplementary file 1 — Supplementary material [file 41598_2019_53182_MOESM1_ESM.docx]

**Systematic mutation analysis in rare colorectal cancer presenting ovarian metastases**

Sungjin Park^†^, Hee Kyung Ahn^†^, Dae Ho Lee, YunJae Jung, Joo-Won Jeong, Seungyoon Nam^*^, Won-Suk Lee^*^

**Supplementary Information**

**Contents**
Supplementary Tables S1 through S6

Supplementary Figure S1 and S2

Supplementary Table S1. Comparing mutational spectrum of our three primary colon and their metastasized ovary tumors.

|  | **Colon** | | | **Ovary** | | |
| --- | --- | --- | --- | --- | --- | --- |
| **Gene symbol** | **Patient #5** | **Patient #8** | **Patient #9** | **Patient #5** | **Patient #8** | **Patient #9** |
| DNAH5 | 0 | 1 | 0 | 0 | 1 | 0 |
| TTN | 1 | 0 | 0 | 1 | 0 | 0 |
| BAI2 | 0 | 0 | 0 | 1 | 0 | 0 |
| CCDC73 | 0 | 0 | 0 | 0 | 1 | 0 |
| CNP | 0 | 0 | 0 | 0 | 1 | 0 |
| CPA4 | 0 | 0 | 0 | 0 | 1 | 0 |
| CTBP2 | 0 | 0 | 0 | 1 | 0 | 0 |
| DDX60L | 0 | 0 | 0 | 0 | 1 | 0 |
| DENND1C | 0 | 0 | 0 | 0 | 0 | 1 |
| DHX38 | 0 | 0 | 0 | 0 | 1 | 0 |
| DSP | 0 | 0 | 0 | 0 | 1 | 0 |
| FAM194A | 0 | 0 | 0 | 0 | 0 | 1 |
| FBN1 | 0 | 0 | 0 | 0 | 1 | 0 |
| FEZF2 | 0 | 0 | 0 | 0 | 1 | 0 |
| FLNA | 0 | 0 | 0 | 1 | 0 | 0 |
| FREM2 | 0 | 0 | 0 | 0 | 1 | 0 |
| FRY | 0 | 0 | 0 | 0 | 1 | 0 |
| GPR101 | 0 | 0 | 0 | 0 | 1 | 0 |
| GUCY2C | 0 | 0 | 0 | 0 | 1 | 0 |
| HLA-C.2 | 0 | 0 | 0 | 1 | 0 | 0 |
| LYZL4 | 0 | 0 | 0 | 0 | 0 | 1 |
| MAP2 | 0 | 0 | 0 | 1 | 0 | 0 |
| MTMR1 | 0 | 0 | 0 | 0 | 1 | 0 |
| MYF5 | 0 | 0 | 0 | 0 | 0 | 1 |
| NIPBL | 0 | 0 | 0 | 0 | 1 | 0 |
| NR2F1 | 0 | 0 | 0 | 1 | 0 | 0 |
| PAXBP1 | 0 | 0 | 0 | 0 | 0 | 1 |
| PDHA2 | 0 | 0 | 0 | 1 | 0 | 0 |
| RASA4 | 0 | 0 | 0 | 0 | 0 | 1 |
| RBMXL1 | 0 | 0 | 0 | 0 | 1 | 0 |
| RGL3 | 0 | 0 | 0 | 1 | 0 | 0 |
| RNF180 | 0 | 0 | 0 | 0 | 1 | 0 |
| ROBO1 | 0 | 0 | 0 | 1 | 0 | 0 |
| RYR1 | 0 | 0 | 0 | 0 | 0 | 1 |
| SERPINB3 | 0 | 0 | 0 | 1 | 0 | 0 |
| SIGLEC11 | 0 | 0 | 0 | 1 | 0 | 0 |
| SLC17A4 | 0 | 0 | 0 | 0 | 1 | 0 |
| SLC22A20 | 0 | 0 | 0 | 0 | 1 | 0 |
| SLC2A12 | 0 | 0 | 0 | 0 | 1 | 0 |
| SLC35F1 | 0 | 0 | 0 | 0 | 0 | 1 |
| SYP | 0 | 0 | 0 | 1 | 0 | 0 |
| THOC7 | 0 | 0 | 0 | 0 | 1 | 0 |
| TJP3 | 0 | 0 | 0 | 0 | 1 | 0 |
| TLX2 | 0 | 0 | 0 | 0 | 0 | 1 |
| TMEM229A | 0 | 0 | 0 | 1 | 0 | 0 |
| TWIST2 | 0 | 0 | 0 | 0 | 1 | 0 |
| WIPI2 | 0 | 0 | 0 | 0 | 1 | 0 |
| ZIM3 | 0 | 0 | 0 | 0 | 0 | 1 |

0: wild typed gene

1: mutated gene

Supplementary Table S2. Clinico-molecular characteristics of the TCGA-matched patients as a "reference network".

| **TCGA sampleID** | **TCGA-A6-6142-01** | **TCGA-CK-4947-01** | **TCGA-CM-5868-01** | **TCGA-F4-6809-01** | **TCGA-G4-6297-01** |
| --- | --- | --- | --- | --- | --- |
| **Microsattlelite instability** | MSS | MSS | MSS | MSS | MSS |
| **Gender** | FEMALE | FEMALE | FEMALE | FEMALE | FEMALE |
| **Cohort** | TCGA Colon Cancer | TCGA Colon Cancer | TCGA Colon Cancer | TCGA Colon Cancer | TCGA Colon Cancer |
| **Anatomic neoplasm subdivision** | Sigmoid Colon | Sigmoid Colon | Sigmoid Colon | Sigmoid Colon | Cecum |
| **Race** | White | White | White | White | White |
| **Age at initial pathologic diagnosis** | 56 | 46 | 59 | 52 | 55 |
| **Height** | 162.5 |  | 171 | 150 |  |
| **Weight** | 88 |  | 99.8 | 88 | 64 |
| **Overall survival (days)** | 368 | 534 | 426 | 403 | 2506 |
| **OS status** | 0 | 0 | 0 | 1 | 0 |
| **Number of mutations (genes)** | 51 | 70 | 102 | 92 | 156 |
| **Pathologic stage** | Stage IVA | Stage IVA | Stage IVA | Stage IVA | Stage IV |
| **Pathologic M** | M1a | M0 | M1a | M1 | M1 |
| **Pathologic N** | N1a | N1 | N1a | N1 | N2 |
| **Pathologic T** | T3 | T4a | T4a | T3 | T3 |
| **Colon polyps present** | NO | NO | NO | NO | YES |
| **BRAF analysed** | NO | NO | YES | NO | NO |
| **BRAF mutation** |  |  | Normal |  |  |
| **KRAS analysed** | YES | NO | YES | NO | NO |
| **KRAS mutation** | NO |  | NO |  |  |

Supplementary Table S3 List of mutations (SNVs) in primary CRC tumours.

|  | | **Consequences** | | **Gene** | | **Transcript_id** | | **HGVS_C** | **HGVS_P** |
| --- | --- | --- | --- | --- | --- | --- | --- | --- | --- |
| #5 | | intron_variant | | LRRIQ3 | | NM_001105659.1 | | c.573+1878C>T | . |
| #5 | | 5_prime_UTR_variant | | TSPAN8 | | NM_004616.2 | | c.-105G>A | . |
| #5 | | missense_variant | | CDC27 | | NM_001114091.2 | | c.794G>A | p.Gly265Asp |
| #5 | | missense_variant | | RHPN2 | | NM_033103.4 | | c.217G>A | p.Val73Met |
| #5 | | intron_variant | | PVRL2 | | NM_001042724.1 | | c.1043-3608C>T | . |
| #5 | | missense_variant | | KCNH7 | | NM_033272.3 | | c.672A>T | p.Lys224Asn |
| #5 | | synonymous_variant | | AQP12A | | NM_198998.2 | | c.249T>C | p.Thr83Thr |
| #5 | | missense_variant | | XKR7 | | NM_001011718.1 | | c.961G>A | p.Ala321Thr |
| #5 | | intron_variant | | SLC12A5 | | NM_001134771.1 | | c.122-5538C>T | . |
| #5 | | missense_variant | | PIWIL3 | | NM_001008496.3 | | c.728G>A | p.Arg243His |
| #5 | | 3_prime_UTR_variant | | RRP7A | | NM_015703.4 | | c.*19A>G | . |
| #5 | | splice_region_variant&intron_variant | | FAM19A1 | | NM_001252216.1 | | c.259+8C>A | . |
| #5 | | missense_variant | | RGS12 | | NM_198229.2 | | c.3458G>T | p.Gly1153Val |
| #5 | | missense_variant&splice_region_variant | | CPZ | | NM_001014447.2 | | c.1606C>A | p.Pro536Thr |
| #5 | | non_coding_exon_variant | | NCF1B | | NR_003186.1 | | n.491C>G | . |
| #5 | | synonymous_variant | | POM121C | | NM_001099415.2 | | c.2346C>G | p.Ser782Ser |
| #5 | | stop_gained | | PEX1 | | NM_000466.2 | | c.547C>T | p.Arg183* |
| #5 | | intron_variant | | LAMTOR4 | | NM_001008395.2 | | c.203-153A>G | . |
| #5 | | missense_variant | | UNC5D | | NM_080872.2 | | c.901T>G | p.Cys301Gly |
| #5 | | missense_variant | | TNFRSF11B | | NM_002546.3 | | c.877G>A | p.Glu293Lys |
| #5 | | intergenic_region | | CNTNAP3B-LOC101927827 | | CNTNAP3B-LOC101927827 | | . | . |
| #5 | | missense_variant | | CXorf23 | | NM_198279.3 | | c.554C>G | p.Ser185Cys |
| #5 | | splice_region_variant&intron_variant | | ZNF182 | | NM_001178099.1 | | c.289+8G>A | . |
| #5 | | intron_variant | | AR | | NM_000044.3 | | c.1769-5150T>C | . |
| #5 | | intron_variant | | LRRIQ3 | | NM_001105659.1 | | c.573+1878C>T | . |
| #5 | | missense_variant | | NRAS | | NM_002524.4 | | c.182A>G | p.Gln61Arg |
| #5 | | intron_variant | | ADAMTSL4 | | NM_001288608.1 | | c.2832+12G>A | . |
| #5 | | intron_variant | | SPTA1 | | NM_003126.2 | | c.957+22G>A | . |
| #5 | | missense_variant | | CHIT1 | | NM_003465.2 | | c.802G>A | p.Gly268Arg |
| #5 | | missense_variant | | KLHDC8A | | NM_001271863.1 | | c.779G>A | p.Arg260Gln |
| #5 | | missense_variant | | TRAF3IP3 | | NM_025228.3 | | c.1127C>A | p.Ala376Asp |
| #5 | | intron_variant | | OBSCN | | NM_001271223.2 | | c.26150+39C>T | . |
| #5 | | intergenic_region | | LOC100129055-HSD17B7P2 | | LOC100129055-HSD17B7P2 | | . | . |
| #5 | | synonymous_variant | | RBP3 | | NM_002900.2 | | c.3546C>T | p.His1182His |
| #5 | | missense_variant | | HPSE2 | | NM_021828.4 | | c.881G>A | p.Arg294Gln |
| #5 | | intron_variant | | SORCS1 | | NM_001013031.2 | | c.960-68G>A | . |
| #5 | | intron_variant | | DNAJC24 | | NM_181706.4 | | c.320-18T>C | . |
| #5 | | 5_prime_UTR_variant,upstream_gene_variant | | YIF1A,TMEM151A | | NM_020470.2,NM_153266.3 | | c.-83C>G,c.-113G>C | .,. |
| #5 | | missense_variant | | SLCO2B1 | | NM_007256.4 | | c.1390G>T | p.Gly464Cys |
| #5 | | missense_variant | | C12orf5 | | NM_020375.2 | | c.198G>A | p.Met66Ile |
| #5 | | intron_variant | | NACA | | NM_001113203.2 | | c.1864+225C>T | . |
| #5 | | 5_prime_UTR_variant | | TSPAN8 | | NM_004616.2 | | c.-105G>A | . |
| #5 | | missense_variant | | CCDC64 | | NM_207311.2 | | c.865C>T | p.Arg289Trp |
| #5 | | missense_variant | | HTR2A | | NM_000621.4 | | c.712G>A | p.Gly238Ser |
| #5 | | synonymous_variant | | PCDH8 | | NM_002590.3 | | c.927C>T | p.Ala309Ala |
| #5 | | synonymous_variant | | IRG1 | | NM_001258406.1 | | c.642G>T | p.Gly214Gly |
| #5 | | intron_variant | | FRMD6 | | NM_001267046.1 | | c.1025-44G>A | . |
| #5 | | missense_variant | | MAGEL2 | | NM_019066.4 | | c.733C>A | p.Pro245Thr |
| #5 | | intron_variant | | GLDN | | NM_181789.2 | | c.817+36G>T | . |
| #5 | | splice_region_variant&intron_variant | | NARFL | | NM_022493.1 | | c.693+5C>T | . |
| #5 | | intron_variant | | CACNA1H | | NM_021098.2 | | c.4477-27G>A | . |
| #5 | | missense_variant | | GPR114 | | NM_153837.1 | | c.1334C>T | p.Ala445Val |
| #5 | | upstream_gene_variant,intron_variant | | LOC102724009,KIF1C | | NR_120665.1,NM_006612.5 | | n.-1G>A,c.1751-31C>T | .,. |
| #5 | | missense_variant | | TP53 | | NM_000546.5 | | c.413C>T | p.Ala138Val |
| #5 | | intron_variant | | DNAH2 | | NM_020877.2 | | c.1171-748T>C | . |
| #5 | | missense_variant | | CDC27 | | NM_001114091.2 | | c.794G>A | p.Gly265Asp |
| #5 | | intron_variant | | GRB2 | | NM_002086.4 | | c.78+23A>G | . |
| #5 | | intron_variant | | MYOM1 | | NM_003803.3 | | c.4378+17G>A | . |
| #5 | | missense_variant | | ROCK1 | | NM_005406.2 | | c.162C>A | p.Asn54Lys |
| #5 | | missense_variant | | NOL4 | | NM_003787.4 | | c.356C>T | p.Thr119Met |
| #5 | | missense_variant | | RHPN2 | | NM_033103.4 | | c.217G>A | p.Val73Met |
| #5 | | synonymous_variant | | ZNF45 | | NM_003425.3 | | c.891T>C | p.Val297Val |
| #5 | | intron_variant | | PVRL2 | | NM_001042724.1 | | c.1043-3608C>T | . |
| #5 | | synonymous_variant | | MYT1L | | NM_015025.2 | | c.2955C>T | p.Asp985Asp |
| #5 | | missense_variant | | FER1L5 | | NM_001293083.1 | | c.2663G>A | p.Arg888Gln |
| #5 | | missense_variant | | KCNH7 | | NM_033272.3 | | c.672A>T | p.Lys224Asn |
| #5 | | splice_acceptor_variant&intron_variant,intron_variant | | TTN,MIR548N | | NM_001267550.2,NR_031666.1 | | c.34856-1G>A,n.49-3770C>T | .,. |
| #5 | | missense_variant | | ASNSD1 | | NM_019048.2 | | c.1261C>A | p.Pro421Thr |
| #5 | | synonymous_variant | | SMARCAL1 | | NM_001127207.1 | | c.1440G>A | p.Pro480Pro |
| #5 | | synonymous_variant | | AQP12A | | NM_198998.2 | | c.249T>C | p.Thr83Thr |
| #5 | | intron_variant | | RALGAPA2 | | NM_020343.3 | | c.5053-42C>T | . |
| #5 | | missense_variant | | XKR7 | | NM_001011718.1 | | c.961G>A | p.Ala321Thr |
| #5 | | missense_variant | | TOX2 | | NM_001098797.1 | | c.1244C>G | p.Ala415Gly |
| #5 | | intron_variant | | SLC12A5 | | NM_001134771.1 | | c.122-5538C>T | . |
| #5 | | missense_variant | | CSE1L | | NM_001316.3 | | c.211A>G | p.Lys71Glu |
| #5 | | synonymous_variant | | ATP9A | | NM_006045.1 | | c.2070T>C | p.Asn690Asn |
| #5 | | missense_variant | | TPTE | | NM_199261.3 | | c.479T>A | p.Ile160Asn |
| #5 | | missense_variant | | SLC7A4 | | NM_004173.2 | | c.1354G>A | p.Val452Ile |
| #5 | | missense_variant | | PIWIL3 | | NM_001008496.3 | | c.728G>A | p.Arg243His |
| #5 | | 3_prime_UTR_variant | | RRP7A | | NM_015703.4 | | c.*19A>G | . |
| #5 | | missense_variant | | TCAIM | | NM_001282913.1 | | c.326G>C | p.Arg109Pro |
| #5 | | upstream_gene_variant,intron_variant | | MIR711,COL7A1 | | NR_031756.1,NM_000094.3 | | n.-1C>T,c.4819-46C>T | .,. |
| #5 | | missense_variant,upstream_gene_variant | | COL7A1,MIR711 | | NM_000094.3,NR_031756.1 | | c.4489C>T,n.-1C>T | p.Arg1497Cys,. |
| #5 | | synonymous_variant,upstream_gene_variant,intron_variant | | NAT6,IFRD2,HYAL3 | | NM_012191.3,NM_006764.4,NM_001200029.1 | | c.750T>A,c.-130T>A,c.-17-1161T>A | p.Thr250Thr,.,. |
| #5 | | stop_gained | | DOCK3 | | NM_004947.4 | | c.376C>T | p.Arg126* |
| #5 | | splice_region_variant&intron_variant | | FAM19A1 | | NM_001252216.1 | | c.259+8C>A | . |
| #5 | | missense_variant | | RGS12 | | NM_198229.2 | | c.3458G>T | p.Gly1153Val |
| #5 | | synonymous_variant | | EVC2 | | NM_147127.4 | | c.2415C>T | p.Ser805Ser |
| #5 | | missense_variant&splice_region_variant | | CPZ | | NM_001014447.2 | | c.1606C>A | p.Pro536Thr |
| #5 | | synonymous_variant | | HERC6 | | NM_017912.3 | | c.165G>A | p.Leu55Leu |
| #5 | | intron_variant | | OXCT1 | | NM_000436.3 | | c.955+26G>T | . |
| #5 | | intron_variant | | GPR98 | | NM_032119.3 | | c.8904-42T>A | . |
| #5 | | stop_gained | | APC | | NM_000038.5 | | c.904C>T | p.Arg302* |
| #5 | | stop_gained | | APC | | NM_000038.5 | | c.4033G>T | p.Glu1345* |
| #5 | | missense_variant,upstream_gene_variant | | PROB1,MZB1 | | NM_001161546.1,NM_016459.3 | | c.1088C>T,c.-61C>T | p.Pro363Leu,. |
| #5 | | missense_variant | | GNPDA1 | | NM_005471.4 | | c.560C>T | p.Thr187Met |
| #5 | | 3_prime_UTR_variant,upstream_gene_variant | | F12,PFN3 | | NM_000505.3,NM_001029886.2 | | c.*95G>A,c.-61G>A | .,. |
| #5 | | missense_variant | | EHMT2 | | NM_001289413.1.3 | | c.94C>G | p.Pro32Ala |
| #5 | | intron_variant | | PRIM2 | | NM_000947.4 | | c.834+16T>C | . |
| #5 | | intron_variant | | AHI1 | | NM_001134830.1 | | c.135+522C>A | . |
| #5 | | non_coding_exon_variant | | NCF1B | | NR_003186.1 | | n.491C>G | . |
| #5 | | missense_variant | | CLDN4 | | NM_001305.4 | | c.433C>A | p.Gln145Lys |
| #5 | | synonymous_variant | | POM121C | | NM_001099415.2 | | c.2346C>G | p.Ser782Ser |
| #5 | | stop_gained | | PEX1 | | NM_000466.2 | | c.547C>T | p.Arg183* |
| #5 | | missense_variant | | KPNA7 | | NM_001145715.1 | | c.1381G>A | p.Asp461Asn |
| #5 | | intron_variant | | LAMTOR4 | | NM_001008395.2 | | c.203-153A>G | . |
| #5 | | missense_variant | | FOXP2 | | NM_148898.3 | | c.1139G>A | p.Ser380Asn |
| #5 | | missense_variant | | KEL | | NM_000420.2 | | c.2024G>A | p.Arg675Gln |
| #5 | | synonymous_variant | | DLGAP2 | | NM_004745.4 | | c.96C>T | p.His32His |
| #5 | | missense_variant | | PPP1R3B | | NM_001201329.1 | | c.701G>A | p.Arg234Gln |
| #5 | | synonymous_variant | | BLK | | NM_001715.2 | | c.936C>T | p.Thr312Thr |
| #5 | | missense_variant | | UNC5D | | NM_080872.2 | | c.901T>G | p.Cys301Gly |
| #5 | | intergenic_region | | C8orf22-SNTG1 | | C8orf22-SNTG1 | | . | . |
| #5 | | missense_variant | | TNFRSF11B | | NM_002546.3 | | c.877G>A | p.Glu293Lys |
| #5 | | synonymous_variant | | BAI1 | | NM_001702.2 | | c.3093C>T | p.Thr1031Thr |
| #5 | | synonymous_variant | | SH3GL2 | | NM_003026.3 | | c.537G>A | p.Pro179Pro |
| #5 | | intergenic_region | | CNTNAP3B-LOC101927827 | | CNTNAP3B-LOC101927827 | | . | . |
| #5 | | missense_variant | | DAPK1 | | NM_001288729.1 | | c.3686G>A | p.Gly1229Glu |
| #5 | | missense_variant | | LAMC3 | | NM_006059.3 | | c.3896C>T | p.Ala1299Val |
| #5 | | 5_prime_UTR_variant | | PIGA | | NM_002641.3 | | c.-89G>A | . |
| #5 | | missense_variant | | CXorf23 | | NM_198279.3 | | c.554C>G | p.Ser185Cys |
| #5 | | synonymous_variant | | CXorf22 | | NM_152632.3 | | c.1386C>A | p.Gly462Gly |
| #5 | | 5_prime_UTR_variant | | FAM47C | | NM_001013736.2 | | c.-36G>A | . |
| #5 | | splice_region_variant&intron_variant | | ZNF182 | | NM_001178099.1 | | c.289+8G>A | . |
| #5 | | synonymous_variant | | HUWE1 | | NM_031407.6 | | c.10248C>T | p.Gly3416Gly |
| #5 | | intron_variant | | AR | | NM_000044.3 | | c.1769-5150T>C | . |
| #5 | | intron_variant,intron_variant | | BCYRN1,ZMYM3 | | NR_001568.1,NM_005096.3 | | n.173-30996G>A,c.3920+28G>A | .,. |
| #5 | | missense_variant | | PGK1 | | NM_000291.3 | | c.910G>T | p.Ala304Ser |
| #5 | | missense_variant | | IL13RA2 | | NM_000640.2 | | c.743G>A | p.Arg248Gln |
| #5 | | intron_variant | | MAP7D3 | | NM_024597.3 | | c.2140-18T>C | . |
| #8 | | missense_variant | | ATAD3B | | NM_031921.4 | | c.1798A>G | p.Thr600Ala |
| #8 | | stop_gained | | CACNA1E | | NM_001205293.1 | | c.6121C>T | p.Arg2041* |
| #8 | | synonymous_variant | | IRF6 | | NM_006147.3 | | c.135G>A | p.Arg45Arg |
| #8 | | missense_variant | | MYO7A | | NM_000260.3 | | c.1261C>T | p.Pro421Ser |
| #8 | | synonymous_variant | | FAM186A | | NM_001145475.1 | | c.3513T>C | p.Leu1171Leu |
| #8 | | missense_variant | | PCDH20 | | NM_022843.3 | | c.245T>A | p.Ile82Asn |
| #8 | | intergenic_region | | WHAMMP2-LOC100289656 | | WHAMMP2-LOC100289656 | | . | . |
| #8 | | 3_prime_UTR_variant | | YWHAE | | NM_006761.4 | | c.*57T>C | . |
| #8 | | intron_variant | | TOP3A | | NM_004618.3 | | c.391-9G>A | . |
| #8 | | non_coding_exon_variant | | LINC00470 | | NR_023925.1 | | n.379C>T | . |
| #8 | | synonymous_variant | | CCDC114 | | NM_144577.3 | | c.1086C>T | p.Ala362Ala |
| #8 | | intron_variant,intron_variant | | RNU6-81P,CCDC74A | | NR_046946.1,NM_138770.2 | | n.21-70026T>C,c.877-22T>C | .,. |
| #8 | | missense_variant | | ZNF217 | | NM_006526.2 | | c.2885C>T | p.Pro962Leu |
| #8 | | upstream_gene_variant,intron_variant | | RNF123,MST1 | | NM_022064.3,NM_020998.3 | | c.-127A>G,c.1251-9T>C | .,. |
| #8 | | missense_variant | | TTC14 | | NM_133462.3 | | c.364G>A | p.Asp122Asn |
| #8 | | intron_variant | | ZFYVE16 | | NM_001105251.2 | | c.70+23C>T | . |
| #8 | | missense_variant,upstream_gene_variant,intron_variant,intron_variant | | PCDHA3,PCDHA4,PCDHA1,PCDHA2 | | NM_018906.2,NM_018907.3,NM_018900.3,NM_018905.2 | | c.2104G>A,c.-115G>A,c.2394+14617G>A,c.2388+5949G>A | p.Val702Ile,.,.,. |
| #8 | | 3_prime_UTR_variant,non_coding_exon_variant | | POM121,NSUN5P2 | | NM_001257190.2,NR_033323.3 | | c.*1439C>G,n.868G>C | .,. |
| #8 | | stop_lost | | GTF2IRD1 | | NM_001199207.1 | | c.2931G>T | p.Ter977Tyrext*? |
| #8 | | missense_variant | | PTPRD | | NM_002839.3 | | c.653C>A | p.Ser218Tyr |
| #8 | | missense_variant | | BNC2 | | NM_017637.5 | | c.3233G>A | p.Arg1078Gln |
| #8 | | missense_variant,upstream_gene_variant | | MAGED2,SNORA11 | | NM_014599.5,NR_002953.1 | | c.634T>C,n.-1T>C | p.Ser212Pro,. |
| #8 | | missense_variant | | ATAD3B | | NM_031921.4 | | c.1798A>G | p.Thr600Ala |
| #8 | | missense_variant | | ATAD3B | | NM_031921.4 | | c.1810T>C | p.Tyr604His |
| #8 | | synonymous_variant | | HSPG2 | | NM_001291860.1 | | c.4932C>T | p.Pro1644Pro |
| #8 | | synonymous_variant | | HIVEP3 | | NM_024503.4 | | c.5526G>T | p.Val1842Val |
| #8 | | missense_variant | | LRRIQ3 | | NM_001105659.1 | | c.1205G>A | p.Arg402Gln |
| #8 | | missense_variant | | ADAMTSL4 | | NM_001288608.1 | | c.2378C>T | p.Pro793Leu |
| #8 | | intron_variant | | SMG5 | | NM_015327.2 | | c.1117+11C>G | . |
| #8 | | missense_variant | | PRCC | | NM_005973.4 | | c.370C>A | p.Pro124Thr |
| #8 | | stop_gained | | CACNA1E | | NM_001205293.1 | | c.6121C>T | p.Arg2041* |
| #8 | | synonymous_variant | | IRF6 | | NM_006147.3 | | c.135G>A | p.Arg45Arg |
| #8 | | missense_variant | | CAPN2 | | NM_001748.4 | | c.700A>G | p.Lys234Glu |
| #8 | | synonymous_variant | | PCDH15 | | NM_001142763.1 | | c.4326G>A | p.Pro1442Pro |
| #8 | | synonymous_variant,upstream_gene_variant | | KCNMA1,LOC101929328 | | NM_001161352.1,NR_120655.1 | | c.3480G>A,n.-1C>T | p.Pro1160Pro,. |
| #8 | | missense_variant | | DRD4 | | NM_000797.3 | | c.652G>A | p.Gly218Ser |
| #8 | | missense_variant | | ART1 | | NM_004314.2 | | c.403G>A | p.Val135Met |
| #8 | | stop_gained | | TTC17 | | NM_018259.5 | | c.1633G>T | p.Glu545* |
| #8 | | splice_region_variant&intron_variant | | FNBP4 | | NM_015308.2 | | c.1820+6C>T | . |
| #8 | | missense_variant | | FAM111A | | NM_001142519.1 | | c.129G>A | p.Met43Ile |
| #8 | | missense_variant | | ZBTB3 | | NM_024784.3 | | c.992C>A | p.Ala331Asp |
| #8 | | missense_variant,upstream_gene_variant | | ESRRA,PRDX5 | | NM_001282450.1,NM_012094.4 | | c.1055G>C,c.-129G>C | p.Arg352Pro,. |
| #8 | | missense_variant | | MYO7A | | NM_000260.3 | | c.1261C>T | p.Pro421Ser |
| #8 | | splice_region_variant&intron_variant | | CNTN5 | | NM_001243270.1 | | c.980+4A>G | . |
| #8 | | intron_variant | | KDM5A | | NM_001042603.2 | | c.4867-389G>A | . |
| #8 | | intron_variant | | LEPREL2 | | NM_014262.4 | | c.1263-17C>T | . |
| #8 | | stop_gained | | DUSP16 | | NM_030640.2 | | c.1447C>T | p.Arg483* |
| #8 | | intron_variant | | MUC19 | | NM_173600.2 | | c.23989+13C>T | . |
| #8 | | synonymous_variant | | FAM186A | | NM_001145475.1 | | c.3513T>C | p.Leu1171Leu |
| #8 | | missense_variant,intron_variant | | RFX4,LOC100287944 | | NM_001206691.1,NR_040246.1 | | c.1537G>A,n.142+41728C>T | p.Ala513Thr,. |
| #8 | | missense_variant | | LATS2 | | NM_014572.2 | | c.1741C>T | p.Arg581Cys |
| #8 | | missense_variant | | PCDH20 | | NM_022843.3 | | c.245T>A | p.Ile82Asn |
| #8 | | intergenic_region | | WHAMMP2-LOC100289656 | | WHAMMP2-LOC100289656 | | . | . |
| #8 | | missense_variant,intron_variant | | MAP1A,RNU6-28P | | NM_002373.5,NR_046489.1.2 | | c.2411C>T,n.35-78705C>T | p.Thr804Met,. |
| #8 | | splice_region_variant&intron_variant | | RSL24D1 | | NM_016304.2 | | c.419-4A>T | . |
| #8 | | synonymous_variant | | UACA | | NM_018003.2 | | c.2772C>T | p.Ser924Ser |
| #8 | | missense_variant | | RBFOX1 | | NM_145891.2 | | c.541G>A | p.Val181Ile |
| #8 | | intron_variant | | SNX29P1 | | NR_045011.1 | | n.107+33A>G | . |
| #8 | | intron_variant | | KATNB1 | | NM_005886.2 | | c.1177+31G>A | . |
| #8 | | intron_variant | | HYDIN | | NM_001270974.1 | | c.6532-62G>T | . |
| #8 | | missense_variant | | PHLPP2 | | NM_015020.3 | | c.629G>A | p.Arg210Gln |
| #8 | | 3_prime_UTR_variant | | YWHAE | | NM_006761.4 | | c.*57T>C | . |
| #8 | | missense_variant | | SGSM2 | | NM_014853.2 | | c.2021G>A | p.Arg674His |
| #8 | | stop_gained | | TP53 | | NM_000546.5 | | c.637C>T | p.Arg213* |
| #8 | | missense_variant | | TBC1D26 | | NM_178571.4 | | c.206G>A | p.Arg69His |
| #8 | | intron_variant | | TOP3A | | NM_004618.3 | | c.391-9G>A | . |
| #8 | | missense_variant,intron_variant | | SGK494,SPAG5-AS1 | | NM_001174103.1,NR_040012.1 | | c.244G>A,n.273-2765C>T | p.Glu82Lys,. |
| #8 | | intron_variant | | ACSF2 | | NM_001288968.1 | | c.529-11G>A | . |
| #8 | | intergenic_region | | ANKFN1-NOG | | ANKFN1-NOG | | . | . |
| #8 | | intron_variant,intron_variant | | LOC101927688,SEPT4 | | NR_110810.1,NM_001256782.1 | | n.258+1317G>T,c.106-156C>A | .,. |
| #8 | | synonymous_variant | | BAIAP2 | | NM_017451.2 | | c.1365G>A | p.Thr455Thr |
| #8 | | splice_region_variant&intron_variant | | FASN | | NM_004104.4 | | c.778+6C>T | . |
| #8 | | non_coding_exon_variant | | LINC00470 | | NR_023925.1 | | n.379C>T | . |
| #8 | | synonymous_variant | | ABHD17A | | NM_031213.3 | | c.513T>C | p.Asn171Asn |
| #8 | | synonymous_variant | | CD209 | | NM_021155.3 | | c.1098C>T | p.Asp366Asp |
| #8 | | missense_variant | | MUC16 | | NM_024690.2 | | c.874G>A | p.Asp292Asn |
| #8 | | missense_variant&splice_region_variant | | MRPL4 | | NM_015956.2 | | c.555C>A | p.Asp185Glu |
| #8 | | intron_variant | | MAST3 | | NM_015016.1 | | c.2118+48A>G | . |
| #8 | | synonymous_variant,upstream_gene_variant | | OVOL3,TBCB | | NM_001270948.1,NM_001281.2 | | c.111G>A,c.-576G>A | p.Val37Val,. |
| #8 | | missense_variant | | CAPN12 | | NM_144691.3 | | c.412G>A | p.Val138Ile |
| #8 | | intron_variant | | XRCC1 | | NM_006297.2 | | c.1621+16A>G | . |
| #8 | | synonymous_variant | | CCDC114 | | NM_144577.3 | | c.1086C>T | p.Ala362Ala |
| #8 | | missense_variant | | KLK13 | | NM_015596.1 | | c.788G>T | p.Arg263Leu |
| #8 | | synonymous_variant | | ZNF865 | | NM_001195605.1 | | c.2025C>T | p.Gly675Gly |
| #8 | | missense_variant | | APOB | | NM_000384.2 | | c.9491C>T | p.Thr3164Met |
| #8 | | intron_variant | | DRC1 | | NM_145038.3 | | c.1510-49G>A | . |
| #8 | | missense_variant | | EML6 | | NM_001039753.2 | | c.5677G>A | p.Ala1893Thr |
| #8 | | intron_variant | | VWA3B | | NM_144992.4 | | c.2674-4762A>T | . |
| #8 | | synonymous_variant | | GLI2 | | NM_005270.4 | | c.4260G>A | p.Ala1420Ala |
| #8 | | intron_variant,intron_variant | | RNU6-81P,CCDC74A | | NR_046946.1,NM_138770.2 | | n.21-70026T>C,c.877-22T>C | .,. |
| #8 | | stop_gained | | THSD7B | | NM_001080427.1 | | c.4341C>A | p.Cys1447* |
| #8 | | synonymous_variant | | CHRND | | NM_000751.2 | | c.663C>T | p.Asn221Asn |
| #8 | | intron_variant | | PIGU | | NM_080476.4 | | c.627+90T>A | . |
| #8 | | synonymous_variant | | CHD6 | | NM_032221.4 | | c.2493G>A | p.Gly831Gly |
| #8 | | missense_variant | | ZNF217 | | NM_006526.2 | | c.2885C>T | p.Pro962Leu |
| #8 | | missense_variant | | ZNF831 | | NM_178457.2 | | c.3215G>A | p.Ser1072Asn |
| #8 | | missense_variant,upstream_gene_variant | | CRELD2,ALG12 | | NM_001135101.2,NM_024105.3 | | c.236T>C,c.-275A>G | p.Leu79Pro,. |
| #8 | | intron_variant | | ZNF385D | | NM_024697.2 | | c.277-747C>A | . |
| #8 | | upstream_gene_variant,intron_variant | | RNF123,MST1 | | NM_022064.3,NM_020998.3 | | c.-127A>G,c.1251-9T>C | .,. |
| #8 | | splice_acceptor_variant&intron_variant | | SLC9A9 | | NM_173653.3 | | c.1470-2A>G | . |
| #8 | | missense_variant | | TTC14 | | NM_133462.3 | | c.364G>A | p.Asp122Asn |
| #8 | | missense_variant | | MAP3K13 | | NM_001242314.1 | | c.248G>A | p.Ser83Asn |
| #8 | | missense_variant | | CCNA2 | | NM_001237.3 | | c.1009G>A | p.Gly337Arg |
| #8 | | intron_variant | | RNF150 | | NM_020724.1 | | c.987+52C>A | . |
| #8 | | missense_variant | | DNAH5 | | NM_001369.2 | | c.6715C>A | p.Pro2239Thr |
| #8 | | missense_variant | | ANKRD31 | | NM_001164443.1 | | c.1781A>G | p.Glu594Gly |
| #8 | | intron_variant | | ZFYVE16 | | NM_001105251.2 | | c.70+23C>T | . |
| #8 | | missense_variant,upstream_gene_variant,intron_variant,intron_variant | | PCDHA3,PCDHA4,PCDHA1,PCDHA2 | | NM_018906.2,NM_018907.3,NM_018900.3,NM_018905.2 | | c.2104G>A,c.-115G>A,c.2394+14617G>A,c.2388+5949G>A | p.Val702Ile,.,.,. |
| #8 | | splice_region_variant&intron_variant | | CDHR2 | | NM_001171976.1 | | c.622-4G>A | . |
| #8 | | intron_variant | | GRM6 | | NM_000843.3 | | c.1355-426C>T | . |
| #8 | | intron_variant | | GCNT2 | | NM_145649.4 | | c.925+26774A>G | . |
| #8 | | synonymous_variant | | SOX4 | | NM_003107.2 | | c.1011C>T | p.Pro337Pro |
| #8 | | missense_variant | | MUC22 | | NM_001198815.1.7 | | c.4088C>A | p.Ser1363Tyr |
| #8 | | 5_prime_UTR_premature_start_codon_gain_variant,5_prime_UTR_variant | | BRD2,BRD2 | | NM_001199455.1.5,NM_001199455.1.5 | | c.-799G>T,c.-799G>T | .,. |
| #8 | | missense_variant | | EFHC1 | | NM_018100.3 | | c.1055G>A | p.Arg352Gln |
| #8 | | missense_variant | | KBTBD2 | | NM_015483.2 | | c.755C>T | p.Ser252Phe |
| #8 | | missense_variant | | COBL | | NM_001287436.1 | | c.353T>A | p.Phe118Tyr |
| #8 | | 3_prime_UTR_variant,non_coding_exon_variant | | POM121,NSUN5P2 | | NM_001257190.2,NR_033323.3 | | c.*1439C>G,n.868G>C | .,. |
| #8 | | stop_lost | | GTF2IRD1 | | NM_001199207.1 | | c.2931G>T | p.Ter977Tyrext*? |
| #8 | | intron_variant | | ATXN7L1 | | NM_020725.1 | | c.1517+2181C>A | . |
| #8 | | intron_variant | | CNTNAP2 | | NM_014141.5 | | c.1083+16C>T | . |
| #8 | | missense_variant | | SSPO | | NM_198455.2 | | c.4304G>C | p.Cys1435Ser |
| #8 | | missense_variant | | LZTS1 | | NM_021020.3 | | c.107G>A | p.Arg36Gln |
| #8 | | missense_variant | | HR | | NM_005144.4 | | c.1784G>A | p.Gly595Asp |
| #8 | | 5_prime_UTR_variant | | LOXL2 | | NM_002318.2 | | c.-60C>A | . |
| #8 | | intron_variant | | ELP3 | | NM_018091.5 | | c.1192-10T>A | . |
| #8 | | synonymous_variant | | PXDNL | | NM_144651.4 | | c.3252G>A | p.Ala1084Ala |
| #8 | | missense_variant | | PTPRD | | NM_002839.3 | | c.653C>A | p.Ser218Tyr |
| #8 | | missense_variant | | BNC2 | | NM_017637.5 | | c.3233G>A | p.Arg1078Gln |
| #8 | | missense_variant | | SPATA31D1 | | NM_001001670.2 | | c.4588A>C | p.Thr1530Pro |
| #8 | | intergenic_region | | LINC00475-IARS | | LINC00475-IARS | | . | . |
| #8 | | synonymous_variant | | ABCA1 | | NM_005502.3 | | c.3834G>A | p.Lys1278Lys |
| #8 | | missense_variant | | CDK5RAP2 | | NM_018249.5 | | c.1643C>T | p.Ser548Leu |
| #8 | | intron_variant | | C5 | | NM_001735.2 | | c.4398+43C>A | . |
| #8 | | missense_variant | | GSN | | NM_000177.4 | | c.1942A>G | p.Lys648Glu |
| #8 | | intron_variant | | MAPKAP1 | | NM_001006617.1 | | c.1346-2785A>G | . |
| #8 | | synonymous_variant | | LCN9 | | NM_001001676.1 | | c.66C>T | p.Thr22Thr |
| #8 | | missense_variant&splice_region_variant | | EXD3 | | NM_017820.4 | | c.1040C>T | p.Ala347Val |
| #8 | | missense_variant,upstream_gene_variant | | MAGED2,SNORA11 | | NM_014599.5,NR_002953.1 | | c.634T>C,n.-1T>C | p.Ser212Pro,. |
| #8 | | synonymous_variant | | TRO | | NM_001039705.2 | | c.2022G>T | p.Val674Val |
| #8 | | missense_variant | | MAMLD1 | | NM_001177465.2 | | c.2378C>T | p.Ser793Leu |
| #9 | | intron_variant | | EIF4G3 | | NM_001198801.1 | | c.546+69C>G | . |
| #9 | | missense_variant | | ATP8B2 | | NM_020452.3 | | c.856A>G | p.Thr286Ala |
| #9 | | intragenic_variant | | DGKZ | | DGKZ | | . | . |
| #9 | | 3_prime_UTR_variant | | PSPC1 | | NM_001042414.2 | | c.*22T>C | . |
| #9 | | missense_variant&splice_region_variant,upstream_gene_variant | | DHRS4,DHRS4-AS1 | | NM_021004.3,NR_023921.2 | | c.305C>T,n.-1G>A | p.Thr102Met,. |
| #9 | | missense_variant | | CSPG4 | | NM_001897.4 | | c.1321G>A | p.Glu441Lys |
| #9 | | splice_region_variant&intron_variant | | XAF1 | | NM_017523.3 | | c.226-3C>A | . |
| #9 | | synonymous_variant | | EVPLL | | NM_001145127.1 | | c.888A>G | p.Pro296Pro |
| #9 | | intergenic_region | | FAM90A27P-BIRC8 | | FAM90A27P-BIRC8 | | . | . |
| #9 | | missense_variant | | LRP1B | | NM_018557.2 | | c.7603C>T | p.His2535Tyr |
| #9 | | 3_prime_UTR_variant,upstream_gene_variant | | ID1,MIR3193 | | NM_002165.3,NR_036161.1 | | c.*38A>G,n.-1A>G | .,. |
| #9 | | intron_variant | | ABHD16A | | NM_021160.2 | | c.741+51A>C | . |
| #9 | | missense_variant | | PACRG | | NM_152410.2 | | c.479G>A | p.Arg160Gln |
| #9 | | non_coding_exon_variant | | GUSBP10 | | NR_030766.1 | | n.145G>A | . |
| #9 | | missense_variant | | DPP6 | | NM_130797.3 | | c.191G>A | p.Gly64Asp |
| #9 | | intron_variant | | CYP11B1 | | NM_000497.3 | | c.396-612A>G | . |
| #9 | | 5_prime_UTR_premature_start_codon_gain_variant,5_prime_UTR_variant,non_coding_exon_variant | | TNFRSF14,TNFRSF14,LOC115110 | | NM_003820.3,NM_003820.3,NR_037844.2 | | c.-192C>G,c.-192C>G,n.259G>C | .,.,. |
| #9 | | splice_region_variant&intron_variant | | TNFRSF25 | | NM_148965.1 | | c.952+5G>C | . |
| #9 | | intron_variant | | EIF4G3 | | NM_001198801.1 | | c.546+69C>G | . |
| #9 | | intron_variant | | TSPAN1 | | NM_005727.3 | | c.-142+1005G>A | . |
| #9 | | intron_variant | | AK5 | | NM_174858.2 | | c.699+15809A>G | . |
| #9 | | missense_variant | | ATP8B2 | | NM_020452.3 | | c.856A>G | p.Thr286Ala |
| #9 | | missense_variant | | SPTA1 | | NM_003126.2 | | c.5195A>C | p.Lys1732Thr |
| #9 | | missense_variant | | ITLN1 | | NM_017625.2 | | c.776G>A | p.Cys259Tyr |
| #9 | | intron_variant | | RCSD1 | | NM_052862.3 | | c.1219-87C>G | . |
| #9 | | missense_variant | | PRRC2C | | NM_015172.3 | | c.5465C>T | p.Ser1822Phe |
| #9 | | missense_variant | | ZNF648 | | NM_001009992.1 | | c.1571G>A | p.Arg524Gln |
| #9 | | missense_variant | | NUAK2 | | NM_030952.1 | | c.1105G>C | p.Glu369Gln |
| #9 | | missense_variant | | TARBP1 | | NM_005646.3 | | c.1519G>A | p.Gly507Arg |
| #9 | | stop_gained | | C1orf101 | | NM_001130957.1 | | c.2803C>T | p.Arg935* |
| #9 | | missense_variant | | PTER | | NM_001001484.2 | | c.589C>G | p.Pro197Ala |
| #9 | | synonymous_variant | | KIAA1217 | | NM_019590.4 | | c.2685G>A | p.Gln895Gln |
| #9 | | missense_variant | | RTKN2 | | NM_145307.3 | | c.1240C>G | p.Arg414Gly |
| #9 | | intron_variant | | TBATA | | NM_152710.2 | | c.970+232C>T | . |
| #9 | | stop_gained | | PDCD4 | | NM_014456.4 | | c.124G>T | p.Gly42* |
| #9 | | stop_gained | | PDZD8 | | NM_173791.3 | | c.1736C>G | p.Ser579* |
| #9 | | synonymous_variant | | C10orf91 | | NM_173541.2 | | c.249G>A | p.Gln83Gln |
| #9 | | stop_gained | | OR52B2 | | NM_001004052.1 | | c.457C>T | p.Arg153* |
| #9 | | missense_variant | | PHF21A | | NM_001101802.1 | | c.1955C>T | p.Pro652Leu |
| #9 | | intragenic_variant | | DGKZ | | DGKZ | | . | . |
| #9 | | missense_variant | | OR8H2 | | NM_001005200.1 | | c.329C>A | p.Ala110Asp |
| #9 | | intron_variant | | TREH | | NM_007180.2 | | c.735-51G>T | . |
| #9 | | splice_region_variant&intron_variant | | ADAMTS15 | | NM_139055.2 | | c.2079-6C>T | . |
| #9 | | missense_variant | | IGSF9B | | NM_001277285.1 | | c.2685G>C | p.Glu895Asp |
| #9 | | intergenic_region | | DDX12P-KLRB1 | | DDX12P-KLRB1 | | . | . |
| #9 | | splice_region_variant&intron_variant | | MUC19 | | NM_173600.2 | | c.5702-5A>C | . |
| #9 | | intron_variant | | AQP6 | | NM_001652.3 | | c.642+11C>T | . |
| #9 | | synonymous_variant | | ESPL1 | | NM_012291.4 | | c.1926G>T | p.Thr642Thr |
| #9 | | missense_variant | | IKZF4 | | NM_022465.3 | | c.806G>A | p.Arg269Gln |
| #9 | | missense_variant | | NAB2 | | NM_005967.3 | | c.1384G>C | p.Glu462Gln |
| #9 | | intergenic_region | | ASCL1-C12orf42 | | ASCL1-C12orf42 | | . | . |
| #9 | | intron_variant | | NOS1 | | NM_001204218.1 | | c.3507+18G>A | . |
| #9 | | 3_prime_UTR_variant | | PSPC1 | | NM_001042414.2 | | c.*22T>C | . |
| #9 | | synonymous_variant | | XPO4 | | NM_022459.4 | | c.3084C>A | p.Ala1028Ala |
| #9 | | missense_variant | | GPR12 | | NM_005288.3 | | c.161C>T | p.Ser54Leu |
| #9 | | missense_variant | | MTUS2 | | NM_001033602.2 | | c.2222A>C | p.Lys741Thr |
| #9 | | 5_prime_UTR_variant | | MYO16 | | NM_001198950.1 | | c.-29G>A | . |
| #9 | | missense_variant | | MYH7 | | NM_000257.3 | | c.974A>G | p.Asp325Gly |
| #9 | | synonymous_variant,upstream_gene_variant | | DHRS4,DHRS4-AS1 | | NM_021004.3,NR_023921.2 | | c.231G>A,n.-1C>T | p.Gln77Gln,. |
| #9 | | missense_variant&splice_region_variant,upstream_gene_variant | | DHRS4,DHRS4-AS1 | | NM_021004.3,NR_023921.2 | | c.305C>T,n.-1G>A | p.Thr102Met,. |
| #9 | | stop_gained,intron_variant | | PRKD1,MIR548AI | | NM_002742.2,NR_039672.1 | | c.763C>T,n.29+66536C>T | p.Arg255*,. |
| #9 | | intergenic_region | | EGLN3-SPTSSA | | EGLN3-SPTSSA | | . | . |
| #9 | | intron_variant | | RBM25 | | NM_021239.2 | | c.156+30G>C | . |
| #9 | | splice_donor_variant&intron_variant | | PPP4R4 | | NM_058237.1 | | c.2197+1G>A | . |
| #9 | | upstream_gene_variant,intergenic_region | | SNORD114-31,SNORD114-30-SNORD114-31 | | NR_003224.1,SNORD114-30-SNORD114-31 | | n.-1G>A,. | .,. |
| #9 | | upstream_gene_variant,upstream_gene_variant,intergenic_region | | MIR656,MEG9,MIR410-MIR656 | | NR_030392.1,NR_047664.1,MIR410-MIR656 | | n.-1G>A,n.-1G>A,. | .,.,. |
| #9 | | missense_variant | | JAG2 | | NM_002226.4 | | c.2057G>A | p.Arg686His |
| #9 | | missense_variant | | BCL2L10 | | NM_020396.2 | | c.226G>A | p.Glu76Lys |
| #9 | | missense_variant | | CYP11A1 | | NM_000781.2 | | c.574G>A | p.Gly192Arg |
| #9 | | missense_variant | | CSPG4 | | NM_001897.4 | | c.1321G>A | p.Glu441Lys |
| #9 | | missense_variant | | EFTUD1 | | NM_024580.5 | | c.278C>G | p.Ser93Cys |
| #9 | | missense_variant | | MESP1 | | NM_018670.3 | | c.260C>A | p.Ala87Asp |
| #9 | | intron_variant | | BLM | | NM_000057.3 | | c.2074+14G>A | . |
| #9 | | missense_variant | | CAPN15 | | NM_005632.2 | | c.169A>T | p.Asn57Tyr |
| #9 | | synonymous_variant | | CAPN15 | | NM_005632.2 | | c.175C>T | p.Leu59Leu |
| #9 | | 5_prime_UTR_variant,upstream_gene_variant | | NHLRC4,PIGQ | | NM_001301159.1,NM_148920.2 | | c.-455G>T,c.-139G>T | .,. |
| #9 | | intron_variant | | PIGQ | | NM_148920.2 | | c.1531+335G>C | . |
| #9 | | missense_variant | | CHD9 | | NM_025134.4 | | c.244G>A | p.Val82Ile |
| #9 | | upstream_gene_variant,intron_variant,intron_variant | | EMC6,TAX1BP3,P2RX5-TAX1BP3 | | NM_001014764.2,NM_014604.3,NR_037928.1 | | c.-340G>C,c.159+30C>G,n.5214+30C>G | .,.,. |
| #9 | | splice_region_variant&intron_variant | | XAF1 | | NM_017523.3 | | c.226-3C>A | . |
| #9 | | stop_gained | | TP53 | | NM_000546.5 | | c.916C>T | p.Arg306* |
| #9 | | intron_variant | | TOP3A | | NM_004618.3 | | c.1711+10G>A | . |
| #9 | | synonymous_variant | | EVPLL | | NM_001145127.1 | | c.888A>G | p.Pro296Pro |
| #9 | | missense_variant | | AATF | | NM_012138.3 | | c.142G>A | p.Gly48Ser |
| #9 | | missense_variant | | ERBB2 | | NM_004448.3 | | c.994G>C | p.Glu332Gln |
| #9 | | upstream_gene_variant,intron_variant | | MIR6867,RAPGEFL1 | | NR_106927.1,NM_016339.3 | | n.-1C>G,c.216-76C>G | .,. |
| #9 | | missense_variant | | SRSF1 | | NM_006924.4 | | c.43G>C | p.Asp15His |
| #9 | | stop_gained&splice_region_variant | | LOXHD1 | | NM_144612.6 | | c.1270A>T | p.Lys424* |
| #9 | | missense_variant | | RTTN | | NM_173630.3 | | c.199C>G | p.Leu67Val |
| #9 | | missense_variant | | TCF3 | | NM_003200.3 | | c.1030C>T | p.His344Tyr |
| #9 | | intron_variant | | FBN3 | | NM_032447.3 | | c.6032-31C>A | . |
| #9 | | missense_variant | | FFAR3 | | NM_005304.3 | | c.154G>A | p.Val52Met |
| #9 | | missense_variant | | PROSER3 | | NM_001039887.2 | | c.514G>C | p.Ala172Pro |
| #9 | | missense_variant | | CLASRP | | NM_007056.2 | | c.584C>T | p.Ser195Leu |
| #9 | | missense_variant | | FBXO46 | | NM_001080469.1 | | c.1310C>T | p.Ala437Val |
| #9 | | missense_variant | | ZNF528 | | NM_032423.2 | | c.949A>C | p.Lys317Gln |
| #9 | | intergenic_region | | FAM90A27P-BIRC8 | | FAM90A27P-BIRC8 | | . | . |
| #9 | | missense_variant | | TMC4 | | NM_001145303.2 | | c.745C>T | p.Arg249Cys |
| #9 | | synonymous_variant,upstream_gene_variant | | TSEN34,MBOAT7 | | NM_001282333.1,NM_024298.4 | | c.933A>G,c.-549T>C | p.Val311Val,. |
| #9 | | synonymous_variant | | FAM71E2 | | NM_001145402.1 | | c.528G>A | p.Pro176Pro |
| #9 | | synonymous_variant | | USP29 | | NM_020903.2 | | c.2571C>T | p.Asn857Asn |
| #9 | | intron_variant | | RNF181 | | NM_016494.3 | | c.218-64C>T | . |
| #9 | | synonymous_variant | | KIAA1211L | | NM_207362.2 | | c.1872C>T | p.His624His |
| #9 | | missense_variant | | AFF3 | | NM_001025108.1 | | c.116G>A | p.Ser39Asn |
| #9 | | intron_variant | | ZC3H6 | | NM_198581.2 | | c.214-13C>T | . |
| #9 | | synonymous_variant | | MYO7B | | NM_001080527.1 | | c.231C>T | p.Asn77Asn |
| #9 | | missense_variant | | LRP1B | | NM_018557.2 | | c.7603C>T | p.His2535Tyr |
| #9 | | missense_variant | | NEB | | NM_001271208.1 | | c.21175C>G | p.Leu7059Val |
| #9 | | missense_variant | | TTC21B | | NM_024753.4 | | c.3121G>C | p.Asp1041His |
| #9 | | splice_region_variant&intron_variant | | STK39 | | NM_013233.2 | | c.1243-8C>T | . |
| #9 | | splice_donor_variant&intron_variant | | CPS1 | | NM_001122633.2 | | c.3354+1G>C | . |
| #9 | | splice_region_variant&intron_variant | | ABCA12 | | NM_173076.2 | | c.317+7G>T | . |
| #9 | | missense_variant | | SP100 | | NM_001080391.1 | | c.628G>A | p.Glu210Lys |
| #9 | | missense_variant | | PLCB4 | | NM_000933.3 | | c.3149T>G | p.Leu1050Arg |
| #9 | | synonymous_variant | | RRBP1 | | NM_001042576.1 | | c.658C>T | p.Leu220Leu |
| #9 | | intragenic_variant | | DEFB119 | | DEFB119 | | . | . |
| #9 | | synonymous_variant,upstream_gene_variant | | REM1,LINC00028 | | NM_014012.5,NR_024358.1 | | c.681G>A,n.-1G>A | p.Thr227Thr,. |
| #9 | | 3_prime_UTR_variant,upstream_gene_variant | | ID1,MIR3193 | | NM_002165.3,NR_036161.1 | | c.*38A>G,n.-1A>G | .,. |
| #9 | | missense_variant&splice_region_variant | | MYLK2 | | NM_033118.3 | | c.1423C>G | p.Leu475Val |
| #9 | | missense_variant | | ZMYND8 | | NM_001281775.2 | | c.1664C>T | p.Ser555Leu |
| #9 | | missense_variant | | TCFL5 | | NM_006602.2 | | c.406G>C | p.Glu136Gln |
| #9 | | stop_gained | | TCFL5 | | NM_006602.2 | | c.214G>T | p.Glu72* |
| #9 | | intron_variant | | APP | | NM_000484.3 | | c.1224+9G>C | . |
| #9 | | synonymous_variant | | MEI1 | | NM_152513.3 | | c.1020C>T | p.Leu340Leu |
| #9 | | intron_variant | | SHANK3 | | NM_033517.1 | | c.2068+84G>A | . |
| #9 | | synonymous_variant | | ACR | | NM_001097.2 | | c.459G>A | p.Ser153Ser |
| #9 | | missense_variant | | FGD5 | | NM_152536.3 | | c.1386G>C | p.Leu462Phe |
| #9 | | intron_variant | | LZTFL1 | | NM_020347.3 | | c.128+28A>C | . |
| #9 | | synonymous_variant | | XCR1 | | NM_001024644.1 | | c.96C>T | p.Leu32Leu |
| #9 | | intron_variant | | ZNF589 | | NM_016089.2 | | c.96+123G>A | . |
| #9 | | upstream_gene_variant,intron_variant | | CELSR3,CELSR3-AS1 | | NM_001407.2,NR_111921.1 | | c.-282G>C,n.46+349C>G | .,. |
| #9 | | intron_variant | | DOCK3 | | NM_004947.4 | | c.4108-16C>T | . |
| #9 | | missense_variant,upstream_gene_variant | | H1FX,H1FX-AS1 | | NM_006026.3,NR_026991.1 | | c.233C>G,n.-1G>C | p.Pro78Arg,. |
| #9 | | missense_variant | | PIK3CA | | NM_006218.2 | | c.3140A>T | p.His1047Leu |
| #9 | | intron_variant | | LIMCH1 | | NM_014988.3 | | c.936-9367G>C | . |
| #9 | | intron_variant | | LNX1 | | NM_001126328.2 | | c.380+15693C>G | . |
| #9 | | missense_variant&splice_region_variant | | KIAA1109 | | NM_015312.3 | | c.5923G>C | p.Asp1975His |
| #9 | | missense_variant | | PCDH10 | | NM_032961.1 | | c.1541C>A | p.Thr514Asn |
| #9 | | intron_variant | | ARHGAP10 | | NM_024605.3 | | c.2272+22G>A | . |
| #9 | | missense_variant | | TCF7 | | NM_003202.3 | | c.340A>G | p.Ser114Gly |
| #9 | | missense_variant | | GEMIN5 | | NM_015465.4 | | c.2885T>A | p.Leu962Gln |
| #9 | | intron_variant | | HNRNPH1 | | NM_001257293.1 | | c.-31-26C>G | . |
| #9 | | missense_variant&splice_region_variant,intron_variant | | LOC100130357,PHACTR1 | | NM_001242698.1,NM_001242648.1 | | c.143G>A,c.1510-1871C>T | p.Arg48Gln,. |
| #9 | | intron_variant | | ABHD16A | | NM_021160.2 | | c.741+51A>C | . |
| #9 | | missense_variant,missense_variant,missense_variant | | C4A,C4B,C4B_2 | | NM_007293.2.4,NM_001002029.3,NM_001242823.2.8 | | c.2743G>T,c.2743G>T,c.2743G>T | p.Ala915Ser,p.Ala915Ser,p.Ala915Ser |
| #9 | | missense_variant,intron_variant | | LOC441155,EYS | | NM_001271675.1,NM_001292009.1 | | c.899A>G,c.1767-7301T>C | p.Lys300Arg,. |
| #9 | | splice_region_variant&intron_variant | | C6orf163 | | NM_001010868.2 | | c.555-3T>C | . |
| #9 | | missense_variant | | ASCC3 | | NM_006828.3 | | c.5521A>T | p.Ser1841Cys |
| #9 | | missense_variant | | TRDN | | NM_006073.3 | | c.1754G>A | p.Arg585Gln |
| #9 | | intron_variant | | MOXD1 | | NM_015529.3 | | c.264+8978G>T | . |
| #9 | | missense_variant | | SASH1 | | NM_015278.3 | | c.2615C>T | p.Thr872Met |
| #9 | | missense_variant | | PACRG | | NM_152410.2 | | c.479G>A | p.Arg160Gln |
| #9 | | intron_variant | | TRG-AS1 | | NR_040085.1 | | n.435+8401G>C | . |
| #9 | | intron_variant | | AMPH | | NM_001635.3 | | c.1158+93G>T | . |
| #9 | | non_coding_exon_variant | | GUSBP10 | | NR_030766.1 | | n.145G>A | . |
| #9 | | missense_variant | | DPP6 | | NM_130797.3 | | c.191G>A | p.Gly64Asp |
| #9 | | intron_variant | | XKR4 | | NM_052898.1 | | c.806+37004G>A | . |
| #9 | | missense_variant | | PI15 | | NM_015886.3 | | c.685T>C | p.Cys229Arg |
| #9 | | intron_variant | | CYP11B1 | | NM_000497.3 | | c.396-612A>G | . |
| #9 | | synonymous_variant | | KIAA1161 | | NM_020702.4 | | c.1497G>A | p.Ser499Ser |
| #9 | | non_coding_exon_variant | | FAM205B | | NR_024481.1 | | n.914G>A | . |
| #9 | | synonymous_variant | | MAMDC2 | | NM_153267.4 | | c.273G>A | p.Ser91Ser |
| #9 | | missense_variant | | MAP3K15 | | NM_001001671.3 | | c.1531T>G | p.Phe511Val |
| #9 | | missense_variant | | KLHL34 | | NM_153270.1 | | c.1787A>G | p.Asp596Gly |
| #9 | | synonymous_variant | | PORCN | | NM_203475.2 | | c.432C>T | p.Gly144Gly |
| #9 | | non_coding_exon_variant | | XIST | | NR_001564.2 | | n.1199C>T | . |
| #9 | | intron_variant | | ATRX | | NM_000489.4 | | c.5786+68T>C | . |
| #9 | | synonymous_variant,upstream_gene_variant | | COX7B,MAGT1 | | NM_001866.2,NM_032121.5 | | c.21C>T,c.-63G>A | p.Ser7Ser,. |
| #9 | | missense_variant | | P2RY10 | | NM_014499.2 | | c.789T>A | p.Phe263Leu |
| #9 | | 5_prime_UTR_variant | | CYLC1 | | NM_021118.2 | | c.-16C>A | . |
| #9 | | missense_variant,missense_variant | | NXF2B,NXF2 | | NM_001099686.2.2,NM_022053.3 | | c.602C>T,c.602C>T | p.Ala201Val,p.Ala201Val |
| #9 | | synonymous_variant | | HS6ST2 | | NM_001077188.1 | | c.1254T>C | p.Phe418Phe |
| #9 | | synonymous_variant | | HS6ST2 | | NM_001077188.1 | | c.1236C>T | p.Gly412Gly |
| #9 | | missense_variant | | ATP11C | | NM_173694.4 | | c.2303A>G | p.Asp768Gly |
| #9 | | stop_gained | | UBE2NL | | NM_001012989.2 | | c.37C>T | p.Gln13* |
| #9 | synonymous_variant,upstream_gene_variant | | GDI1,FAM50A | | NM_001493.2,NM_004699.3 | | c.936C>T,c.-111C>T | | p.Asn312Asn,. |

Supplementary Table S4 List of mutations (INDELs) in primary CRC tumours.

|  | **Var_type** | **Consequences** | **Gene** | **Transcript_id** | **HGVS_P** |
| --- | --- | --- | --- | --- | --- |
| #5 | DEL | intron_variant | C1orf168 | NM_001004303.4 | . |
| #5 | DEL | intron_variant | GPR158 | NM_020752.2 | . |
| #5 | DEL | intron_variant | TRPM5 | NM_014555.3 | . |
| #5 | DEL | intron_variant | NUP98 | NM_016320.4 | . |
| #5 | INS | intron_variant | CAMKK2 | NM_001270485.1 | . |
| #5 | DEL | intron_variant | CCNA1 | NM_003914.3 | . |
| #5 | DEL | intron_variant | SOS2 | NM_006939.2 | . |
| #5 | DEL | splice_region_variant&intron_variant,intron_variant | TP53BP1,RNU6-28P | NM_001141980.1,NR_046489.1.2 | .,. |
| #5 | DEL | 5_prime_UTR_variant,upstream_gene_variant,intergenic_region,transcript | RHOT1,RHOT1,LRRC37B-RHOT1,RHOT1 | NM_001033568.2,NM_001033568.2,LRRC37B-RHOT1,NM_001033568.2 | .,.,.,. |
| #5 | DEL | intron_variant | PSMD11 | NM_001270482.1 | . |
| #5 | DEL | upstream_gene_variant,intergenic_region | KLK1,KLK1-KLK15 | NM_002257.3,KLK1-KLK15 | .,. |
| #5 | DEL | intergenic_region | SLC4A3-MIR4268 | SLC4A3-MIR4268 | . |
| #5 | DEL | splice_region_variant&intron_variant,upstream_gene_variant | CRELD2,ALG12 | NM_001135101.2,NM_024105.3 | .,. |
| #5 | DEL | 3_prime_UTR_variant | PLRG1 | NM_002669.3 | . |
| #5 | DEL | intergenic_region | IRX1-LOC101929153 | IRX1-LOC101929153 | . |
| #5 | DEL | intron_variant | RNASET2 | NM_003730.4 | . |
| #5 | DEL | intron_variant | IGFBP3 | NM_001013398.1 | . |
| #5 | DEL | intron_variant | LRRC69 | NM_001129890.1 | . |
| #5 | DEL | intron_variant | CSMD3 | NM_198123.1 | . |
| #5 | INS | intron_variant | ADAMTSL1 | NM_001040272.5 | . |
| #5 | DEL | 3_prime_UTR_variant | SYTL4 | NM_001129896.2 | . |
| #5 | INS | intron_variant | SNRNP40 | NM_004814.2 | . |
| #5 | DEL | intron_variant | C1orf168 | NM_001004303.4 | . |
| #5 | INS | intron_variant | LYST | NM_000081.3 | . |
| #5 | DEL | intron_variant | GPR158 | NM_020752.2 | . |
| #5 | DEL | intron_variant | TRPM5 | NM_014555.3 | . |
| #5 | DEL | intron_variant | NUP98 | NM_016320.4 | . |
| #5 | DEL | intron_variant | OTOGL | NM_173591.3 | . |
| #5 | INS | intron_variant | CAMKK2 | NM_001270485.1 | . |
| #5 | DEL | intron_variant | CCNA1 | NM_003914.3 | . |
| #5 | DEL | splice_region_variant&intron_variant | COG6 | NM_020751.2 | . |
| #5 | DEL | intron_variant | SOS2 | NM_006939.2 | . |
| #5 | DEL | intron_variant | TRIP11 | NM_004239.3 | . |
| #5 | DEL | splice_region_variant&intron_variant,intron_variant | TP53BP1,RNU6-28P | NM_001141980.1,NR_046489.1.2 | .,. |
| #5 | INS | intron_variant | CDH13 | NM_001220488.1 | . |
| #5 | DEL | 5_prime_UTR_variant,upstream_gene_variant,intergenic_region,transcript | RHOT1,RHOT1,LRRC37B-RHOT1,RHOT1 | NM_001033568.2,NM_001033568.2,LRRC37B-RHOT1,NM_001033568.2 | .,.,.,. |
| #5 | DEL | intron_variant | PSMD11 | NM_001270482.1 | . |
| #5 | DEL | upstream_gene_variant,intergenic_region | KLK1,KLK1-KLK15 | NM_002257.3,KLK1-KLK15 | .,. |
| #5 | DEL | intergenic_region | GCKR-C2orf16 | GCKR-C2orf16 | . |
| #5 | DEL | intergenic_region | SLC4A3-MIR4268 | SLC4A3-MIR4268 | . |
| #5 | DEL | splice_region_variant&intron_variant,upstream_gene_variant | CRELD2,ALG12 | NM_001135101.2,NM_024105.3 | .,. |
| #5 | DEL | intron_variant | KLF3 | NM_016531.5 | . |
| #5 | DEL | intron_variant | TBCK | NM_001163435.2 | . |
| #5 | DEL | 3_prime_UTR_variant | PLRG1 | NM_002669.3 | . |
| #5 | DEL | intergenic_region | IRX1-LOC101929153 | IRX1-LOC101929153 | . |
| #5 | INS | intron_variant | AGER | NM_001206929.1.2 | . |
| #5 | DEL | intron_variant | RNASET2 | NM_003730.4 | . |
| #5 | DEL | intergenic_region | SMOC2-THBS2 | SMOC2-THBS2 | . |
| #5 | DEL | intron_variant | IGFBP3 | NM_001013398.1 | . |
| #5 | INS | intron_variant | SNX16 | NM_022133.3 | . |
| #5 | DEL | intron_variant | LRRC69 | NM_001129890.1 | . |
| #5 | DEL | intron_variant | CSMD3 | NM_198123.1 | . |
| #5 | INS | intron_variant | ADAMTSL1 | NM_001040272.5 | . |
| #5 | DEL | 3_prime_UTR_variant | SYTL4 | NM_001129896.2 | . |
| #8 | DEL | intron_variant | HP1BP3 | NM_016287.3 | . |
| #8 | INS | 5_prime_UTR_variant | KCNK1 | NM_002245.3 | . |
| #8 | DEL | intron_variant | ANKRD26 | NM_014915.2 | . |
| #8 | DEL | upstream_gene_variant,upstream_gene_variant,intron_variant | HSPB2,HSPB2-C11orf52,CRYAB | NM_001541.3,NR_037651.1,NM_001289807.1 | .,.,. |
| #8 | INS | intron_variant | WIF1 | NM_007191.4 | . |
| #8 | INS | upstream_gene_variant,non_coding_exon_variant | TRHDE,TRHDE-AS1 | NM_013381.2,NR_026837.1 | .,. |
| #8 | DEL | 3_prime_UTR_variant,3_prime_UTR_variant | GLIPR1,KRR1 | NM_006851.2,NM_007043.6 | .,. |
| #8 | INS | intron_variant | GOLGA3 | NM_005895.3 | . |
| #8 | INS | intron_variant | LACC1 | NM_001128303.1 | . |
| #8 | DEL | intron_variant | FASN | NM_004104.4 | . |
| #8 | INS | 5_prime_UTR_variant | MAP4K4 | NM_145686.3 | . |
| #8 | INS | intragenic_variant | COL18A1 | COL18A1 | . |
| #8 | DEL | 3_prime_UTR_variant | PPIC | NM_000943.4 | . |
| #8 | DEL | intron_variant | ACAT2 | NM_005891.2 | . |
| #8 | DEL | intron_variant | NUGGC | NM_001010906.1 | . |
| #8 | DEL | intron_variant | HOOK3 | NM_032410.3 | . |
| #8 | DEL | intron_variant | KDM4C | NM_015061.3 | . |
| #8 | DEL | intron_variant | HP1BP3 | NM_016287.3 | . |
| #8 | INS | 5_prime_UTR_variant | KCNK1 | NM_002245.3 | . |
| #8 | INS | intron_variant | ANKRD26 | NM_014915.2 | . |
| #8 | DEL | intron_variant | ANKRD26 | NM_014915.2 | . |
| #8 | DEL | upstream_gene_variant,upstream_gene_variant,intron_variant | HSPB2,HSPB2-C11orf52,CRYAB | NM_001541.3,NR_037651.1,NM_001289807.1 | .,.,. |
| #8 | INS | intron_variant | WIF1 | NM_007191.4 | . |
| #8 | INS | upstream_gene_variant,non_coding_exon_variant | TRHDE,TRHDE-AS1 | NM_013381.2,NR_026837.1 | .,. |
| #8 | DEL | 3_prime_UTR_variant,3_prime_UTR_variant | GLIPR1,KRR1 | NM_006851.2,NM_007043.6 | .,. |
| #8 | INS | intron_variant | GOLGA3 | NM_005895.3 | . |
| #8 | INS | intron_variant | LACC1 | NM_001128303.1 | . |
| #8 | INS | intron_variant | KLF5 | NM_001730.4 | . |
| #8 | INS | intron_variant,intron_variant | MYHAS,MYH2 | NR_125367.1,NM_001100112.1 | .,. |
| #8 | DEL | intron_variant | FASN | NM_004104.4 | . |
| #8 | INS | 5_prime_UTR_variant | MAP4K4 | NM_145686.3 | . |
| #8 | DEL | intron_variant | SPEG | NM_005876.4 | . |
| #8 | INS | intron_variant | RPN2 | NM_002951.3 | . |
| #8 | INS | intragenic_variant | COL18A1 | COL18A1 | . |
| #8 | DEL | intron_variant | SLCO4C1 | NM_180991.4 | . |
| #8 | DEL | 3_prime_UTR_variant | PPIC | NM_000943.4 | . |
| #8 | DEL | disruptive_inframe_deletion | AK9 | NM_001145128.2 | p.Glu703del |
| #8 | DEL | intron_variant | ACAT2 | NM_005891.2 | . |
| #8 | DEL | intron_variant | NUGGC | NM_001010906.1 | . |
| #8 | DEL | intron_variant | HOOK3 | NM_032410.3 | . |
| #8 | DEL | intron_variant | KDM4C | NM_015061.3 | . |
| #9 | DEL | intron_variant | SDHC | NM_003001.3 | . |
| #9 | INS | intron_variant,intron_variant | RNU5F-1,EPRS | NR_002753.5,NM_004446.2 | .,. |
| #9 | INS | intron_variant | OVCH2 | NM_198185.4 | . |
| #9 | INS | intron_variant | DLG2 | NM_001142699.1 | . |
| #9 | INS | intron_variant | PLEKHA5 | NM_001256470.1 | . |
| #9 | DEL | intron_variant | KIF21A | NM_001173464.1 | . |
| #9 | INS | intron_variant | MPP5 | NM_022474.3 | . |
| #9 | DEL | intron_variant | HDC | NM_002112.3 | . |
| #9 | DEL | intron_variant,intron_variant | MIR548H4,NOX5 | NR_031680.1,NM_024505.3 | .,. |
| #9 | DEL | intron_variant | ZSCAN32 | NM_001284527.1 | . |
| #9 | DEL | intron_variant | NDE1 | NM_001143979.1 | . |
| #9 | DEL | splice_region_variant&intron_variant | RPGRIP1L | NM_015272.2 | . |
| #9 | DEL | upstream_gene_variant,intergenic_region | DPH1,RTN4RL1-DPH1 | NM_001383.3,RTN4RL1-DPH1 | .,. |
| #9 | INS | intron_variant | ADAMTS10 | NM_030957.3 | . |
| #9 | INS | 5_prime_UTR_variant | PTGIS | NM_000961.3 | . |
| #9 | DEL | inframe_deletion | TAF4 | NM_003185.3 | p.Pro186_Gly187del |
| #9 | DEL | upstream_gene_variant,intron_variant | ARFRP1,ZGPAT | NM_001267547.2,NM_032527.4 | .,. |
| #9 | DEL | intron_variant | SPICE1 | NM_144718.3 | . |
| #9 | INS | intron_variant,intron_variant | EEF1E1-BLOC1S5,EEF1E1 | NR_037618.1,NM_004280.4 | .,. |
| #9 | DEL | intron_variant | RFX6 | NM_173560.3 | . |
| #9 | DEL | intron_variant | NUP214 | NM_005085.3 | . |
| #9 | INS | intron_variant | BRWD3 | NM_153252.4 | . |
| #9 | INS | 3_prime_UTR_variant | SYTL4 | NM_001129896.2 | . |
| #9 | INS | intron_variant | SPANXN1 | NM_001009614.2 | . |
| #9 | DEL | intron_variant | ABCA4 | NM_000350.2 | . |
| #9 | DEL | intron_variant | SDHC | NM_003001.3 | . |
| #9 | INS | intron_variant,intron_variant | RNU5F-1,EPRS | NR_002753.5,NM_004446.2 | .,. |
| #9 | INS | intron_variant | TUBGCP2 | NM_001256617.1 | . |
| #9 | INS | intron_variant | OVCH2 | NM_198185.4 | . |
| #9 | INS | intron_variant | DLG2 | NM_001142699.1 | . |
| #9 | INS | intron_variant | PLEKHA5 | NM_001256470.1 | . |
| #9 | DEL | intron_variant | KIF21A | NM_001173464.1 | . |
| #9 | INS | intron_variant | MPP5 | NM_022474.3 | . |
| #9 | DEL | intron_variant,intron_variant | TTC7B,LOC101928909 | NM_001010854.1,NR_110134.1 | .,. |
| #9 | DEL | intron_variant | HDC | NM_002112.3 | . |
| #9 | DEL | intron_variant,intron_variant | MIR548H4,NOX5 | NR_031680.1,NM_024505.3 | .,. |
| #9 | INS | intron_variant | FAM169B | NM_182562.2 | . |
| #9 | DEL | intron_variant | ZSCAN32 | NM_001284527.1 | . |
| #9 | DEL | intron_variant | NDE1 | NM_001143979.1 | . |
| #9 | DEL | splice_region_variant&intron_variant | RPGRIP1L | NM_015272.2 | . |
| #9 | DEL | upstream_gene_variant,intergenic_region | DPH1,RTN4RL1-DPH1 | NM_001383.3,RTN4RL1-DPH1 | .,. |
| #9 | INS | intron_variant | JUP | NM_002230.2 | . |
| #9 | INS | intron_variant | ADAMTS10 | NM_030957.3 | . |
| #9 | INS | intron_variant | NEB | NM_001271208.1 | . |
| #9 | DEL | intron_variant | ICA1L | NM_001288622.1 | . |
| #9 | INS | 5_prime_UTR_variant | PTGIS | NM_000961.3 | . |
| #9 | DEL | inframe_deletion | TAF4 | NM_003185.3 | p.Pro186_Gly187del |
| #9 | DEL | 3_prime_UTR_variant | EEF1A2 | NM_001958.3 | . |
| #9 | DEL | upstream_gene_variant,intron_variant | ARFRP1,ZGPAT | NM_001267547.2,NM_032527.4 | .,. |
| #9 | DEL | intergenic_region | IGLL5-RTDR1 | IGLL5-RTDR1 | . |
| #9 | DEL | intron_variant | SPICE1 | NM_144718.3 | . |
| #9 | INS | intergenic_region | MIR4275-PCDH7 | MIR4275-PCDH7 | . |
| #9 | INS | intron_variant | CWC27 | NM_005869.3 | . |
| #9 | INS | intron_variant,intron_variant | EEF1E1-BLOC1S5,EEF1E1 | NR_037618.1,NM_004280.4 | .,. |
| #9 | DEL | intron_variant | RFX6 | NM_173560.3 | . |
| #9 | DEL | intron_variant | NUP214 | NM_005085.3 | . |
| #9 | INS | upstream_gene_variant,intergenic_region | PTCHD1,PTCHD1-AS-PTCHD1 | NM_173495.2,PTCHD1-AS-PTCHD1 | .,. |
| #9 | INS | intron_variant | BRWD3 | NM_153252.4 | . |
| #9 | INS | 3_prime_UTR_variant | SYTL4 | NM_001129896.2 | . |
| #9 | INS | intron_variant | SPANXN1 | NM_001009614.2 | . |

Supplementary Table S5 List of mutations (SNVs) in metastatic ovarian tumours.

|  | **Consequences** | **Gene** | **Transcript_id** | **HGVS_C** | **HGVS_P** |
| --- | --- | --- | --- | --- | --- |
| #5 | missense_variant | NBPF3 | NM_032264.4 | c.1332C>G | p.Asp444Glu |
| #5 | missense_variant | BAI2 | NM_001294335.1 | c.1474G>A | p.Asp492Asn |
| #5 | intron_variant | TTC18 | NM_145170.3 | c.1467-32T>G | . |
| #5 | missense_variant | CTBP2 | NM_022802.2 | c.2357A>G | p.Asn786Ser |
| #5 | intron_variant | ACAT1 | NM_000019.3 | c.941-36G>A | . |
| #5 | intergenic_region | GNG2-C14orf166 | GNG2-C14orf166 | . | . |
| #5 | upstream_gene_variant,non_coding_exon_variant | CCDC64B,LOC100128770 | NM_001103175.1,NR_047572.1 | c.-46G>A,n.1060C>T | .,. |
| #5 | missense_variant | USP6 | NM_004505.2 | c.398G>A | p.Arg133Lys |
| #5 | intron_variant | SSH2 | NM_001282129.1 | c.145-29A>T | . |
| #5 | intron_variant | ATAD5 | NM_024857.3 | c.4013-20G>T | . |
| #5 | synonymous_variant | CDC27 | NM_001114091.2 | c.501A>G | p.Thr167Thr |
| #5 | missense_variant | SERPINB3 | NM_006919.2 | c.967C>A | p.Leu323Ile |
| #5 | missense_variant | RGL3 | NM_001161616.2 | c.1549C>T | p.Arg517Trp |
| #5 | intron_variant,intron_variant | ZNF790-AS1,ZNF790 | NR_040027.1,NM_001242800.1 | n.449+36T>A,c.10-67A>T | .,. |
| #5 | synonymous_variant | ZNF540 | NM_001172225.2 | c.1227A>G | p.Lys409Lys |
| #5 | missense_variant | SIGLEC11 | NM_052884.2 | c.1720G>A | p.Ala574Thr |
| #5 | intergenic_region | FAM90A27P-BIRC8 | FAM90A27P-BIRC8 | . | . |
| #5 | synonymous_variant | LILRB1 | NM_001081637.2 | c.1344C>T | p.Thr448Thr |
| #5 | intron_variant,intron_variant | RNU6-81P,CCDC74A | NR_046946.1,NM_138770.2 | n.21-70026T>C,c.877-22T>C | .,. |
| #5 | missense_variant | TTN | NM_001267550.2 | c.17553G>T | p.Glu5851Asp |
| #5 | missense_variant | MAP2 | NM_002374.3 | c.3793G>T | p.Ala1265Ser |
| #5 | intergenic_region | ALPPL2-ALPI | ALPPL2-ALPI | . | . |
| #5 | intron_variant | COMMD7 | NM_053041.2 | c.428-21T>C | . |
| #5 | intron_variant | TOM1 | NM_001135732.1 | c.52+14G>T | . |
| #5 | upstream_gene_variant,intron_variant | BAIAP2L2,PLA2G6 | NM_025045.5,NM_003560.2 | c.-145T>C,c.2276+89T>C | .,. |
| #5 | synonymous_variant | MAGI1 | NM_001033057.1 | c.3867C>T | p.Pro1289Pro |
| #5 | missense_variant | ROBO1 | NM_002941.3 | c.4379C>T | p.Pro1460Leu |
| #5 | missense_variant | PDHA2 | NM_005390.4 | c.482G>A | p.Gly161Asp |
| #5 | intron_variant | MROH2B | NM_173489.4 | c.3749+43A>G | . |
| #5 | splice_donor_variant&intron_variant,intron_variant | NR2F1,MIR548AO | NM_005654.5,NR_049801.1 | c.991+1G>C,n.17-983G>C | .,. |
| #5 | missense_variant,intron_variant | FAM196B,DOCK2 | NM_001129891.1,NM_004946.2 | c.415T>C,c.2799+42632A>G | p.Ser139Pro,. |
| #5 | upstream_gene_variant,upstream_gene_variant,intron_variant | MIR1236,SKIV2L,NELFE | NR_031601.1.6,NM_006929.4,NM_002904.5 | n.-1G>A,c.-390C>T,c.75+57G>A | .,.,. |
| #5 | intron_variant | EIF3B | NM_001037283.1 | c.1810+9C>A | . |
| #5 | missense_variant | TMEM229A | NM_001136002.1 | c.928G>A | p.Val310Met |
| #5 | intron_variant | VIPR2 | NM_003382.4 | c.597+64C>T | . |
| #5 | intron_variant | XKR6 | NM_173683.3 | c.765-77446T>C | . |
| #5 | missense_variant,non_coding_exon_variant | SYP,SYP-AS1 | NM_003179.2,NR_046649.1 | c.58C>T,n.174G>A | p.Arg20Trp,. |
| #5 | synonymous_variant | HDX | NM_001177479.1 | c.408C>A | p.Ile136Ile |
| #5 | synonymous_variant | PCDH19 | NM_001184880.1 | c.1485G>A | p.Ser495Ser |
| #5 | splice_donor_variant&intron_variant | FLNA | NM_001110556.1 | c.987+1G>A | . |
| #5 | intron_variant | LRRIQ3 | NM_001105659.1 | c.573+1878C>T | . |
| #5 | missense_variant | NRAS | NM_002524.4 | c.182A>G | p.Gln61Arg |
| #5 | intron_variant | ADAMTSL4 | NM_001288608.1 | c.2832+12G>A | . |
| #5 | intron_variant | SPTA1 | NM_003126.2 | c.957+22G>A | . |
| #5 | missense_variant | CHIT1 | NM_003465.2 | c.802G>A | p.Gly268Arg |
| #5 | missense_variant | KLHDC8A | NM_001271863.1 | c.779G>A | p.Arg260Gln |
| #5 | missense_variant | TRAF3IP3 | NM_025228.3 | c.1127C>A | p.Ala376Asp |
| #5 | intron_variant | OBSCN | NM_001271223.2 | c.26150+39C>T | . |
| #5 | intergenic_region | LOC100129055-HSD17B7P2 | LOC100129055-HSD17B7P2 | . | . |
| #5 | synonymous_variant | RBP3 | NM_002900.2 | c.3546C>T | p.His1182His |
| #5 | missense_variant | HPSE2 | NM_021828.4 | c.881G>A | p.Arg294Gln |
| #5 | intron_variant | SORCS1 | NM_001013031.2 | c.960-68G>A | . |
| #5 | intron_variant | DNAJC24 | NM_181706.4 | c.320-18T>C | . |
| #5 | 5_prime_UTR_variant,upstream_gene_variant | YIF1A,TMEM151A | NM_020470.2,NM_153266.3 | c.-83C>G,c.-113G>C | .,. |
| #5 | missense_variant | SLCO2B1 | NM_007256.4 | c.1390G>T | p.Gly464Cys |
| #5 | missense_variant | C12orf5 | NM_020375.2 | c.198G>A | p.Met66Ile |
| #5 | intron_variant | NACA | NM_001113203.2 | c.1864+225C>T | . |
| #5 | 5_prime_UTR_variant | TSPAN8 | NM_004616.2 | c.-105G>A | . |
| #5 | missense_variant | CCDC64 | NM_207311.2 | c.865C>T | p.Arg289Trp |
| #5 | missense_variant | HTR2A | NM_000621.4 | c.712G>A | p.Gly238Ser |
| #5 | synonymous_variant | PCDH8 | NM_002590.3 | c.927C>T | p.Ala309Ala |
| #5 | synonymous_variant | IRG1 | NM_001258406.1 | c.642G>T | p.Gly214Gly |
| #5 | intron_variant | FRMD6 | NM_001267046.1 | c.1025-44G>A | . |
| #5 | missense_variant | MAGEL2 | NM_019066.4 | c.733C>A | p.Pro245Thr |
| #5 | intron_variant | GLDN | NM_181789.2 | c.817+36G>T | . |
| #5 | splice_region_variant&intron_variant | NARFL | NM_022493.1 | c.693+5C>T | . |
| #5 | intron_variant | CACNA1H | NM_021098.2 | c.4477-27G>A | . |
| #5 | missense_variant | GPR114 | NM_153837.1 | c.1334C>T | p.Ala445Val |
| #5 | upstream_gene_variant,intron_variant | LOC102724009,KIF1C | NR_120665.1,NM_006612.5 | n.-1G>A,c.1751-31C>T | .,. |
| #5 | missense_variant | TP53 | NM_000546.5 | c.413C>T | p.Ala138Val |
| #5 | intron_variant | DNAH2 | NM_020877.2 | c.1171-748T>C | . |
| #5 | missense_variant | CDC27 | NM_001114091.2 | c.794G>A | p.Gly265Asp |
| #5 | intron_variant | GRB2 | NM_002086.4 | c.78+23A>G | . |
| #5 | intron_variant | MYOM1 | NM_003803.3 | c.4378+17G>A | . |
| #5 | missense_variant | ROCK1 | NM_005406.2 | c.162C>A | p.Asn54Lys |
| #5 | missense_variant | NOL4 | NM_003787.4 | c.356C>T | p.Thr119Met |
| #5 | missense_variant | RHPN2 | NM_033103.4 | c.217G>A | p.Val73Met |
| #5 | synonymous_variant | ZNF45 | NM_003425.3 | c.891T>C | p.Val297Val |
| #5 | intron_variant | PVRL2 | NM_001042724.1 | c.1043-3608C>T | . |
| #5 | synonymous_variant | MYT1L | NM_015025.2 | c.2955C>T | p.Asp985Asp |
| #5 | missense_variant | FER1L5 | NM_001293083.1 | c.2663G>A | p.Arg888Gln |
| #5 | missense_variant | KCNH7 | NM_033272.3 | c.672A>T | p.Lys224Asn |
| #5 | splice_acceptor_variant&intron_variant,intron_variant | TTN,MIR548N | NM_001267550.2,NR_031666.1 | c.34856-1G>A,n.49-3770C>T | .,. |
| #5 | missense_variant | ASNSD1 | NM_019048.2 | c.1261C>A | p.Pro421Thr |
| #5 | synonymous_variant | SMARCAL1 | NM_001127207.1 | c.1440G>A | p.Pro480Pro |
| #5 | synonymous_variant | AQP12A | NM_198998.2 | c.249T>C | p.Thr83Thr |
| #5 | intron_variant | RALGAPA2 | NM_020343.3 | c.5053-42C>T | . |
| #5 | missense_variant | XKR7 | NM_001011718.1 | c.961G>A | p.Ala321Thr |
| #5 | missense_variant | TOX2 | NM_001098797.1 | c.1244C>G | p.Ala415Gly |
| #5 | intron_variant | SLC12A5 | NM_001134771.1 | c.122-5538C>T | . |
| #5 | missense_variant | CSE1L | NM_001316.3 | c.211A>G | p.Lys71Glu |
| #5 | synonymous_variant | ATP9A | NM_006045.1 | c.2070T>C | p.Asn690Asn |
| #5 | missense_variant | TPTE | NM_199261.3 | c.479T>A | p.Ile160Asn |
| #5 | missense_variant | SLC7A4 | NM_004173.2 | c.1354G>A | p.Val452Ile |
| #5 | missense_variant | PIWIL3 | NM_001008496.3 | c.728G>A | p.Arg243His |
| #5 | 3_prime_UTR_variant | RRP7A | NM_015703.4 | c.*19A>G | . |
| #5 | missense_variant | TCAIM | NM_001282913.1 | c.326G>C | p.Arg109Pro |
| #5 | upstream_gene_variant,intron_variant | MIR711,COL7A1 | NR_031756.1,NM_000094.3 | n.-1C>T,c.4819-46C>T | .,. |
| #5 | missense_variant,upstream_gene_variant | COL7A1,MIR711 | NM_000094.3,NR_031756.1 | c.4489C>T,n.-1C>T | p.Arg1497Cys,. |
| #5 | synonymous_variant,upstream_gene_variant,intron_variant | NAT6,IFRD2,HYAL3 | NM_012191.3,NM_006764.4,NM_001200029.1 | c.750T>A,c.-130T>A,c.-17-1161T>A | p.Thr250Thr,.,. |
| #5 | stop_gained | DOCK3 | NM_004947.4 | c.376C>T | p.Arg126* |
| #5 | splice_region_variant&intron_variant | FAM19A1 | NM_001252216.1 | c.259+8C>A | . |
| #5 | missense_variant | RGS12 | NM_198229.2 | c.3458G>T | p.Gly1153Val |
| #5 | synonymous_variant | EVC2 | NM_147127.4 | c.2415C>T | p.Ser805Ser |
| #5 | missense_variant&splice_region_variant | CPZ | NM_001014447.2 | c.1606C>A | p.Pro536Thr |
| #5 | synonymous_variant | HERC6 | NM_017912.3 | c.165G>A | p.Leu55Leu |
| #5 | intron_variant | OXCT1 | NM_000436.3 | c.955+26G>T | . |
| #5 | intron_variant | GPR98 | NM_032119.3 | c.8904-42T>A | . |
| #5 | stop_gained | APC | NM_000038.5 | c.904C>T | p.Arg302* |
| #5 | stop_gained | APC | NM_000038.5 | c.4033G>T | p.Glu1345* |
| #5 | missense_variant,upstream_gene_variant | PROB1,MZB1 | NM_001161546.1,NM_016459.3 | c.1088C>T,c.-61C>T | p.Pro363Leu,. |
| #5 | missense_variant | GNPDA1 | NM_005471.4 | c.560C>T | p.Thr187Met |
| #5 | 3_prime_UTR_variant,upstream_gene_variant | F12,PFN3 | NM_000505.3,NM_001029886.2 | c.*95G>A,c.-61G>A | .,. |
| #5 | missense_variant | EHMT2 | NM_001289413.1.3 | c.94C>G | p.Pro32Ala |
| #5 | intron_variant | PRIM2 | NM_000947.4 | c.834+16T>C | . |
| #5 | intron_variant | AHI1 | NM_001134830.1 | c.135+522C>A | . |
| #5 | non_coding_exon_variant | NCF1B | NR_003186.1 | n.491C>G | . |
| #5 | missense_variant | CLDN4 | NM_001305.4 | c.433C>A | p.Gln145Lys |
| #5 | synonymous_variant | POM121C | NM_001099415.2 | c.2346C>G | p.Ser782Ser |
| #5 | stop_gained | PEX1 | NM_000466.2 | c.547C>T | p.Arg183* |
| #5 | missense_variant | KPNA7 | NM_001145715.1 | c.1381G>A | p.Asp461Asn |
| #5 | intron_variant | LAMTOR4 | NM_001008395.2 | c.203-153A>G | . |
| #5 | missense_variant | FOXP2 | NM_148898.3 | c.1139G>A | p.Ser380Asn |
| #5 | missense_variant | KEL | NM_000420.2 | c.2024G>A | p.Arg675Gln |
| #5 | synonymous_variant | DLGAP2 | NM_004745.4 | c.96C>T | p.His32His |
| #5 | missense_variant | PPP1R3B | NM_001201329.1 | c.701G>A | p.Arg234Gln |
| #5 | synonymous_variant | BLK | NM_001715.2 | c.936C>T | p.Thr312Thr |
| #5 | missense_variant | UNC5D | NM_080872.2 | c.901T>G | p.Cys301Gly |
| #5 | intergenic_region | C8orf22-SNTG1 | C8orf22-SNTG1 | . | . |
| #5 | missense_variant | TNFRSF11B | NM_002546.3 | c.877G>A | p.Glu293Lys |
| #5 | synonymous_variant | BAI1 | NM_001702.2 | c.3093C>T | p.Thr1031Thr |
| #5 | synonymous_variant | SH3GL2 | NM_003026.3 | c.537G>A | p.Pro179Pro |
| #5 | intergenic_region | CNTNAP3B-LOC101927827 | CNTNAP3B-LOC101927827 | . | . |
| #5 | missense_variant | DAPK1 | NM_001288729.1 | c.3686G>A | p.Gly1229Glu |
| #5 | missense_variant | LAMC3 | NM_006059.3 | c.3896C>T | p.Ala1299Val |
| #5 | 5_prime_UTR_variant | PIGA | NM_002641.3 | c.-89G>A | . |
| #5 | missense_variant | CXorf23 | NM_198279.3 | c.554C>G | p.Ser185Cys |
| #5 | synonymous_variant | CXorf22 | NM_152632.3 | c.1386C>A | p.Gly462Gly |
| #5 | 5_prime_UTR_variant | FAM47C | NM_001013736.2 | c.-36G>A | . |
| #5 | splice_region_variant&intron_variant | ZNF182 | NM_001178099.1 | c.289+8G>A | . |
| #5 | synonymous_variant | HUWE1 | NM_031407.6 | c.10248C>T | p.Gly3416Gly |
| #5 | intron_variant | AR | NM_000044.3 | c.1769-5150T>C | . |
| #5 | intron_variant,intron_variant | BCYRN1,ZMYM3 | NR_001568.1,NM_005096.3 | n.173-30996G>A,c.3920+28G>A | .,. |
| #5 | missense_variant | PGK1 | NM_000291.3 | c.910G>T | p.Ala304Ser |
| #5 | missense_variant | IL13RA2 | NM_000640.2 | c.743G>A | p.Arg248Gln |
| #5 | intron_variant | MAP7D3 | NM_024597.3 | c.2140-18T>C | . |
| #8 | intron_variant | ARHGEF16 | NM_014448.3 | c.1473+13G>A | . |
| #8 | non_coding_exon_variant | ESPNP | NR_026567.1 | n.813C>T | . |
| #8 | missense_variant | IGSF21 | NM_032880.4 | c.872G>A | p.Arg291His |
| #8 | intron_variant | USP48 | NM_032236.5 | c.666-17G>A | . |
| #8 | intron_variant | COL16A1 | NM_001856.3 | c.3880-87G>T | . |
| #8 | synonymous_variant | AK5 | NM_174858.2 | c.1596C>T | p.Tyr532Tyr |
| #8 | intron_variant | PLEKHA6 | NM_014935.4 | c.1524+1737G>A | . |
| #8 | synonymous_variant | FMN2 | NM_020066.4 | c.2880C>G | p.Pro960Pro |
| #8 | missense_variant | MRGPRE | NM_001039165.2 | c.275C>T | p.Pro92Leu |
| #8 | stop_gained | CCDC73 | NM_001008391.3 | c.1588G>T | p.Glu530* |
| #8 | missense_variant | SLC22A20 | NM_001004326.4 | c.422C>T | p.Ala141Val |
| #8 | missense_variant | KAT5 | NM_182710.2 | c.322G>T | p.Ala108Ser |
| #8 | synonymous_variant | MOGAT2 | NM_025098.2 | c.492A>G | p.Glu164Glu |
| #8 | missense_variant | GUCY2C | NM_004963.3 | c.203G>A | p.Arg68His |
| #8 | initiator_codon_variant | PYROXD1 | NM_024854.3 | c.1A>T | p.Met1? |
| #8 | missense_variant | SYT10 | NM_198992.3 | c.1057G>T | p.Asp353Tyr |
| #8 | intron_variant | PPHLN1 | NM_016488.6 | c.72+7847G>A | . |
| #8 | missense_variant | GJA3 | NM_021954.3 | c.1031C>T | p.Pro344Leu |
| #8 | missense_variant | FRY | NM_023037.2 | c.4268C>T | p.Thr1423Met |
| #8 | missense_variant | FREM2 | NM_207361.5 | c.6839G>A | p.Gly2280Glu |
| #8 | splice_region_variant&intron_variant | CKAP2 | NM_001098525.2 | c.235-7C>A | . |
| #8 | intron_variant | KLHL1 | NM_020866.2 | c.1227+15C>T | . |
| #8 | intergenic_region | OR4N5-OR11G2 | OR4N5-OR11G2 | . | . |
| #8 | intergenic_region | OR4E2-DAD1 | OR4E2-DAD1 | . | . |
| #8 | synonymous_variant,intron_variant | CKMT1B,RNU6-28P | NM_020990.3,NR_046489.1.2 | c.1056A>G,n.35-3718A>G | p.Lys352Lys,. |
| #8 | synonymous_variant,intron_variant | CKMT1B,RNU6-28P | NM_020990.3,NR_046489.1.2 | c.1086T>C,n.35-3688T>C | p.Ala362Ala,. |
| #8 | missense_variant,upstream_gene_variant | DUOX2,DUOXA2 | NM_014080.4,NM_207581.3 | c.123G>T,c.-282C>A | p.Trp41Cys,. |
| #8 | stop_gained | FBN1 | NM_000138.4 | c.3973G>T | p.Glu1325* |
| #8 | intragenic_variant | NEDD4 | NEDD4 | . | . |
| #8 | intron_variant | GLYR1 | NM_032569.3 | c.732+9G>T | . |
| #8 | missense_variant,upstream_gene_variant | ARL6IP1,RPS15A | NM_015161.1,NM_001019.4 | c.571A>T,c.-97A>T | p.Ile191Leu,. |
| #8 | intergenic_region | UBE2MP1-LOC283914 | UBE2MP1-LOC283914 | . | . |
| #8 | missense_variant | DHX38 | NM_014003.3 | c.3451G>A | p.Val1151Met |
| #8 | synonymous_variant | KRTAP9-4 | NM_033191.2 | c.384C>G | p.Pro128Pro |
| #8 | missense_variant | CNP | NM_033133.4 | c.464G>A | p.Arg155Gln |
| #8 | intron_variant | ANKFN1 | NM_153228.2 | c.1109-75G>A | . |
| #8 | missense_variant,intron_variant | MARCH10,MIR548W | NM_001288779.1,NR_036146.1 | c.1735G>A,n.21+14700C>T | p.Val579Ile,. |
| #8 | missense_variant | TJP3 | NM_001267561.1 | c.866C>T | p.Pro289Leu |
| #8 | intron_variant | CREB3L3 | NM_032607.2 | c.891-9C>T | . |
| #8 | missense_variant,intron_variant | SIGLEC10,LOC100129083 | NM_033130.4,NM_001256795.1 | c.430C>A,c.317-190G>T | p.Gln144Lys,. |
| #8 | missense_variant | ARHGEF33 | NM_001145451.2 | c.427C>T | p.Arg143Cys |
| #8 | intron_variant | EN1 | NM_001426.3 | c.863-30A>C | . |
| #8 | missense_variant | MARCO | NM_006770.3 | c.575C>T | p.Ser192Leu |
| #8 | missense_variant | KCNH7 | NM_033272.3 | c.1916C>T | p.Ser639Leu |
| #8 | synonymous_variant | TMEFF2 | NM_016192.2 | c.636G>A | p.Ser212Ser |
| #8 | synonymous_variant | PRKAG3 | NM_017431.2 | c.666C>T | p.Asn222Asn |
| #8 | missense_variant | TWIST2 | NM_001271893.3 | c.242C>T | p.Ser81Leu |
| #8 | intergenic_region | C2orf54-LOC200772 | C2orf54-LOC200772 | . | . |
| #8 | intron_variant | EPB41L1 | NM_012156.2 | c.447+84G>A | . |
| #8 | missense_variant | APCDD1L | NM_153360.1 | c.686C>T | p.Ala229Val |
| #8 | missense_variant | CHRNA4 | NM_000744.6 | c.1109G>A | p.Arg370Gln |
| #8 | synonymous_variant | GORASP1 | NM_031899.3 | c.585C>T | p.Gly195Gly |
| #8 | synonymous_variant,upstream_gene_variant | QARS,MIR6890 | NM_005051.2,NR_106950.1 | c.840T>C,n.-1T>C | p.His280His,. |
| #8 | missense_variant | CACNA1D | NM_000720.3 | c.916T>C | p.Ser306Pro |
| #8 | missense_variant | FEZF2 | NM_018008.3 | c.443C>T | p.Ala148Val |
| #8 | missense_variant,intron_variant | THOC7,C3orf49 | NM_025075.3,NR_026866.1 | c.38G>A,n.957-5268C>T | p.Arg13His,. |
| #8 | stop_gained | TMEM108 | NM_001136469.2 | c.1317G>A | p.Trp439* |
| #8 | synonymous_variant | SLC9A9 | NM_173653.3 | c.918T>C | p.Cys306Cys |
| #8 | missense_variant | DDX60L | NM_001012967.2 | c.4259C>T | p.Ala1420Val |
| #8 | intron_variant | KLKB1 | NM_000892.3 | c.1145-19G>A | . |
| #8 | stop_gained | DNAH5 | NM_001369.2 | c.5748G>A | p.Trp1916* |
| #8 | synonymous_variant | DNAH5 | NM_001369.2 | c.5565C>A | p.Ile1855Ile |
| #8 | synonymous_variant | AGXT2 | NM_031900.3 | c.258G>T | p.Leu86Leu |
| #8 | missense_variant | NIPBL | NM_133433.3 | c.3602G>A | p.Arg1201Lys |
| #8 | missense_variant | RNF180 | NM_001113561.1 | c.452C>T | p.Ala151Val |
| #8 | splice_region_variant&intron_variant | GEMIN5 | NM_015465.4 | c.1294-3T>G | . |
| #8 | stop_gained | GABRG2 | NM_198903.2 | c.1417C>T | p.Arg473* |
| #8 | synonymous_variant | UIMC1 | NM_001199297.1 | c.1344C>A | p.Thr448Thr |
| #8 | synonymous_variant | RREB1 | NM_001003699.3 | c.2190C>A | p.Ile730Ile |
| #8 | missense_variant | DSP | NM_004415.2 | c.5618G>A | p.Arg1873His |
| #8 | stop_gained | SLC17A4 | NM_005495.2 | c.106C>T | p.Arg36* |
| #8 | missense_variant | TBC1D22B | NM_017772.2 | c.1067G>A | p.Ser356Asn |
| #8 | intron_variant | PTCRA | NM_001243168.1 | c.58+125C>T | . |
| #8 | intron_variant | PTCRA | NM_001243168.1 | c.59-635C>T | . |
| #8 | intron_variant | PRIM2 | NM_000947.4 | c.1021-33595G>C | . |
| #8 | missense_variant | SLC2A12 | NM_145176.2 | c.722G>T | p.Arg241Ile |
| #8 | missense_variant | WIPI2 | NM_015610.3 | c.770G>T | p.Ser257Ile |
| #8 | synonymous_variant | BMPER | NM_133468.4 | c.24G>A | p.Gly8Gly |
| #8 | non_coding_exon_variant | CDC14C | NR_003595.1 | n.771A>T | . |
| #8 | synonymous_variant | DTX2 | NM_001102594.1 | c.792C>G | p.Thr264Thr |
| #8 | missense_variant | CPA4 | NM_016352.3 | c.887A>G | p.Gln296Arg |
| #8 | missense_variant,upstream_gene_variant | CRYGN,MIR3907 | NM_144727.1,NR_037468.1 | c.122C>T,n.-1C>T | p.Ser41Phe,. |
| #8 | intron_variant | FGFR1 | NM_001174067.1 | c.1029+66A>G | . |
| #8 | missense_variant | PCMTD1 | NM_052937.3 | c.743G>A | p.Arg248His |
| #8 | synonymous_variant | RP1 | NM_006269.1 | c.4926C>T | p.Ile1642Ile |
| #8 | intron_variant | FRMPD1 | NM_014907.2 | c.613-30G>A | . |
| #8 | intergenic_region | ANKRD20A3-LOC642236 | ANKRD20A3-LOC642236 | . | . |
| #8 | synonymous_variant | RASEF | NM_152573.3 | c.786C>T | p.Arg262Arg |
| #8 | intron_variant | IARS | NM_002161.5 | c.2308-406T>G | . |
| #8 | intron_variant | CACNA1B | NM_000718.3 | c.5222+12G>A | . |
| #8 | intron_variant | PHF8 | NM_001184896.1 | c.2017+17T>G | . |
| #8 | missense_variant | KIAA1210 | NM_020721.1 | c.929G>A | p.Arg310Gln |
| #8 | missense_variant | GPR101 | NM_054021.1 | c.292C>A | p.Pro98Thr |
| #8 | missense_variant | MTMR1 | NM_003828.2 | c.1909C>T | p.Arg637Trp |
| #8 | missense_variant | ATAD3B | NM_031921.4 | c.1798A>G | p.Thr600Ala |
| #8 | missense_variant | ATAD3B | NM_031921.4 | c.1810T>C | p.Tyr604His |
| #8 | synonymous_variant | HSPG2 | NM_001291860.1 | c.4932C>T | p.Pro1644Pro |
| #8 | synonymous_variant | HIVEP3 | NM_024503.4 | c.5526G>T | p.Val1842Val |
| #8 | missense_variant | LRRIQ3 | NM_001105659.1 | c.1205G>A | p.Arg402Gln |
| #8 | missense_variant | ADAMTSL4 | NM_001288608.1 | c.2378C>T | p.Pro793Leu |
| #8 | intron_variant | SMG5 | NM_015327.2 | c.1117+11C>G | . |
| #8 | missense_variant | PRCC | NM_005973.4 | c.370C>A | p.Pro124Thr |
| #8 | stop_gained | CACNA1E | NM_001205293.1 | c.6121C>T | p.Arg2041* |
| #8 | synonymous_variant | IRF6 | NM_006147.3 | c.135G>A | p.Arg45Arg |
| #8 | missense_variant | CAPN2 | NM_001748.4 | c.700A>G | p.Lys234Glu |
| #8 | synonymous_variant | PCDH15 | NM_001142763.1 | c.4326G>A | p.Pro1442Pro |
| #8 | synonymous_variant,upstream_gene_variant | KCNMA1,LOC101929328 | NM_001161352.1,NR_120655.1 | c.3480G>A,n.-1C>T | p.Pro1160Pro,. |
| #8 | missense_variant | DRD4 | NM_000797.3 | c.652G>A | p.Gly218Ser |
| #8 | missense_variant | ART1 | NM_004314.2 | c.403G>A | p.Val135Met |
| #8 | stop_gained | TTC17 | NM_018259.5 | c.1633G>T | p.Glu545* |
| #8 | splice_region_variant&intron_variant | FNBP4 | NM_015308.2 | c.1820+6C>T | . |
| #8 | missense_variant | FAM111A | NM_001142519.1 | c.129G>A | p.Met43Ile |
| #8 | missense_variant | ZBTB3 | NM_024784.3 | c.992C>A | p.Ala331Asp |
| #8 | missense_variant,upstream_gene_variant | ESRRA,PRDX5 | NM_001282450.1,NM_012094.4 | c.1055G>C,c.-129G>C | p.Arg352Pro,. |
| #8 | missense_variant | MYO7A | NM_000260.3 | c.1261C>T | p.Pro421Ser |
| #8 | splice_region_variant&intron_variant | CNTN5 | NM_001243270.1 | c.980+4A>G | . |
| #8 | intron_variant | KDM5A | NM_001042603.2 | c.4867-389G>A | . |
| #8 | intron_variant | LEPREL2 | NM_014262.4 | c.1263-17C>T | . |
| #8 | stop_gained | DUSP16 | NM_030640.2 | c.1447C>T | p.Arg483* |
| #8 | intron_variant | MUC19 | NM_173600.2 | c.23989+13C>T | . |
| #8 | synonymous_variant | FAM186A | NM_001145475.1 | c.3513T>C | p.Leu1171Leu |
| #8 | missense_variant,intron_variant | RFX4,LOC100287944 | NM_001206691.1,NR_040246.1 | c.1537G>A,n.142+41728C>T | p.Ala513Thr,. |
| #8 | missense_variant | LATS2 | NM_014572.2 | c.1741C>T | p.Arg581Cys |
| #8 | missense_variant | PCDH20 | NM_022843.3 | c.245T>A | p.Ile82Asn |
| #8 | intergenic_region | WHAMMP2-LOC100289656 | WHAMMP2-LOC100289656 | . | . |
| #8 | missense_variant,intron_variant | MAP1A,RNU6-28P | NM_002373.5,NR_046489.1.2 | c.2411C>T,n.35-78705C>T | p.Thr804Met,. |
| #8 | splice_region_variant&intron_variant | RSL24D1 | NM_016304.2 | c.419-4A>T | . |
| #8 | synonymous_variant | UACA | NM_018003.2 | c.2772C>T | p.Ser924Ser |
| #8 | missense_variant | RBFOX1 | NM_145891.2 | c.541G>A | p.Val181Ile |
| #8 | intron_variant | SNX29P1 | NR_045011.1 | n.107+33A>G | . |
| #8 | intron_variant | KATNB1 | NM_005886.2 | c.1177+31G>A | . |
| #8 | intron_variant | HYDIN | NM_001270974.1 | c.6532-62G>T | . |
| #8 | missense_variant | PHLPP2 | NM_015020.3 | c.629G>A | p.Arg210Gln |
| #8 | 3_prime_UTR_variant | YWHAE | NM_006761.4 | c.*57T>C | . |
| #8 | missense_variant | SGSM2 | NM_014853.2 | c.2021G>A | p.Arg674His |
| #8 | stop_gained | TP53 | NM_000546.5 | c.637C>T | p.Arg213* |
| #8 | missense_variant | TBC1D26 | NM_178571.4 | c.206G>A | p.Arg69His |
| #8 | intron_variant | TOP3A | NM_004618.3 | c.391-9G>A | . |
| #8 | missense_variant,intron_variant | SGK494,SPAG5-AS1 | NM_001174103.1,NR_040012.1 | c.244G>A,n.273-2765C>T | p.Glu82Lys,. |
| #8 | intron_variant | ACSF2 | NM_001288968.1 | c.529-11G>A | . |
| #8 | intergenic_region | ANKFN1-NOG | ANKFN1-NOG | . | . |
| #8 | intron_variant,intron_variant | LOC101927688,SEPT4 | NR_110810.1,NM_001256782.1 | n.258+1317G>T,c.106-156C>A | .,. |
| #8 | synonymous_variant | BAIAP2 | NM_017451.2 | c.1365G>A | p.Thr455Thr |
| #8 | splice_region_variant&intron_variant | FASN | NM_004104.4 | c.778+6C>T | . |
| #8 | non_coding_exon_variant | LINC00470 | NR_023925.1 | n.379C>T | . |
| #8 | synonymous_variant | ABHD17A | NM_031213.3 | c.513T>C | p.Asn171Asn |
| #8 | synonymous_variant | CD209 | NM_021155.3 | c.1098C>T | p.Asp366Asp |
| #8 | missense_variant | MUC16 | NM_024690.2 | c.874G>A | p.Asp292Asn |
| #8 | missense_variant&splice_region_variant | MRPL4 | NM_015956.2 | c.555C>A | p.Asp185Glu |
| #8 | intron_variant | MAST3 | NM_015016.1 | c.2118+48A>G | . |
| #8 | synonymous_variant,upstream_gene_variant | OVOL3,TBCB | NM_001270948.1,NM_001281.2 | c.111G>A,c.-576G>A | p.Val37Val,. |
| #8 | missense_variant | CAPN12 | NM_144691.3 | c.412G>A | p.Val138Ile |
| #8 | intron_variant | XRCC1 | NM_006297.2 | c.1621+16A>G | . |
| #8 | synonymous_variant | CCDC114 | NM_144577.3 | c.1086C>T | p.Ala362Ala |
| #8 | missense_variant | KLK13 | NM_015596.1 | c.788G>T | p.Arg263Leu |
| #8 | synonymous_variant | ZNF865 | NM_001195605.1 | c.2025C>T | p.Gly675Gly |
| #8 | missense_variant | APOB | NM_000384.2 | c.9491C>T | p.Thr3164Met |
| #8 | intron_variant | DRC1 | NM_145038.3 | c.1510-49G>A | . |
| #8 | missense_variant | EML6 | NM_001039753.2 | c.5677G>A | p.Ala1893Thr |
| #8 | intron_variant | VWA3B | NM_144992.4 | c.2674-4762A>T | . |
| #8 | synonymous_variant | GLI2 | NM_005270.4 | c.4260G>A | p.Ala1420Ala |
| #8 | intron_variant,intron_variant | RNU6-81P,CCDC74A | NR_046946.1,NM_138770.2 | n.21-70026T>C,c.877-22T>C | .,. |
| #8 | stop_gained | THSD7B | NM_001080427.1 | c.4341C>A | p.Cys1447* |
| #8 | synonymous_variant | CHRND | NM_000751.2 | c.663C>T | p.Asn221Asn |
| #8 | intron_variant | PIGU | NM_080476.4 | c.627+90T>A | . |
| #8 | synonymous_variant | CHD6 | NM_032221.4 | c.2493G>A | p.Gly831Gly |
| #8 | missense_variant | ZNF217 | NM_006526.2 | c.2885C>T | p.Pro962Leu |
| #8 | missense_variant | ZNF831 | NM_178457.2 | c.3215G>A | p.Ser1072Asn |
| #8 | missense_variant,upstream_gene_variant | CRELD2,ALG12 | NM_001135101.2,NM_024105.3 | c.236T>C,c.-275A>G | p.Leu79Pro,. |
| #8 | intron_variant | ZNF385D | NM_024697.2 | c.277-747C>A | . |
| #8 | upstream_gene_variant,intron_variant | RNF123,MST1 | NM_022064.3,NM_020998.3 | c.-127A>G,c.1251-9T>C | .,. |
| #8 | splice_acceptor_variant&intron_variant | SLC9A9 | NM_173653.3 | c.1470-2A>G | . |
| #8 | missense_variant | TTC14 | NM_133462.3 | c.364G>A | p.Asp122Asn |
| #8 | missense_variant | MAP3K13 | NM_001242314.1 | c.248G>A | p.Ser83Asn |
| #8 | missense_variant | CCNA2 | NM_001237.3 | c.1009G>A | p.Gly337Arg |
| #8 | intron_variant | RNF150 | NM_020724.1 | c.987+52C>A | . |
| #8 | missense_variant | DNAH5 | NM_001369.2 | c.6715C>A | p.Pro2239Thr |
| #8 | missense_variant | ANKRD31 | NM_001164443.1 | c.1781A>G | p.Glu594Gly |
| #8 | intron_variant | ZFYVE16 | NM_001105251.2 | c.70+23C>T | . |
| #8 | missense_variant,upstream_gene_variant,intron_variant,intron_variant | PCDHA3,PCDHA4,PCDHA1,PCDHA2 | NM_018906.2,NM_018907.3,NM_018900.3,NM_018905.2 | c.2104G>A,c.-115G>A,c.2394+14617G>A,c.2388+5949G>A | p.Val702Ile,.,.,. |
| #8 | splice_region_variant&intron_variant | CDHR2 | NM_001171976.1 | c.622-4G>A | . |
| #8 | intron_variant | GRM6 | NM_000843.3 | c.1355-426C>T | . |
| #8 | intron_variant | GCNT2 | NM_145649.4 | c.925+26774A>G | . |
| #8 | synonymous_variant | SOX4 | NM_003107.2 | c.1011C>T | p.Pro337Pro |
| #8 | missense_variant | MUC22 | NM_001198815.1.7 | c.4088C>A | p.Ser1363Tyr |
| #8 | 5_prime_UTR_premature_start_codon_gain_variant,5_prime_UTR_variant | BRD2,BRD2 | NM_001199455.1.5,NM_001199455.1.5 | c.-799G>T,c.-799G>T | .,. |
| #8 | missense_variant | EFHC1 | NM_018100.3 | c.1055G>A | p.Arg352Gln |
| #8 | missense_variant | KBTBD2 | NM_015483.2 | c.755C>T | p.Ser252Phe |
| #8 | missense_variant | COBL | NM_001287436.1 | c.353T>A | p.Phe118Tyr |
| #8 | 3_prime_UTR_variant,non_coding_exon_variant | POM121,NSUN5P2 | NM_001257190.2,NR_033323.3 | c.*1439C>G,n.868G>C | .,. |
| #8 | stop_lost | GTF2IRD1 | NM_001199207.1 | c.2931G>T | p.Ter977Tyrext*? |
| #8 | intron_variant | ATXN7L1 | NM_020725.1 | c.1517+2181C>A | . |
| #8 | intron_variant | CNTNAP2 | NM_014141.5 | c.1083+16C>T | . |
| #8 | missense_variant | SSPO | NM_198455.2 | c.4304G>C | p.Cys1435Ser |
| #8 | missense_variant | LZTS1 | NM_021020.3 | c.107G>A | p.Arg36Gln |
| #8 | missense_variant | HR | NM_005144.4 | c.1784G>A | p.Gly595Asp |
| #8 | 5_prime_UTR_variant | LOXL2 | NM_002318.2 | c.-60C>A | . |
| #8 | intron_variant | ELP3 | NM_018091.5 | c.1192-10T>A | . |
| #8 | synonymous_variant | PXDNL | NM_144651.4 | c.3252G>A | p.Ala1084Ala |
| #8 | missense_variant | PTPRD | NM_002839.3 | c.653C>A | p.Ser218Tyr |
| #8 | missense_variant | BNC2 | NM_017637.5 | c.3233G>A | p.Arg1078Gln |
| #8 | missense_variant | SPATA31D1 | NM_001001670.2 | c.4588A>C | p.Thr1530Pro |
| #8 | intergenic_region | LINC00475-IARS | LINC00475-IARS | . | . |
| #8 | synonymous_variant | ABCA1 | NM_005502.3 | c.3834G>A | p.Lys1278Lys |
| #8 | missense_variant | CDK5RAP2 | NM_018249.5 | c.1643C>T | p.Ser548Leu |
| #8 | intron_variant | C5 | NM_001735.2 | c.4398+43C>A | . |
| #8 | missense_variant | GSN | NM_000177.4 | c.1942A>G | p.Lys648Glu |
| #8 | intron_variant | MAPKAP1 | NM_001006617.1 | c.1346-2785A>G | . |
| #8 | synonymous_variant | LCN9 | NM_001001676.1 | c.66C>T | p.Thr22Thr |
| #8 | missense_variant&splice_region_variant | EXD3 | NM_017820.4 | c.1040C>T | p.Ala347Val |
| #8 | missense_variant,upstream_gene_variant | MAGED2,SNORA11 | NM_014599.5,NR_002953.1 | c.634T>C,n.-1T>C | p.Ser212Pro,. |
| #8 | synonymous_variant | TRO | NM_001039705.2 | c.2022G>T | p.Val674Val |
| #8 | missense_variant | MAMLD1 | NM_001177465.2 | c.2378C>T | p.Ser793Leu |
| #9 | intron_variant | TNR | NM_003285.2 | c.1964-69T>A | . |
| #9 | missense_variant | HMCN1 | NM_031935.2 | c.6124A>C | p.Ser2042Arg |
| #9 | intron_variant | WDR37 | NM_014023.3 | c.650-10C>T | . |
| #9 | non_coding_exon_variant | CCNYL2 | NR_103829.1 | n.1117A>C | . |
| #9 | intron_variant | LTBR | NM_002342.2 | c.802-51A>G | . |
| #9 | intergenic_region | DDX12P-KLRB1 | DDX12P-KLRB1 | . | . |
| #9 | missense_variant | MYF5 | NM_005593.2 | c.100C>T | p.Pro34Ser |
| #9 | intron_variant | TBC1D4 | NM_014832.3 | c.3317-10C>T | . |
| #9 | synonymous_variant | JAG2 | NM_002226.4 | c.1704C>A | p.Gly568Gly |
| #9 | intergenic_region | SLC7A5P2-LOC101927814 | SLC7A5P2-LOC101927814 | . | . |
| #9 | intron_variant | PRKCB | NM_002738.6 | c.1331+102C>T | . |
| #9 | missense_variant | CTNS | NM_001031681.2 | c.861G>T | p.Met287Ile |
| #9 | intron_variant | TOP2A | NM_001067.3 | c.1844-43G>C | . |
| #9 | missense_variant | CDC27 | NM_001114091.2 | c.794G>A | p.Gly265Asp |
| #9 | upstream_gene_variant,intron_variant | LOC100506325,PNPO | NR_103856.1,NM_018129.3 | n.-1G>A,c.418-27C>T | .,. |
| #9 | missense_variant | DENND1C | NM_024898.3 | c.2263C>G | p.Leu755Val |
| #9 | missense_variant | RYR1 | NM_000540.2 | c.15053G>A | p.Arg5018Lys |
| #9 | missense_variant | ZIM3 | NM_052882.1 | c.1335A>C | p.Lys445Asn |
| #9 | missense_variant | TLX2 | NM_016170.4 | c.721C>T | p.Arg241Trp |
| #9 | missense_variant | MAP2 | NM_002374.3 | c.3135A>C | p.Gln1045His |
| #9 | missense_variant | MAP2 | NM_002374.3 | c.3146A>C | p.Lys1049Thr |
| #9 | intron_variant | ATG4B | NM_013325.4 | c.538+104C>T | . |
| #9 | intron_variant | UQCC1 | NM_018244.4 | c.652-2379C>T | . |
| #9 | missense_variant,intron_variant | PAXBP1,PAXBP1-AS1 | NM_016631.3,NR_038879.1 | c.2518C>G,n.2618-900G>C | p.Leu840Val,. |
| #9 | missense_variant | LYZL4 | NM_144634.2 | c.167A>G | p.Lys56Arg |
| #9 | missense_variant | ERICH6 | NM_152394.3 | c.961G>A | p.Glu321Lys |
| #9 | intron_variant,intron_variant | ZNF595,ZNF718 | NM_182524.3,NM_001039127.5 | c.226+12T>A,c.226+12T>A | .,. |
| #9 | intron_variant | KDR | NM_002253.2 | c.2972-22A>T | . |
| #9 | intron_variant | FRG1 | NM_004477.2 | c.741-50T>C | . |
| #9 | intron_variant | KLC4 | NM_201523.2 | c.1210-11C>G | . |
| #9 | missense_variant | SLC35F1 | NM_001029858.3 | c.1030T>C | p.Phe344Leu |
| #9 | upstream_gene_variant,upstream_gene_variant,intron_variant | RASA4CP,DBNL,LINC00957 | NR_024116.2,NM_001122956.1,NR_015401.2 | n.-1A>G,c.-99T>C,n.1445-393T>C | .,.,. |
| #9 | missense_variant,missense_variant | RASA4,RASA4B | NM_006989.5,NM_001277335.1.2 | c.422A>G,c.422A>G | p.Glu141Gly,p.Glu141Gly |
| #9 | synonymous_variant | DCAF12 | NM_015397.3 | c.48G>A | p.Pro16Pro |
| #9 | synonymous_variant | CTSV | NM_001201575.1 | c.852C>T | p.Tyr284Tyr |
| #9 | 5_prime_UTR_premature_start_codon_gain_variant,5_prime_UTR_variant,non_coding_exon_variant | TNFRSF14,TNFRSF14,LOC115110 | NM_003820.3,NM_003820.3,NR_037844.2 | c.-192C>G,c.-192C>G,n.259G>C | .,.,. |
| #9 | splice_region_variant&intron_variant | TNFRSF25 | NM_148965.1 | c.952+5G>C | . |
| #9 | intron_variant | EIF4G3 | NM_001198801.1 | c.546+69C>G | . |
| #9 | intron_variant | TSPAN1 | NM_005727.3 | c.-142+1005G>A | . |
| #9 | intron_variant | AK5 | NM_174858.2 | c.699+15809A>G | . |
| #9 | missense_variant | ATP8B2 | NM_020452.3 | c.856A>G | p.Thr286Ala |
| #9 | missense_variant | SPTA1 | NM_003126.2 | c.5195A>C | p.Lys1732Thr |
| #9 | missense_variant | ITLN1 | NM_017625.2 | c.776G>A | p.Cys259Tyr |
| #9 | intron_variant | RCSD1 | NM_052862.3 | c.1219-87C>G | . |
| #9 | missense_variant | PRRC2C | NM_015172.3 | c.5465C>T | p.Ser1822Phe |
| #9 | missense_variant | ZNF648 | NM_001009992.1 | c.1571G>A | p.Arg524Gln |
| #9 | missense_variant | NUAK2 | NM_030952.1 | c.1105G>C | p.Glu369Gln |
| #9 | missense_variant | TARBP1 | NM_005646.3 | c.1519G>A | p.Gly507Arg |
| #9 | stop_gained | C1orf101 | NM_001130957.1 | c.2803C>T | p.Arg935* |
| #9 | missense_variant | PTER | NM_001001484.2 | c.589C>G | p.Pro197Ala |
| #9 | synonymous_variant | KIAA1217 | NM_019590.4 | c.2685G>A | p.Gln895Gln |
| #9 | missense_variant | RTKN2 | NM_145307.3 | c.1240C>G | p.Arg414Gly |
| #9 | intron_variant | TBATA | NM_152710.2 | c.970+232C>T | . |
| #9 | stop_gained | PDCD4 | NM_014456.4 | c.124G>T | p.Gly42* |
| #9 | stop_gained | PDZD8 | NM_173791.3 | c.1736C>G | p.Ser579* |
| #9 | synonymous_variant | C10orf91 | NM_173541.2 | c.249G>A | p.Gln83Gln |
| #9 | stop_gained | OR52B2 | NM_001004052.1 | c.457C>T | p.Arg153* |
| #9 | missense_variant | PHF21A | NM_001101802.1 | c.1955C>T | p.Pro652Leu |
| #9 | intragenic_variant | DGKZ | DGKZ | . | . |
| #9 | missense_variant | OR8H2 | NM_001005200.1 | c.329C>A | p.Ala110Asp |
| #9 | intron_variant | TREH | NM_007180.2 | c.735-51G>T | . |
| #9 | splice_region_variant&intron_variant | ADAMTS15 | NM_139055.2 | c.2079-6C>T | . |
| #9 | missense_variant | IGSF9B | NM_001277285.1 | c.2685G>C | p.Glu895Asp |
| #9 | intergenic_region | DDX12P-KLRB1 | DDX12P-KLRB1 | . | . |
| #9 | splice_region_variant&intron_variant | MUC19 | NM_173600.2 | c.5702-5A>C | . |
| #9 | intron_variant | AQP6 | NM_001652.3 | c.642+11C>T | . |
| #9 | synonymous_variant | ESPL1 | NM_012291.4 | c.1926G>T | p.Thr642Thr |
| #9 | missense_variant | IKZF4 | NM_022465.3 | c.806G>A | p.Arg269Gln |
| #9 | missense_variant | NAB2 | NM_005967.3 | c.1384G>C | p.Glu462Gln |
| #9 | intergenic_region | ASCL1-C12orf42 | ASCL1-C12orf42 | . | . |
| #9 | intron_variant | NOS1 | NM_001204218.1 | c.3507+18G>A | . |
| #9 | 3_prime_UTR_variant | PSPC1 | NM_001042414.2 | c.*22T>C | . |
| #9 | synonymous_variant | XPO4 | NM_022459.4 | c.3084C>A | p.Ala1028Ala |
| #9 | missense_variant | GPR12 | NM_005288.3 | c.161C>T | p.Ser54Leu |
| #9 | missense_variant | MTUS2 | NM_001033602.2 | c.2222A>C | p.Lys741Thr |
| #9 | 5_prime_UTR_variant | MYO16 | NM_001198950.1 | c.-29G>A | . |
| #9 | missense_variant | MYH7 | NM_000257.3 | c.974A>G | p.Asp325Gly |
| #9 | synonymous_variant,upstream_gene_variant | DHRS4,DHRS4-AS1 | NM_021004.3,NR_023921.2 | c.231G>A,n.-1C>T | p.Gln77Gln,. |
| #9 | missense_variant&splice_region_variant,upstream_gene_variant | DHRS4,DHRS4-AS1 | NM_021004.3,NR_023921.2 | c.305C>T,n.-1G>A | p.Thr102Met,. |
| #9 | stop_gained,intron_variant | PRKD1,MIR548AI | NM_002742.2,NR_039672.1 | c.763C>T,n.29+66536C>T | p.Arg255*,. |
| #9 | intergenic_region | EGLN3-SPTSSA | EGLN3-SPTSSA | . | . |
| #9 | intron_variant | RBM25 | NM_021239.2 | c.156+30G>C | . |
| #9 | splice_donor_variant&intron_variant | PPP4R4 | NM_058237.1 | c.2197+1G>A | . |
| #9 | upstream_gene_variant,intergenic_region | SNORD114-31,SNORD114-30-SNORD114-31 | NR_003224.1,SNORD114-30-SNORD114-31 | n.-1G>A,. | .,. |
| #9 | upstream_gene_variant,upstream_gene_variant,intergenic_region | MIR656,MEG9,MIR410-MIR656 | NR_030392.1,NR_047664.1,MIR410-MIR656 | n.-1G>A,n.-1G>A,. | .,.,. |
| #9 | missense_variant | JAG2 | NM_002226.4 | c.2057G>A | p.Arg686His |
| #9 | missense_variant | BCL2L10 | NM_020396.2 | c.226G>A | p.Glu76Lys |
| #9 | missense_variant | CYP11A1 | NM_000781.2 | c.574G>A | p.Gly192Arg |
| #9 | missense_variant | CSPG4 | NM_001897.4 | c.1321G>A | p.Glu441Lys |
| #9 | missense_variant | EFTUD1 | NM_024580.5 | c.278C>G | p.Ser93Cys |
| #9 | missense_variant | MESP1 | NM_018670.3 | c.260C>A | p.Ala87Asp |
| #9 | intron_variant | BLM | NM_000057.3 | c.2074+14G>A | . |
| #9 | missense_variant | CAPN15 | NM_005632.2 | c.169A>T | p.Asn57Tyr |
| #9 | synonymous_variant | CAPN15 | NM_005632.2 | c.175C>T | p.Leu59Leu |
| #9 | 5_prime_UTR_variant,upstream_gene_variant | NHLRC4,PIGQ | NM_001301159.1,NM_148920.2 | c.-455G>T,c.-139G>T | .,. |
| #9 | intron_variant | PIGQ | NM_148920.2 | c.1531+335G>C | . |
| #9 | missense_variant | CHD9 | NM_025134.4 | c.244G>A | p.Val82Ile |
| #9 | upstream_gene_variant,intron_variant,intron_variant | EMC6,TAX1BP3,P2RX5-TAX1BP3 | NM_001014764.2,NM_014604.3,NR_037928.1 | c.-340G>C,c.159+30C>G,n.5214+30C>G | .,.,. |
| #9 | splice_region_variant&intron_variant | XAF1 | NM_017523.3 | c.226-3C>A | . |
| #9 | stop_gained | TP53 | NM_000546.5 | c.916C>T | p.Arg306* |
| #9 | intron_variant | TOP3A | NM_004618.3 | c.1711+10G>A | . |
| #9 | synonymous_variant | EVPLL | NM_001145127.1 | c.888A>G | p.Pro296Pro |
| #9 | missense_variant | AATF | NM_012138.3 | c.142G>A | p.Gly48Ser |
| #9 | missense_variant | ERBB2 | NM_004448.3 | c.994G>C | p.Glu332Gln |
| #9 | upstream_gene_variant,intron_variant | MIR6867,RAPGEFL1 | NR_106927.1,NM_016339.3 | n.-1C>G,c.216-76C>G | .,. |
| #9 | missense_variant | SRSF1 | NM_006924.4 | c.43G>C | p.Asp15His |
| #9 | stop_gained&splice_region_variant | LOXHD1 | NM_144612.6 | c.1270A>T | p.Lys424* |
| #9 | missense_variant | RTTN | NM_173630.3 | c.199C>G | p.Leu67Val |
| #9 | missense_variant | TCF3 | NM_003200.3 | c.1030C>T | p.His344Tyr |
| #9 | intron_variant | FBN3 | NM_032447.3 | c.6032-31C>A | . |
| #9 | missense_variant | FFAR3 | NM_005304.3 | c.154G>A | p.Val52Met |
| #9 | missense_variant | PROSER3 | NM_001039887.2 | c.514G>C | p.Ala172Pro |
| #9 | missense_variant | CLASRP | NM_007056.2 | c.584C>T | p.Ser195Leu |
| #9 | missense_variant | FBXO46 | NM_001080469.1 | c.1310C>T | p.Ala437Val |
| #9 | missense_variant | ZNF528 | NM_032423.2 | c.949A>C | p.Lys317Gln |
| #9 | intergenic_region | FAM90A27P-BIRC8 | FAM90A27P-BIRC8 | . | . |
| #9 | missense_variant | TMC4 | NM_001145303.2 | c.745C>T | p.Arg249Cys |
| #9 | synonymous_variant,upstream_gene_variant | TSEN34,MBOAT7 | NM_001282333.1,NM_024298.4 | c.933A>G,c.-549T>C | p.Val311Val,. |
| #9 | synonymous_variant | FAM71E2 | NM_001145402.1 | c.528G>A | p.Pro176Pro |
| #9 | synonymous_variant | USP29 | NM_020903.2 | c.2571C>T | p.Asn857Asn |
| #9 | intron_variant | RNF181 | NM_016494.3 | c.218-64C>T | . |
| #9 | synonymous_variant | KIAA1211L | NM_207362.2 | c.1872C>T | p.His624His |
| #9 | missense_variant | AFF3 | NM_001025108.1 | c.116G>A | p.Ser39Asn |
| #9 | intron_variant | ZC3H6 | NM_198581.2 | c.214-13C>T | . |
| #9 | synonymous_variant | MYO7B | NM_001080527.1 | c.231C>T | p.Asn77Asn |
| #9 | missense_variant | LRP1B | NM_018557.2 | c.7603C>T | p.His2535Tyr |
| #9 | missense_variant | NEB | NM_001271208.1 | c.21175C>G | p.Leu7059Val |
| #9 | missense_variant | TTC21B | NM_024753.4 | c.3121G>C | p.Asp1041His |
| #9 | splice_region_variant&intron_variant | STK39 | NM_013233.2 | c.1243-8C>T | . |
| #9 | splice_donor_variant&intron_variant | CPS1 | NM_001122633.2 | c.3354+1G>C | . |
| #9 | splice_region_variant&intron_variant | ABCA12 | NM_173076.2 | c.317+7G>T | . |
| #9 | missense_variant | SP100 | NM_001080391.1 | c.628G>A | p.Glu210Lys |
| #9 | missense_variant | PLCB4 | NM_000933.3 | c.3149T>G | p.Leu1050Arg |
| #9 | synonymous_variant | RRBP1 | NM_001042576.1 | c.658C>T | p.Leu220Leu |
| #9 | intragenic_variant | DEFB119 | DEFB119 | . | . |
| #9 | synonymous_variant,upstream_gene_variant | REM1,LINC00028 | NM_014012.5,NR_024358.1 | c.681G>A,n.-1G>A | p.Thr227Thr,. |
| #9 | 3_prime_UTR_variant,upstream_gene_variant | ID1,MIR3193 | NM_002165.3,NR_036161.1 | c.*38A>G,n.-1A>G | .,. |
| #9 | missense_variant&splice_region_variant | MYLK2 | NM_033118.3 | c.1423C>G | p.Leu475Val |
| #9 | missense_variant | ZMYND8 | NM_001281775.2 | c.1664C>T | p.Ser555Leu |
| #9 | missense_variant | TCFL5 | NM_006602.2 | c.406G>C | p.Glu136Gln |
| #9 | stop_gained | TCFL5 | NM_006602.2 | c.214G>T | p.Glu72* |
| #9 | intron_variant | APP | NM_000484.3 | c.1224+9G>C | . |
| #9 | synonymous_variant | MEI1 | NM_152513.3 | c.1020C>T | p.Leu340Leu |
| #9 | intron_variant | SHANK3 | NM_033517.1 | c.2068+84G>A | . |
| #9 | synonymous_variant | ACR | NM_001097.2 | c.459G>A | p.Ser153Ser |
| #9 | missense_variant | FGD5 | NM_152536.3 | c.1386G>C | p.Leu462Phe |
| #9 | intron_variant | LZTFL1 | NM_020347.3 | c.128+28A>C | . |
| #9 | synonymous_variant | XCR1 | NM_001024644.1 | c.96C>T | p.Leu32Leu |
| #9 | intron_variant | ZNF589 | NM_016089.2 | c.96+123G>A | . |
| #9 | upstream_gene_variant,intron_variant | CELSR3,CELSR3-AS1 | NM_001407.2,NR_111921.1 | c.-282G>C,n.46+349C>G | .,. |
| #9 | intron_variant | DOCK3 | NM_004947.4 | c.4108-16C>T | . |
| #9 | missense_variant,upstream_gene_variant | H1FX,H1FX-AS1 | NM_006026.3,NR_026991.1 | c.233C>G,n.-1G>C | p.Pro78Arg,. |
| #9 | missense_variant | PIK3CA | NM_006218.2 | c.3140A>T | p.His1047Leu |
| #9 | intron_variant | LIMCH1 | NM_014988.3 | c.936-9367G>C | . |
| #9 | intron_variant | LNX1 | NM_001126328.2 | c.380+15693C>G | . |
| #9 | missense_variant&splice_region_variant | KIAA1109 | NM_015312.3 | c.5923G>C | p.Asp1975His |
| #9 | missense_variant | PCDH10 | NM_032961.1 | c.1541C>A | p.Thr514Asn |
| #9 | intron_variant | ARHGAP10 | NM_024605.3 | c.2272+22G>A | . |
| #9 | missense_variant | TCF7 | NM_003202.3 | c.340A>G | p.Ser114Gly |
| #9 | missense_variant | GEMIN5 | NM_015465.4 | c.2885T>A | p.Leu962Gln |
| #9 | intron_variant | HNRNPH1 | NM_001257293.1 | c.-31-26C>G | . |
| #9 | missense_variant&splice_region_variant,intron_variant | LOC100130357,PHACTR1 | NM_001242698.1,NM_001242648.1 | c.143G>A,c.1510-1871C>T | p.Arg48Gln,. |
| #9 | intron_variant | ABHD16A | NM_021160.2 | c.741+51A>C | . |
| #9 | missense_variant,missense_variant,missense_variant | C4A,C4B,C4B_2 | NM_007293.2.4,NM_001002029.3,NM_001242823.2.8 | c.2743G>T,c.2743G>T,c.2743G>T | p.Ala915Ser,p.Ala915Ser,p.Ala915Ser |
| #9 | missense_variant,intron_variant | LOC441155,EYS | NM_001271675.1,NM_001292009.1 | c.899A>G,c.1767-7301T>C | p.Lys300Arg,. |
| #9 | splice_region_variant&intron_variant | C6orf163 | NM_001010868.2 | c.555-3T>C | . |
| #9 | missense_variant | ASCC3 | NM_006828.3 | c.5521A>T | p.Ser1841Cys |
| #9 | missense_variant | TRDN | NM_006073.3 | c.1754G>A | p.Arg585Gln |
| #9 | intron_variant | MOXD1 | NM_015529.3 | c.264+8978G>T | . |
| #9 | missense_variant | SASH1 | NM_015278.3 | c.2615C>T | p.Thr872Met |
| #9 | missense_variant | PACRG | NM_152410.2 | c.479G>A | p.Arg160Gln |
| #9 | intron_variant | TRG-AS1 | NR_040085.1 | n.435+8401G>C | . |
| #9 | intron_variant | AMPH | NM_001635.3 | c.1158+93G>T | . |
| #9 | non_coding_exon_variant | GUSBP10 | NR_030766.1 | n.145G>A | . |
| #9 | missense_variant | DPP6 | NM_130797.3 | c.191G>A | p.Gly64Asp |
| #9 | intron_variant | XKR4 | NM_052898.1 | c.806+37004G>A | . |
| #9 | missense_variant | PI15 | NM_015886.3 | c.685T>C | p.Cys229Arg |
| #9 | intron_variant | CYP11B1 | NM_000497.3 | c.396-612A>G | . |
| #9 | synonymous_variant | KIAA1161 | NM_020702.4 | c.1497G>A | p.Ser499Ser |
| #9 | non_coding_exon_variant | FAM205B | NR_024481.1 | n.914G>A | . |
| #9 | synonymous_variant | MAMDC2 | NM_153267.4 | c.273G>A | p.Ser91Ser |
| #9 | missense_variant | MAP3K15 | NM_001001671.3 | c.1531T>G | p.Phe511Val |
| #9 | missense_variant | KLHL34 | NM_153270.1 | c.1787A>G | p.Asp596Gly |
| #9 | synonymous_variant | PORCN | NM_203475.2 | c.432C>T | p.Gly144Gly |
| #9 | non_coding_exon_variant | XIST | NR_001564.2 | n.1199C>T | . |
| #9 | intron_variant | ATRX | NM_000489.4 | c.5786+68T>C | . |
| #9 | synonymous_variant,upstream_gene_variant | COX7B,MAGT1 | NM_001866.2,NM_032121.5 | c.21C>T,c.-63G>A | p.Ser7Ser,. |
| #9 | missense_variant | P2RY10 | NM_014499.2 | c.789T>A | p.Phe263Leu |
| #9 | 5_prime_UTR_variant | CYLC1 | NM_021118.2 | c.-16C>A | . |
| #9 | missense_variant,missense_variant | NXF2B,NXF2 | NM_001099686.2.2,NM_022053.3 | c.602C>T,c.602C>T | p.Ala201Val,p.Ala201Val |
| #9 | synonymous_variant | HS6ST2 | NM_001077188.1 | c.1254T>C | p.Phe418Phe |
| #9 | synonymous_variant | HS6ST2 | NM_001077188.1 | c.1236C>T | p.Gly412Gly |
| #9 | missense_variant | ATP11C | NM_173694.4 | c.2303A>G | p.Asp768Gly |
| #9 | stop_gained | UBE2NL | NM_001012989.2 | c.37C>T | p.Gln13* |
| #9 | synonymous_variant,upstream_gene_variant | GDI1,FAM50A | NM_001493.2,NM_004699.3 | c.936C>T,c.-111C>T | p.Asn312Asn,. |

Supplementary Table S6 List of mutations (INDELs) in metastatic ovarian tumours.

|  | **Var_type** | **Consequences** | **Gene** | **Transcript_id** | **HGVS_P** |
| --- | --- | --- | --- | --- | --- |
| #5 | INS | splice_region_variant&intron_variant | SCNN1D | NM_001130413.3 | . |
| #5 | DEL | intron_variant | CHIA | NM_201653.3 | . |
| #5 | DEL | intron_variant | RABGAP1L | NM_014857.4 | . |
| #5 | INS | intron_variant | AIDA | NM_022831.2 | . |
| #5 | DEL | intron_variant | SPAG6 | NM_012443.3 | . |
| #5 | INS | intron_variant | PARD3 | NM_019619.3 | . |
| #5 | DEL | intron_variant | SUPV3L1 | NM_003171.3 | . |
| #5 | DEL | intron_variant | DDX12P | NR_033399.1 | . |
| #5 | DEL | intron_variant | UBE2N | NM_003348.3 | . |
| #5 | DEL | intron_variant | KIAA1033 | NM_001293640.1 | . |
| #5 | DEL | intron_variant | PHF11 | NM_001040443.1 | . |
| #5 | DEL | intron_variant | UNC13C | NM_001080534.1 | . |
| #5 | INS | intron_variant | BNIP2 | NM_004330.2 | . |
| #5 | DEL | 5_prime_UTR_variant | ORAI3 | NM_152288.2 | . |
| #5 | DEL | intron_variant | CIRH1A | NM_032830.2 | . |
| #5 | INS | intron_variant | NUMBL | NM_004756.4 | . |
| #5 | DEL | intron_variant | IL1RL1 | NM_016232.4 | . |
| #5 | DEL | intron_variant | PAX3 | NM_181459.3 | . |
| #5 | INS | intron_variant | RPN2 | NM_002951.3 | . |
| #5 | DEL | intron_variant,intron_variant | BLCAP,NNAT | NM_001167820.1,NM_005386.2 | .,. |
| #5 | DEL | intron_variant | KCNQ2 | NM_172107.2 | . |
| #5 | DEL | intron_variant | SAMM50 | NM_015380.4 | . |
| #5 | DEL | intron_variant | ROBO1 | NM_002941.3 | . |
| #5 | INS | intron_variant | PPP3CA | NM_000944.4 | . |
| #5 | DEL | intergenic_region | IRX1-LOC101929153 | IRX1-LOC101929153 | . |
| #5 | DEL | intron_variant | CCDC152 | NM_001134848.1 | . |
| #5 | INS | intron_variant | ANKRD32 | NM_032290.3 | . |
| #5 | DEL | intron_variant | NUP43 | NM_198887.2 | . |
| #5 | DEL | disruptive_inframe_deletion | THSD7A | NM_015204.2 | p.Pro31_Leu34del |
| #5 | INS | intron_variant | LMBR1 | NM_022458.3 | . |
| #5 | INS | intron_variant | NCOA2 | NM_006540.2 | . |
| #5 | INS | intron_variant | TRAPPC9 | NM_031466.6 | . |
| #5 | DEL | intron_variant | EDA | NM_001399.4 | . |
| #5 | INS | intron_variant | SNRNP40 | NM_004814.2 | . |
| #5 | DEL | intron_variant | C1orf168 | NM_001004303.4 | . |
| #5 | INS | intron_variant | LYST | NM_000081.3 | . |
| #5 | DEL | intron_variant | GPR158 | NM_020752.2 | . |
| #5 | DEL | intron_variant | TRPM5 | NM_014555.3 | . |
| #5 | DEL | intron_variant | NUP98 | NM_016320.4 | . |
| #5 | DEL | intron_variant | OTOGL | NM_173591.3 | . |
| #5 | INS | intron_variant | CAMKK2 | NM_001270485.1 | . |
| #5 | DEL | intron_variant | CCNA1 | NM_003914.3 | . |
| #5 | DEL | splice_region_variant&intron_variant | COG6 | NM_020751.2 | . |
| #5 | DEL | intron_variant | SOS2 | NM_006939.2 | . |
| #5 | DEL | intron_variant | TRIP11 | NM_004239.3 | . |
| #5 | DEL | splice_region_variant&intron_variant,intron_variant | TP53BP1,RNU6-28P | NM_001141980.1,NR_046489.1.2 | .,. |
| #5 | INS | intron_variant | CDH13 | NM_001220488.1 | . |
| #5 | DEL | 5_prime_UTR_variant,upstream_gene_variant,intergenic_region,transcript | RHOT1,RHOT1,LRRC37B-RHOT1,RHOT1 | NM_001033568.2,NM_001033568.2,LRRC37B-RHOT1,NM_001033568.2 | .,.,.,. |
| #5 | DEL | intron_variant | PSMD11 | NM_001270482.1 | . |
| #5 | DEL | upstream_gene_variant,intergenic_region | KLK1,KLK1-KLK15 | NM_002257.3,KLK1-KLK15 | .,. |
| #5 | DEL | intergenic_region | GCKR-C2orf16 | GCKR-C2orf16 | . |
| #5 | DEL | intergenic_region | SLC4A3-MIR4268 | SLC4A3-MIR4268 | . |
| #5 | DEL | splice_region_variant&intron_variant,upstream_gene_variant | CRELD2,ALG12 | NM_001135101.2,NM_024105.3 | .,. |
| #5 | DEL | intron_variant | KLF3 | NM_016531.5 | . |
| #5 | DEL | intron_variant | TBCK | NM_001163435.2 | . |
| #5 | DEL | 3_prime_UTR_variant | PLRG1 | NM_002669.3 | . |
| #5 | DEL | intergenic_region | IRX1-LOC101929153 | IRX1-LOC101929153 | . |
| #5 | INS | intron_variant | AGER | NM_001206929.1.2 | . |
| #5 | DEL | intron_variant | RNASET2 | NM_003730.4 | . |
| #5 | DEL | intergenic_region | SMOC2-THBS2 | SMOC2-THBS2 | . |
| #5 | DEL | intron_variant | IGFBP3 | NM_001013398.1 | . |
| #5 | INS | intron_variant | SNX16 | NM_022133.3 | . |
| #5 | DEL | intron_variant | LRRC69 | NM_001129890.1 | . |
| #5 | DEL | intron_variant | CSMD3 | NM_198123.1 | . |
| #5 | INS | intron_variant | ADAMTSL1 | NM_001040272.5 | . |
| #5 | DEL | 3_prime_UTR_variant | SYTL4 | NM_001129896.2 | . |
| #8 | DEL | intron_variant | DOCK7 | NM_001271999.1 | . |
| #8 | DEL | intron_variant | MFSD4 | NM_181644.4 | . |
| #8 | INS | intron_variant | KBTBD3 | NM_152433.3 | . |
| #8 | INS | intron_variant | CACNA2D4 | NM_172364.4 | . |
| #8 | DEL | intron_variant | GLIPR1 | NM_006851.2 | . |
| #8 | INS | intron_variant | SNW1 | NM_012245.2 | . |
| #8 | DEL | intron_variant | GANC | NM_198141.2 | . |
| #8 | DEL | intron_variant | LOC653786 | NR_003676.3 | . |
| #8 | DEL | intron_variant | ADAMTS18 | NM_199355.2 | . |
| #8 | INS | intron_variant | FANCA | NM_000135.2 | . |
| #8 | DEL | intron_variant | SERPINF1 | NM_002615.5 | . |
| #8 | DEL | splice_region_variant&intron_variant | C19orf71 | NM_001135580.1 | . |
| #8 | INS | intron_variant | VWA3B | NM_144992.4 | . |
| #8 | DEL | intron_variant | HM13 | NM_178581.2 | . |
| #8 | DEL | intron_variant | KCNQ2 | NM_172107.2 | . |
| #8 | DEL | intron_variant | DCLK2 | NM_001040261.4 | . |
| #8 | INS | intron_variant | SLC6A7 | NM_014228.3 | . |
| #8 | INS | 5_prime_UTR_variant | HLA-DRB1 | NM_002124.3 | . |
| #8 | DEL | intron_variant | DNAH8 | NM_001206927.1 | . |
| #8 | DEL | intron_variant | SYNE1 | NM_182961.3 | . |
| #8 | INS | intron_variant | TMEM196 | NM_152774.3 | . |
| #8 | DEL | 3_prime_UTR_variant | NEIL2 | NM_001135746.1 | . |
| #8 | INS | frameshift_variant,non_coding_exon_variant | TRPA1,LOC100132891 | NM_007332.2,NR_033652.1 | p.Gly505fs,. |
| #8 | DEL | intron_variant | KAL1 | NM_000216.2 | . |
| #8 | DEL | intron_variant | CXorf22 | NM_152632.3 | . |
| #8 | INS | non_coding_exon_variant,non_coding_exon_variant | TSIX,XIST | NR_003255.2,NR_001564.2 | .,. |
| #8 | DEL | intron_variant | HP1BP3 | NM_016287.3 | . |
| #8 | INS | 5_prime_UTR_variant | KCNK1 | NM_002245.3 | . |
| #8 | INS | intron_variant | ANKRD26 | NM_014915.2 | . |
| #8 | DEL | intron_variant | ANKRD26 | NM_014915.2 | . |
| #8 | DEL | upstream_gene_variant,upstream_gene_variant,intron_variant | HSPB2,HSPB2-C11orf52,CRYAB | NM_001541.3,NR_037651.1,NM_001289807.1 | .,.,. |
| #8 | INS | intron_variant | WIF1 | NM_007191.4 | . |
| #8 | INS | upstream_gene_variant,non_coding_exon_variant | TRHDE,TRHDE-AS1 | NM_013381.2,NR_026837.1 | .,. |
| #8 | DEL | 3_prime_UTR_variant,3_prime_UTR_variant | GLIPR1,KRR1 | NM_006851.2,NM_007043.6 | .,. |
| #8 | INS | intron_variant | GOLGA3 | NM_005895.3 | . |
| #8 | INS | intron_variant | LACC1 | NM_001128303.1 | . |
| #8 | INS | intron_variant | KLF5 | NM_001730.4 | . |
| #8 | INS | intron_variant,intron_variant | MYHAS,MYH2 | NR_125367.1,NM_001100112.1 | .,. |
| #8 | DEL | intron_variant | FASN | NM_004104.4 | . |
| #8 | INS | 5_prime_UTR_variant | MAP4K4 | NM_145686.3 | . |
| #8 | DEL | intron_variant | SPEG | NM_005876.4 | . |
| #8 | INS | intron_variant | RPN2 | NM_002951.3 | . |
| #8 | INS | intragenic_variant | COL18A1 | COL18A1 | . |
| #8 | DEL | intron_variant | SLCO4C1 | NM_180991.4 | . |
| #8 | DEL | 3_prime_UTR_variant | PPIC | NM_000943.4 | . |
| #8 | DEL | disruptive_inframe_deletion | AK9 | NM_001145128.2 | p.Glu703del |
| #8 | DEL | intron_variant | ACAT2 | NM_005891.2 | . |
| #8 | DEL | intron_variant | NUGGC | NM_001010906.1 | . |
| #8 | DEL | intron_variant | HOOK3 | NM_032410.3 | . |
| #8 | DEL | intron_variant | KDM4C | NM_015061.3 | . |
| #9 | INS | intron_variant | PADI4 | NM_012387.2 | . |
| #9 | INS | intron_variant | SCYL3 | NM_181093.3 | . |
| #9 | DEL | intron_variant | EDEM3 | NM_025191.3 | . |
| #9 | INS | 5_prime_UTR_variant | KCNMA1 | NM_001161352.1 | . |
| #9 | INS | intron_variant,intron_variant | TRIM6,TRIM6-TRIM34 | NM_001003818.2,NM_001003819.3 | .,. |
| #9 | DEL | intron_variant | GALNT8 | NM_017417.1 | . |
| #9 | DEL | intron_variant | SYT10 | NM_198992.3 | . |
| #9 | DEL | intron_variant | DIP2B | NM_173602.2 | . |
| #9 | DEL | upstream_gene_variant,non_coding_exon_variant | RILPL1,MIR3908 | NM_178314.3,NR_037470.1 | .,. |
| #9 | INS | intergenic_region | LOC338797-SFSWAP | LOC338797-SFSWAP | . |
| #9 | DEL | intron_variant | SPAG9 | NM_001130528.2 | . |
| #9 | DEL | non_coding_exon_variant | LINC00469 | NR_027146.1 | . |
| #9 | DEL | intron_variant,intron_variant | ZNF559,ZNF559-ZNF177 | NM_001202406.1,NM_001172650.2 | .,. |
| #9 | DEL | intron_variant | WDR43 | NM_015131.1 | . |
| #9 | DEL | intergenic_region | CXCR4-THSD7B | CXCR4-THSD7B | . |
| #9 | DEL | upstream_gene_variant,intron_variant | LOC101929225,CRLS1 | NR_110101.1,NM_019095.4 | .,. |
| #9 | DEL | intron_variant | CLASP2 | NM_015097.2 | . |
| #9 | INS | intron_variant | PCDH7 | NM_001173523.1 | . |
| #9 | INS | intron_variant | FRG1 | NM_004477.2 | . |
| #9 | DEL | intron_variant | ADAMTS6 | NM_197941.2 | . |
| #9 | DEL | upstream_gene_variant,intron_variant | FCHSD1,ARAP3 | NM_033449.2,NM_022481.5 | .,. |
| #9 | DEL | intron_variant | HLA-DQA1 | NM_002122.3 | . |
| #9 | INS | intron_variant | TTK | NM_003318.4 | . |
| #9 | DEL | intron_variant | POT1 | NM_015450.2 | . |
| #9 | DEL | intron_variant | CASP2 | NM_032982.3 | . |
| #9 | DEL | intron_variant | SCML2 | NM_006089.2 | . |
| #9 | DEL | intron_variant | ABCA4 | NM_000350.2 | . |
| #9 | DEL | intron_variant | SDHC | NM_003001.3 | . |
| #9 | INS | intron_variant,intron_variant | RNU5F-1,EPRS | NR_002753.5,NM_004446.2 | .,. |
| #9 | INS | intron_variant | TUBGCP2 | NM_001256617.1 | . |
| #9 | INS | intron_variant | OVCH2 | NM_198185.4 | . |
| #9 | INS | intron_variant | DLG2 | NM_001142699.1 | . |
| #9 | INS | intron_variant | PLEKHA5 | NM_001256470.1 | . |
| #9 | DEL | intron_variant | KIF21A | NM_001173464.1 | . |
| #9 | INS | intron_variant | MPP5 | NM_022474.3 | . |
| #9 | DEL | intron_variant,intron_variant | TTC7B,LOC101928909 | NM_001010854.1,NR_110134.1 | .,. |
| #9 | DEL | intron_variant | HDC | NM_002112.3 | . |
| #9 | DEL | intron_variant,intron_variant | MIR548H4,NOX5 | NR_031680.1,NM_024505.3 | .,. |
| #9 | INS | intron_variant | FAM169B | NM_182562.2 | . |
| #9 | DEL | intron_variant | ZSCAN32 | NM_001284527.1 | . |
| #9 | DEL | intron_variant | NDE1 | NM_001143979.1 | . |
| #9 | DEL | splice_region_variant&intron_variant | RPGRIP1L | NM_015272.2 | . |
| #9 | DEL | upstream_gene_variant,intergenic_region | DPH1,RTN4RL1-DPH1 | NM_001383.3,RTN4RL1-DPH1 | .,. |
| #9 | INS | intron_variant | JUP | NM_002230.2 | . |
| #9 | INS | intron_variant | ADAMTS10 | NM_030957.3 | . |
| #9 | INS | intron_variant | NEB | NM_001271208.1 | . |
| #9 | DEL | intron_variant | ICA1L | NM_001288622.1 | . |
| #9 | INS | 5_prime_UTR_variant | PTGIS | NM_000961.3 | . |
| #9 | DEL | inframe_deletion | TAF4 | NM_003185.3 | p.Pro186_Gly187del |
| #9 | DEL | 3_prime_UTR_variant | EEF1A2 | NM_001958.3 | . |
| #9 | DEL | upstream_gene_variant,intron_variant | ARFRP1,ZGPAT | NM_001267547.2,NM_032527.4 | .,. |
| #9 | DEL | intergenic_region | IGLL5-RTDR1 | IGLL5-RTDR1 | . |
| #9 | DEL | intron_variant | SPICE1 | NM_144718.3 | . |
| #9 | INS | intergenic_region | MIR4275-PCDH7 | MIR4275-PCDH7 | . |
| #9 | INS | intron_variant | CWC27 | NM_005869.3 | . |
| #9 | INS | intron_variant,intron_variant | EEF1E1-BLOC1S5,EEF1E1 | NR_037618.1,NM_004280.4 | .,. |
| #9 | DEL | intron_variant | RFX6 | NM_173560.3 | . |
| #9 | DEL | intron_variant | NUP214 | NM_005085.3 | . |
| #9 | INS | upstream_gene_variant,intergenic_region | PTCHD1,PTCHD1-AS-PTCHD1 | NM_173495.2,PTCHD1-AS-PTCHD1 | .,. |
| #9 | INS | intron_variant | BRWD3 | NM_153252.4 | . |
| #9 | INS | 3_prime_UTR_variant | SYTL4 | NM_001129896.2 | . |
| #9 | INS | intron_variant | SPANXN1 | NM_001009614.2 | . |


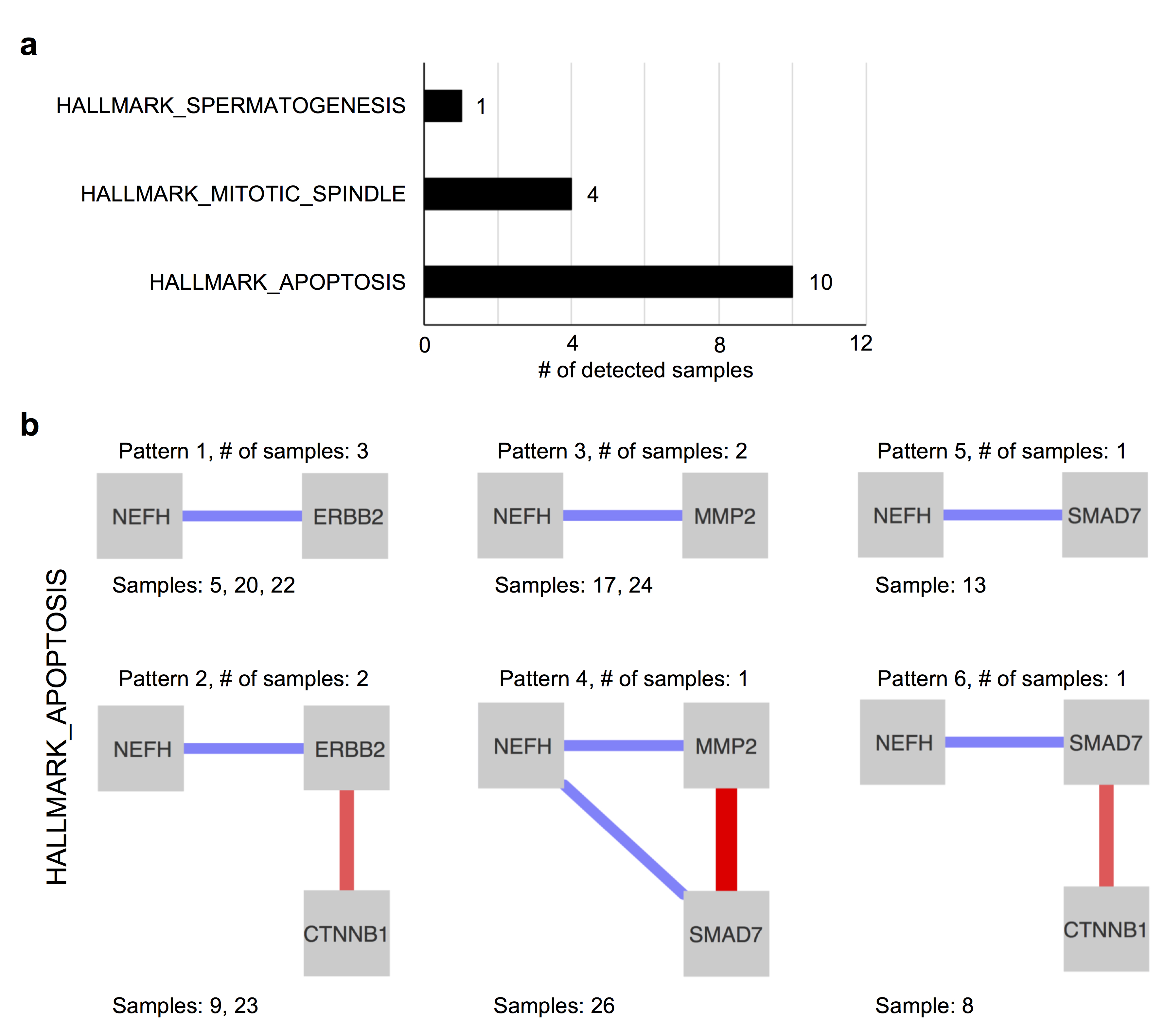


Supplementary Figure S1. The sample-specific networks in the targeted sequencing dataset^1^ showed the apoptosis consistently. (a) Among 26 samples, twenty-three samples were showed significant changes comparing to the TCGA-matched samples, and the three contexts (apoptosis, mitotic spindle, and spermatogenesis) were revealed again (HALLMARK_APOPTOSIS in 10 patients, HALLMARK_MITOTIC_SPINDLE in 4 patients, and HALLMARK_SPERMATOGENESIS in a patient). (b) The 10 samples relating to HAMLLMARK_APOPTOSIS in (a) revealed new significant correlation changes with *NEFH*. Depending on configuration of neighboring nodes, these ten samples categorized into patterns 1 to 6. *NEFH* was associated with *CTNNB1, ERBB2, MMP2*, and *SMAD7*. Colored edges indicate positive (red) and negative (blue) correlations.


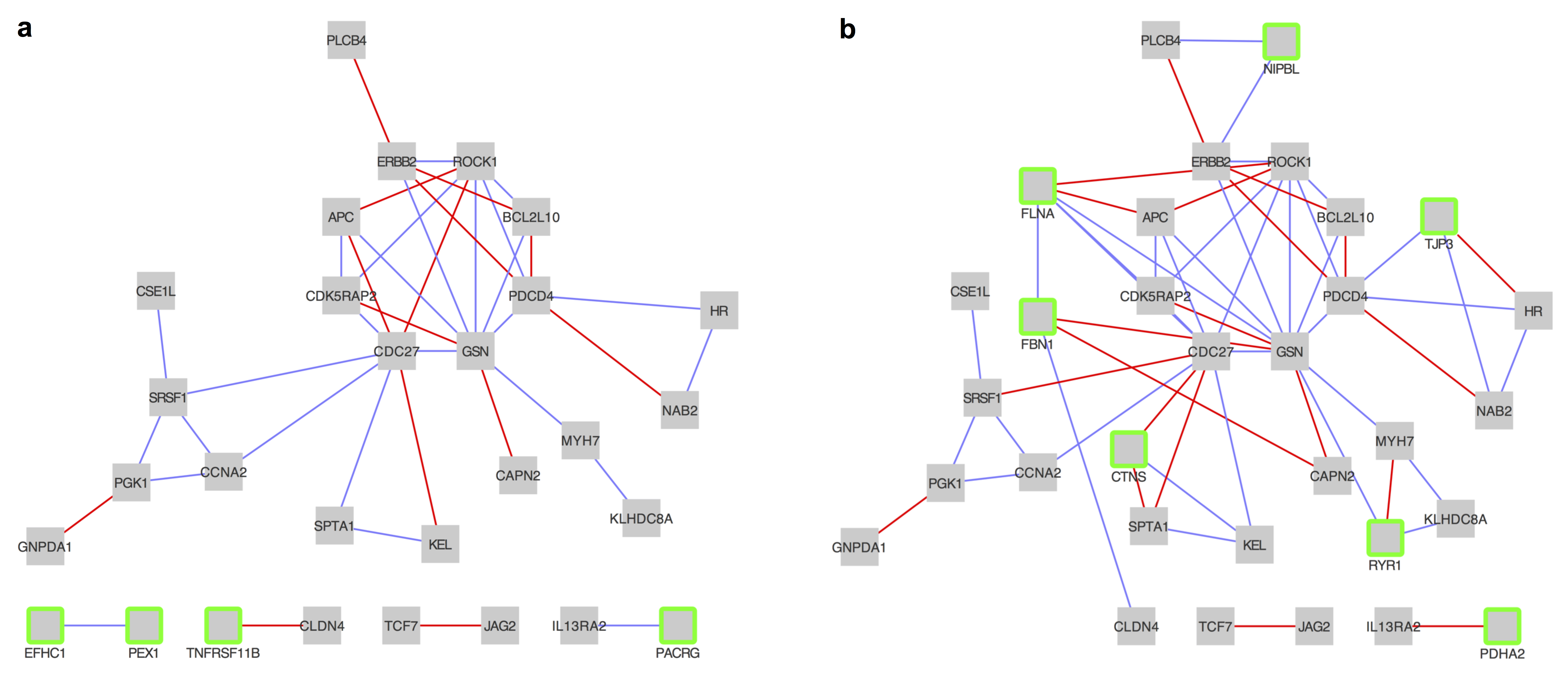


Supplementary Figure S2. Mutational co-occurrence networks showed similar topological structures of primary CRC and metastasized ovarian tumors from patients #5, #8, and #9. (a) The overall configurations between the primary CRC network and (b) the metastasized ovarian network showed similarity but there were minor differences. For example, *APC*-*CDC27* and *CDC27*-*ROCK1* had positive association in the CRC network, but, in the metastasized ovarian tumor network, *APC*-*CDC27* and *CDC27*-*ROCK1* were negatives. However, significantly detected genes (nodes) and their associations (edges) were preserved in the metastasized ovarian network. Green-bounded nodes represented genes exclusively belonging to either the CRC or the metastasized ovarian cancer networks. Grey nodes indicated common genes in both networks. Red edges indicated positive and blue negative correlations.

Supplementary References

1 Crobach, S. *et al.* Somatic mutation profiles in primary colorectal cancers and matching ovarian metastases: Identification of driver and passenger mutations. *J Pathol Clin Res* **2**, 166-174, doi:10.1002/cjp2.45 (2016).

2 Kramer, A., Green, J., Pollard, J., Jr. & Tugendreich, S. Causal analysis approaches in Ingenuity Pathway Analysis. *Bioinformatics* **30**, 523-530, doi:10.1093/bioinformatics/btt703 (2014).
